# The efficacy and safety of anti-Aβ agents for delaying cognitive decline in Alzheimer’s disease: a meta-analysis

**DOI:** 10.3389/fnagi.2023.1257973

**Published:** 2023-11-06

**Authors:** Jiaxuan Li, Xin Wu, Xin Tan, Shixin Wang, Ruisi Qu, Xiaofeng Wu, Zhouqing Chen, Zhong Wang, Gang Chen

**Affiliations:** ^1^Department of Neurosurgery & Brain and Nerve Research Laboratory, The First Affiliated Hospital of Soochow University, Suzhou, Jiangsu Province, China; ^2^Department of Neurology, The Affiliated Suzhou Hospital of Nanjing Medical University, Suzhou Municipal Hospital, Suzhou, Jiangsu Province, China; ^3^Department of Ultrasound, The First Affiliated Hospital of Soochow University, Suzhou, Jiangsu Province, China

**Keywords:** Alzheimer’s disease, cognitive impairment, amyloid-β, monoclonal antibody, γ-secretase inhibitors, BACE-1 inhibitors, intravenous immunoglobulin, γ-secretase modulators

## Abstract

**Background:**

This meta-analysis evaluates the efficacy and safety of amyloid-β (Aβ) targeted therapies for delaying cognitive deterioration in Alzheimer’s disease (AD).

**Methods:**

PubMed, EMBASE, the Cochrane Library, and ClinicalTrials.gov were systematically searched to identify relevant studies published before January 18, 2023.

**Results:**

We pooled 33,689 participants from 42 studies. The meta-analysis showed no difference between anti-Aβ drugs and placebo in the Alzheimer’s Disease Assessment Scale–Cognitive Subscale (ADAS-Cog), and anti-Aβ drugs were associated with a high risk of adverse events [ADAS-Cog: MDs = −0.08 (−0.32 to 0.15), *p* = 0.4785; AEs: RR = 1.07 (1.02 to 1.11), *p* = 0.0014]. Monoclonal antibodies outperformed the placebo in delaying cognitive deterioration as measured by ADAS-Cog, Clinical Dementia Rating–Sum of Boxes (CDR-SB), Mini-Mental State Examination (MMSE) and Alzheimer’s Disease Cooperative Study–Activities of Daily Living (ADCS-ADL), without increasing the risk of adverse events [ADAS-Cog: MDs = −0.55 (−0.89 to 0.21), *p* = 0.001; CDR-SB: MDs = −0.19 (−0.29 to −0.10), *p* < 0.0001; MMSE: MDs = 0.19 (0.00 to 0.39), *p* = 0.05; ADCS-ADL: MDs = 1.26 (0.84 to 1.68), *p* < 0.00001]. Intravenous immunoglobulin and γ-secretase modulators (GSM) increased cognitive decline in CDR-SB [MDs = 0.45 (0.17 to 0.74), *p* = 0.002], but had acceptable safety profiles in AD patients. γ-secretase inhibitors (GSI) increased cognitive decline in ADAS-Cog, and also in MMSE and ADCS-ADL. BACE-1 inhibitors aggravated cognitive deterioration in the outcome of the Neuropsychiatric Inventory (NPI). GSI and BACE-1 inhibitors caused safety concerns. No evidence indicates active Aβ immunotherapy, MPAC, or tramiprosate have effects on cognitive function and tramiprosate is associated with serious adverse events.

**Conclusion:**

Current evidence does not show that anti-Aβ drugs have an effect on cognitive performance in AD patients. However, monoclonal antibodies can delay cognitive decline in AD. Development of other types of anti-Aβ drugs should be cautious.

**Systematic Review Registration:**

PROSPERO (https://www.crd.york.ac.uk/prospero/), identifier CRD42023391596.

## 1. Background

Alzheimer’s disease (AD) is a progressive, irreversible, and fatal neurodegenerative disease associated with decreased cognitive performance. The main risk factor for AD is age, and AD is the fifth leading cause of death in people over 65 years of age (Cummings et al., 2021; Pardo-Moreno et al., 2022). The mortality rate of AD increased from 0.0165% in 1999 to 0.0305% in 2018 and is increasing rapidly upward from 2019 to 2023 (Zhao et al., 2021). AD represents a significant challenge and burden on the public health system, with no effective disease-modifying or preventive therapies available (Alzheimer's Association, 2019). The amyloid-β (Aβ) hypothesis is widely accepted as the primary pathogenesis of AD (Mo et al., 2017). Furthermore, β-amyloidosis and the pathological changes it causes (pathologic tau and neurodegeneration) are considered to be one of the main causes of cognitive decline (Jack et al., 2018).

Over the last 30 years, many therapy strategies targeting AD pathogenesis have been proposed, and much of the work focused on the Aβ cascade hypothesis (ACH) to prevent Aβ accumulation (Hane et al., 2017; Penke et al., 2017). Disease-modifying therapies are currently the most common treatment tested in AD research, with the Aβ target accounting for approximately 15.4% (Cummings et al., 2021). In clinical trials, the main modes of targeting Aβ have been active immunization, passive immunization, and secretase inhibitors (Long and Holtzman, 2019). Active immunization or vaccination removes or prevents Aβ plaques by introducing Aβ peptide fragments, stimulating the patient’s immune response, and actively producing antibodies against Aβ (Mo et al., 2017). Another strategy for Aβ clearance is passive immunotherapy, with monoclonal antibodies as the primary passive immunotherapy for AD (Plascencia-Villa and Perry, 2023). The Food and Drug Administration (FDA) approved aducanumab in 2021, making it the first anti-Aβ monoclonal antibody approved for the treatment of AD, and lecanemab was recently approved as well (Mafi et al., 2022; Larkin, 2023). Aβ is produced by sequential cleavage of Aβ precursor protein (APP) by β-secretase and γ-secretase. BACE1 (β-site APP cleaving enzyme-1) is a unique β-secretase; its absence can prevent the production of Aβ, making BACE1 an important therapeutic target (Haass et al., 2012). Hence, strategies focused on BACE1 inhibitors, γ-secretase inhibitors (GSI) and γ-secretase modulators (GSM) have also been developed for the treatment of AD.

Several previous meta-analyses have investigated the effectiveness of different classes of Aβ-targeted drugs in patients with AD, each based on a limited number of randomized controlled trials (RCTs) and with inconsistent conclusions (Mo et al., 2017; Penninkilampi et al., 2017; Foroutan et al., 2019; Liu and Wang, 2019; Lu et al., 2020; Avgerinos et al., 2021; Lacorte et al., 2022). Therefore, we conducted a meta-analysis of RCTs of all drugs targeting Aβ including monoclonal antibodies, BACE1 inhibitors, active immunotherapy, GSI, intravenous immunoglobulin, GSM, metal-protein–attenuating compounds (MPAC), and tramiprosate. In addition, to investigate the optimal treatment strategy for AD, subgroup analyses were performed to assess the effects of drug class, duration of treatment, and baseline characteristics of patients on outcomes.

## 2. Methods

### 2.1. Study protocol

Before the project started, we drafted a research protocol following the Cochrane Collaboration format (Liberati et al., 2009). The protocol for this systematic review has been registered in PROSPERO (CRD42023391596).

### 2.2. Study selection

We set the inclusion criteria as follows: (a) study type: RCT; (b) language restriction: only available in English; (c) participants: patients who had cognitive impairment due to AD; (d) intervention: Aβ-targeting agents; control: placebo; (e) outcomes: the primary efficacy outcome was Alzheimer’s Disease Assessment Scale–Cognitive Subscale (ADAS-Cog). Secondary efficacy outcomes included Clinical Dementia Rating–Sum of Boxes (CDR-SB), Mini-Mental State Examination (MMSE), Alzheimer’s Disease Cooperative Study–Activities of Daily Living (ADCS-ADL), and Neuropsychiatric Inventory (NPI). Safety outcomes included adverse events (AEs), serious adverse events (SAEs), and death. The included RCTs were requested to supply the primary efficacy outcome. The exclusion criteria were set as follows: (1) study type: retrospective and cohort studies, reviews, conferences, protocols and case reports; (2) participants: patients with dementia not caused by AD; (3) intervention: tau-targeted therapies, lifestyle interventions.

### 2.3. Search strategy

PubMed, EMBASE, the Cochrane Library, and ClinicalTrials.gov were systematically searched to identify relevant studies published before January 18, 2023. The following search strategy was employed: “Amyloid Beta-Peptides” AND “Alzheimer’s disease” in the title, abstract or keywords. The comprehensive search strategy is in the Supplementary Table S1. To ensure a more thorough search, the reference lists of RCTs, relevant systematic reviews, and meta-analyses were independently and manually screened.

### 2.4. Study selection and data collection

Two reviewers (JXL and XW) independently reviewed all titles, abstracts, and full-text articles searched from the four databases, as well as the reference lists of RCTs and relevant systematic reviews or meta-analyses, in accordance with the eligibility criteria mentioned above. Duplicates and research articles for which the full text was unavailable were excluded. Disagreements between the two authors were settled through discussion or, if necessary, by a third author (XT) not involved in data collection. Following selection and evaluation, the following data were extracted from the included RCTs: study characteristics, baseline characteristics and outcome events included for each RCT (Supplementary Table S2); inclusion and exclusion criteria, study design, and all efficacy and safety outcomes are shown in Supplementary Table S3.

### 2.5. Quality assessment

The quality assessment of the included studies was evaluated with reference to the method of Lin et al. (2018). Included studies were assessed through seven items, and a study could receive a total score from 0 to 7. Quality assessment was not used as an exclusion criterion. The results of the quality assessment are presented in the Supplementary Table S4.

### 2.6. Risk of bias

Review Manager 5.3 software was used to assess the risk of bias plot. To evaluate the risk of bias in RCTs, the Cochrane Collaboration’s uniform criteria were used (Higgins et al., 2011), which included selection bias, performance bias, detection bias, attrition bias, reporting bias, and other potential biases. Each bias criterion was classified as “low,” “high,” or “unclear.” JXL and XW conducted the evaluation independently. Disagreements were settled with the help of a third author (XT).

### 2.7. Statistical analysis

R 3.5.3 statistical software and meta-package were used to perform the meta-analysis. We estimated the mean differences (MDs) with 95% confidence interval (CI) for continuous outcomes and risk ratio (RR) with 95% CI for dichotomous outcomes. If a study included multiple intervention groups, we combined the experimental groups into one group, and the means and standard deviation (SD) were combined according to Cochrane Handbook (Shuster, 2011). Heterogeneity was estimated as follows: I (Pardo-Moreno et al., 2022) < 30% indicates “low heterogeneity”; I (Pardo-Moreno et al., 2022) between 30 and 50% suggests “moderate heterogeneity”; I (Pardo-Moreno et al., 2022) > 50% indicates “substantial heterogeneity.” For data with less than 50% heterogeneity, we used a common effects model, and for data with more than 50% heterogeneity, we used a random effects model. We evaluated the possible heterogeneity of treatment effects and the robustness of our findings with subgroup metaanalyses using follow-up time (< 72 weeks and ≥ 72 weeks), types of drugs, and degree of cognitive impairment of the included patients (early AD only) as covariates. We defined early AD as patients with mild cognitive impairment, i.e., MMSE scores >20. We also performed sensitivity analyses by removing each RCT. Two-tailed tests were performed for all the analyses, and a *p* value <0.05 was considered statistically significant.

## 3. Results

### 3.1. Study characteristics

**Figure 1:**
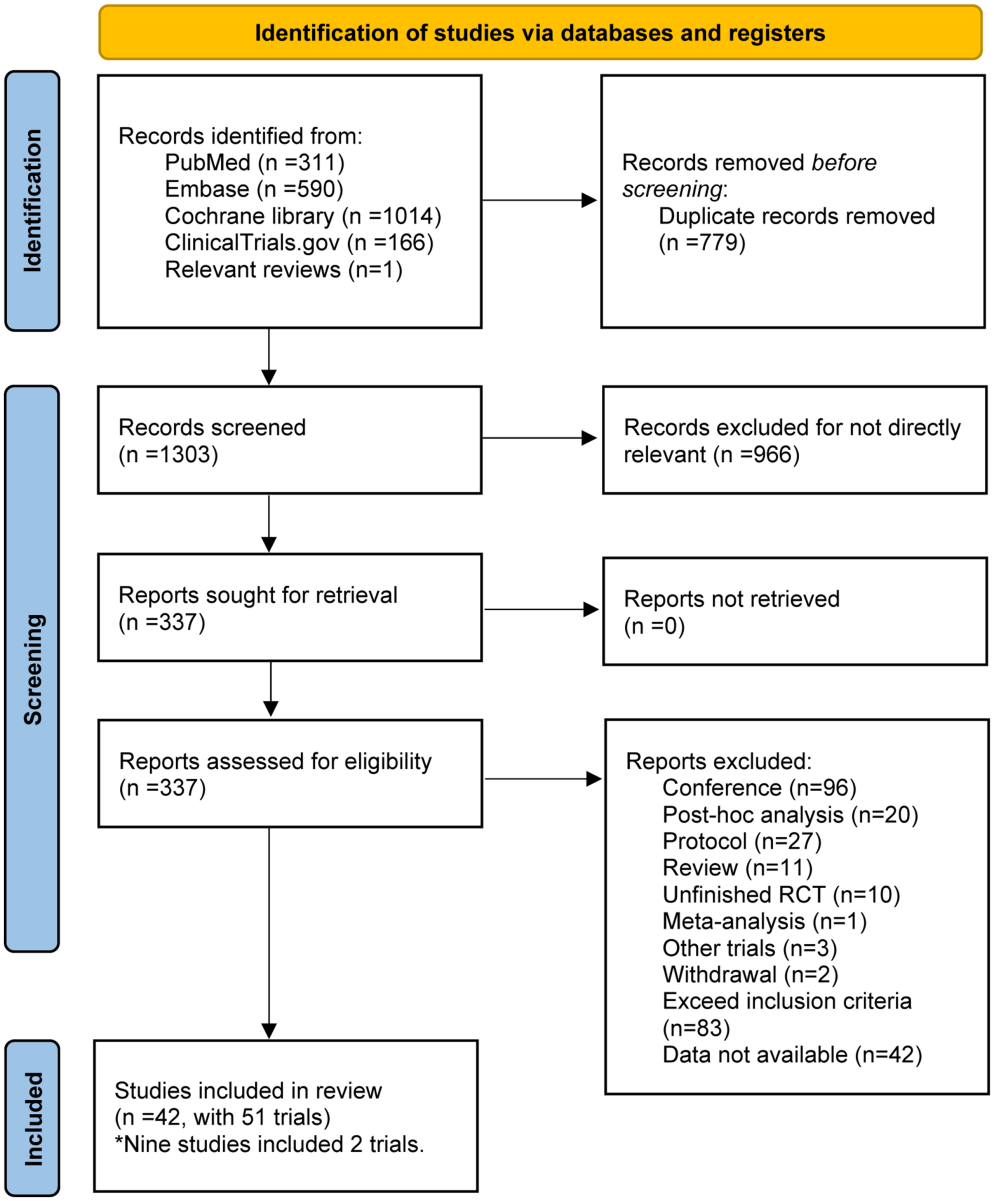
The study search, selection, and inclusion process.

PubMed, EMBASE, the Cochrane Library, and Clinicaltrials.gov together provided 2081 titles and abstracts. In addition, references from relevant studies were manually scanned, and an additional RCT was discovered. A total of 1745 articles were excluded due to duplication and irrelevance after a quick review, and 337 full articles were assessed for eligibility. Among these, 295 articles were excluded due to the inapplicable publication types: conference abstract (*n* = 96); post-hoc analysis (*n* = 20); protocol (*n* = 27); review (*n* = 11); unfinished RCTs (*n* = 10); meta-analysis (*n* = 1); other trials (*n* = 3); withdrawal (*n* = 2); exceed inclusion criteria (*n* = 83); data not available (*n* = 42). Finally, a total of 42 studies containing 51 RCTs were included in the meta-analysis. The selection process is summarized in the flow diagram (Figure 1). The main characteristics of the included studies are summarized in Supplementary Table S2.

### 3.2. Primary efficacy outcome

**Figure 2:**
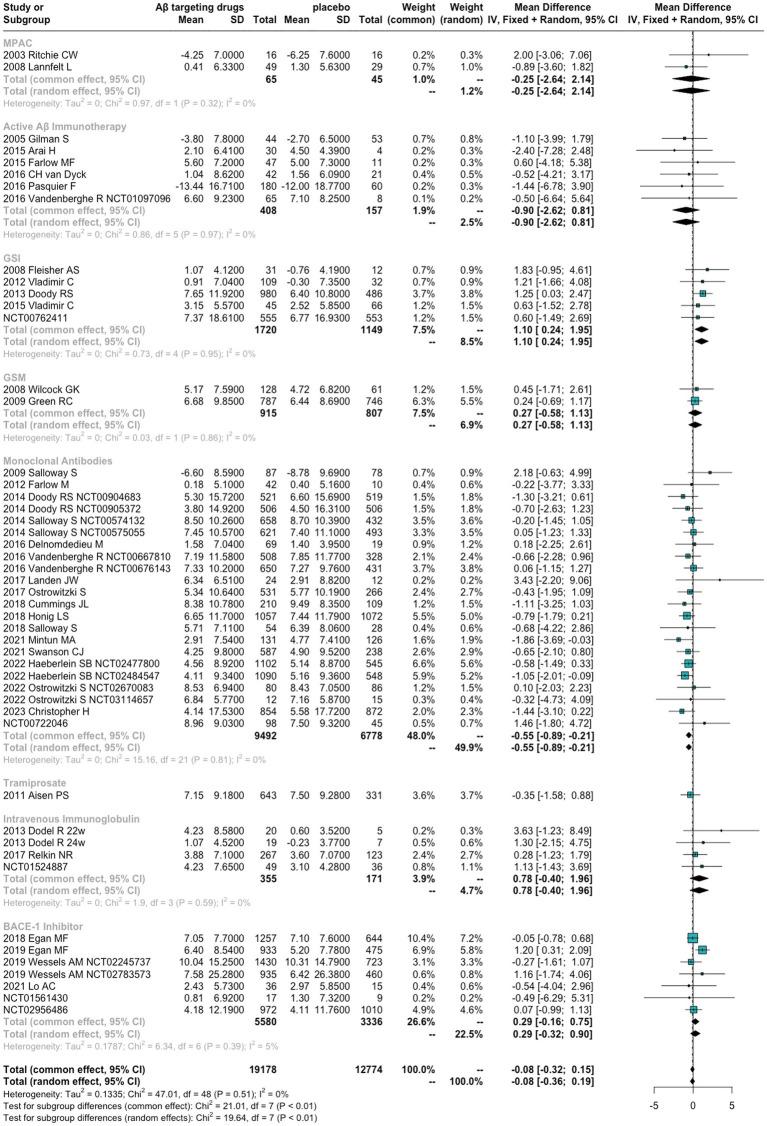
Forest plots for Alzheimer’s Disease Assessment Scale–Cognitive Subscale.

As shown in Figure 2, for ADAS-Cog, the difference between anti-Aβ agents and placebo did not meet the statistical significance [MDs = −0.08 (−0.32 to 0.15), *p* = 0.4785]. Monoclonal antibodies are the only Aβ-targeting agents more effective than placebo [MDs = −0.55 (−0.89 to 0.21), *p* = 0.001]. In contrast, GSI performed even worse than placebo in ADAS-Cog [MDs = 0.68 (0.08 to 1.29)].

### 3.3. Secondary efficacy outcome

For CDR-SB, MMSE, and ADCS-ADL, no significant differences were found between the anti-Aβ agents and the placebo. Anti-Aβ agents were even worse than placebo in NPI (MDs = 0.91 [0.42 to 1.41], *p* = 0.0003). In contrast, compared with placebo, monoclonal antibodies showed better results in CDR-SB, MMSE, and ADCS-ADL (CDR-SB: MDs = −0.19 [−0.29 to −0.10], *p* < 0.0001; MMSE: MDs = 0.19 [0.00 to 0.39], *p* = 0.05; ADCS-ADL: MDs = 1.26 [0.84 to 1.68], *p* < 0.00001). In comparison to the placebo, intravenous immunoglobulin had worse results in CDR-SB and NPI (MDs = 1.72 [0.53 to 2.90], *p* = 0.004 and MDs = 2.23 [0.14 to 4.31], *p* = 0.04, respectively). In MMSE, and ADCS-ADL, GSI was worse than placebo (MDs = −0.63 [−1.14 to −0.12]. *p* = 0.01 and MDs = −1.57 [−2.75 to −0.40], *p* = 0.007, respectively). Furthermore, BACE1 inhibitors were worse than the placebo in NPI (MDs = 2.23 [0.14 to 4.31], *p* = 0.002), and GSM was worse than the placebo in CDR-SB (MDs = 0.45 [0.17 to 0.74], p = 0.002). Table 1 shows the detailed results of the efficacy outcomes analyses. Forest plots are shown in the Supplementary Figures S1–S4.

**Table 1:** Meta-analysis of secondary efficacy outcomes and safety outcomes.

	MD (95% CI) /RR [95% CI]	*p* value
*Secondary efficacy outcomes*		
*CDR-SB*		
Tramiprosate	−0.10 (−0.54, 0.34)	0.66
GSM	**0.45 (0.17, 0.74)**	**0.002**
Intravenous immunoglobulin	**1.72 (0.53, 2.90)**	**0.004**
GSI	0.02 (−0.63, 0.68)	0.95
Active Aβ immunotherapy	0.54 (−0.44, 1.52)	0.28
BACE-1 inhibitor	0.01 (−0.14, 0.17)	0.85
Monoclonal antibodies	**−0.19 (−0.29, −0.10)**	**< 0.0001**
Overall	−0.01 (−0.14, 0.11)	0.88
*MMSE*		
MPAC	0.22 (−1.54, 1.98)	0.81
GSM	−0.38 (−0.95, 0.19)	0.19
Intravenous immunoglobulin	0.19 (−1.80, 2.19)	0.85
GSI	**−0.63 (−1.14, −0.12)**	**0.01**
Active Aβ immunotherapy	−0.18 (−1.21, 0.86)	0.74
BACE-1 inhibitor	0.09 (−0.17, 0.35)	0.49
Monoclonal antibodies	**0.19 (0.00, 0.39)**	**0.05**
Overall	0.06 (−0.09, 0.20)	0.44
*ADCS-ADL*		
GSM	−0.34 (−1.60, 0.93)	0.60
Intravenous immunoglobulin	−1.26 (−3.08, 0.55)	0.17
GSI	**−1.51 (−2.61, −0.41)**	**0.007**
Active Aβ immunotherapy	−0.60 (−5.06, 3.86)	0.79
BACE-1 inhibitor	−0.27 (−2.47, 1.94)	0.81
Monoclonal antibodies	**1.26 (0.84, 1.68)**	**< 0.00001**
Overall	−0.03 (−0.71, 0.64)	0.92
*NPI*		
GSM	0.62 (−0.61, 1.85)	0.32
Intravenous immunoglobulin	**2.23 (0.14, 4.31)**	**0.04**
GSI	0.55 (−1.63, 2.72)	0.62
Active Aβ immunotherapy	−0.94 (−4.33, 2.45)	0.59
BACE-1 inhibitor	**1.20 (0.44, 1.96)**	**0.002**
Monoclonal antibodies	0.44 (−0.65, 1.53)	0.43
Overall	**0.91 (0.42, 1.41)**	**0.0003**
*Safety outcomes*		
*SAEs*		
Tramiprosate	**1.44 [1.18, 1.76]**	**0.0003**
MPAC	0.33 [0.04, 2.87]	0.32
GSM	1.14 [0.95, 1.37]	0.16
Intravenous immunoglobulin	0.85 [0.60, 1.22]	0.38
GSI	**1.63 [1.38, 1.93]**	**< 0.00001**
Active Aβ immunotherapy	1.32 [0.90, 1.92]	0.16
BACE-1 inhibitor	**1.16 [1.05, 1.28]**	**0.003**
Monoclonal antibodies	1.05 [0.98, 1.13]	0.17
Overall	**1.15 [1.09, 1.21]**	**< 0.0001**
*Death*		
Tramiprosate	0.65 [0.33, 1.29]	0.22
MPAC	0.33 [0.01, 7.62]	0.49
GSM	1.41 [0.79, 2.54]	0.25
Intravenous immunoglobulin	0.92 [0.17, 4.97]	0.93
GSI	1.93 [0.82, 4.54]	0.13
Active Aβ immunotherapy	0.60 [0.17, 2.07]	0.42
BACE-1 inhibitor	1.09 [0.63, 1.86]	0.76
Monoclonal antibodies	0.99 [0.74, 1.31]	0.92
Overall	1.04 [0.85, 1.28]	0.72

MD, mean difference; RR, Relative Risk; CI, Confidence Interval; CDR-SB, Clinical Dementia Rating–Sum of Boxes; GSI, γ-Secretase Inhibitor; GSM, γ-Secretase Modulators; MMSE, Mini-Mental State Examination; MPAC, metal-protein–attenuating compounds; ADCS-ADL, Alzheimer’s Disease Cooperative Study–Activities of Daily Living; NPI, Neuropsychiatric Inventory; SAE, serious adverse events. Bold indicates that the result is statistically significant.

### 3.4. Safety outcome analyses

**Figure 3:**
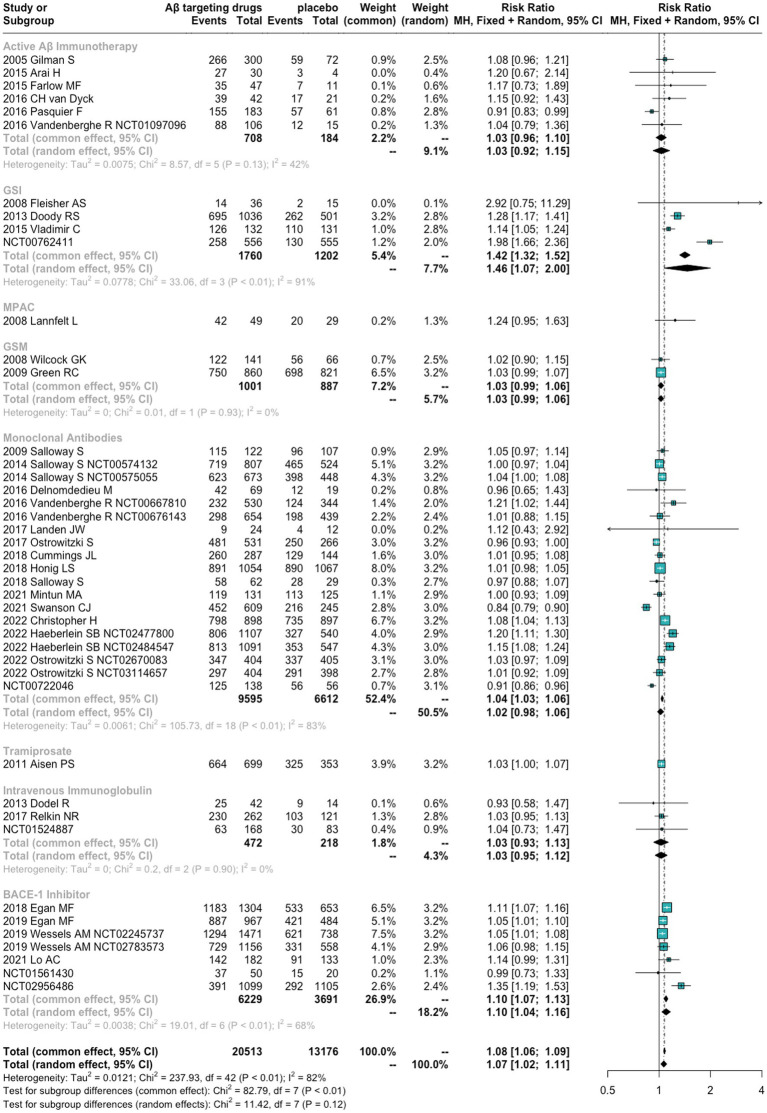
Forest plots for adverse events.

We found that anti-Aβ agents showed a significantly higher risk of AEs and SAEs than placebo [RR = 1.07 (1.02 to 1.11), *p* = 0.0014 and RR = 1.15 (1.09 to 1.21), *p* < 0.0001, respectively]. No significant differences were found between anti-Aβ agents and placebo in terms of death [RR = 1.04 (0.85, 1.28), *p* = 0.7209]. For different drug types, GSI and BACE1 Inhibitors showed a significantly high risk of AEs and SAEs. Tramiprosate was associated with SAEs. The detailed results are presented in Figure 3, Table 1, and Supplementary Figures S5, S6.

### 3.5. Subgroup analyses

To assess the influence of different follow-up times and degrees of cognitive impairment, we implemented subgroup analyses according to the characteristics at baseline. In ADAS-Cog, CDR-SB, MMSE, and ADCS-ADL, we found no difference between anti-Aβ and placebo regardless of follow-up time ≥ 72 weeks group or < 72 weeks group. For NPI, both time subgroups were worse than the placebo [< 72 weeks: MDs = 0.85 (0.36, 1.35), *p* = 0.001; ≥ 72 weeks: MDs = 4.92 (0.89, 8.95), *p* = 0.017]. In terms of safety, both time subgroups had a higher risk of AEs than the placebo [< 72 weeks: RR = 1.10 (1.02, 1.18), *p* = 0.0097; ≥ 72 weeks: RR = 1.06 (1.02, 1.11), *p* = 0.0024], but only the follow-up time ≥ 72 weeks group had a higher risk of SAEs [RR = 1.14 (1.05, 1.23), *p* = 0.0014].

For patients with mild cognitive impairment, no significant differences were found between the anti-Aβ agents and the placebo in ADAS-Cog, CDR-SB, MMSE, and ADCS-ADL, but anti-Aβ agents are inferior to the placebo in NPI [MDs = 0.95 (0.28, 1.62), *p* = 0.0056]. These results are consistent with the primary analyses, including patients with mild to moderate cognitive impairment. Monoclonal antibodies are superior to the placebo in ADAS-Cog, CDR-SB, and ADCS-ADL, while BACE1 inhibitors were inferior to the placebo in ADCS-ADL and NPI. For safety, anti-Aβ agents showed a higher risk of AEs than placebo [RR = 1.06 (1.02; 1.11), *p* = 0.0073]. No significant differences were found between anti-Aβ agents and placebo in terms of SAEs and death. The detailed results of the subgroup analyses are shown in Table 2. Forest plots are shown in the Supplementary Figures S7–S22.

**Table 2:** Subgroup analysis of efficacy and safety outcomes.

	< 72 weeks		≥ 72 weeks		Early AD	
	MD (95% CI)/RR [95% CI]	*p* value	MD (95% CI)/RR [95% CI]	*p* value	MD (95% CI)/RR [95% CI]	*p* value
*Efficacy outcomes*						
ADAS-Cog	0.43 (−0.45, 1.31)	0.343	−0.12 (−0.37, 0.12)	0.319	−0.30 (−0.61, 0.02)	0.0664
CDR-SB	0.67 (−0.11, 1.45)	0.083	−0.06 (−0.18, 0.07)	0.396	−0.14 (−0.30, 0.02)	0.0831
MMSE	−0.06 (−0.70, 0.59)	0.861	0.06 (−0.08, 0.21)	0.409	0.05 (−0.12, 0.23)	0.5468
ADCS-ADL	−1.00 (−2.71, 0.72)	0.273	0.10 (−0.62, 0.82)	0.751	0.47 (−0.43, 1.36)	0.3095
NPI	**0.85 (0.36, 1.35)**	**0.001**	**4.92 (0.89, 8.95)**	**0.017**	**0.95 (0.28, 1.62)**	**0.0056**
*Safety outcomes*						
AEs	**1.10 [1.02, 1.18]**	**0.0097**	**1.06 [1.02, 1.11]**	**0.0024**	**1.06 [1.02, 1.11]**	**0.0073**
SAEs	1.19 [0.82, 1.72]	0.3608	**1.14 [1.05, 1.23]**	**0.0014**	1.06 [0.99, 1.13]	0.1040
Death	1.10 [0.34, 3.53]	0.8688	1.04 [0.84, 1.28]	0.7390	0.79 [0.59, 1.07]	0.1289

AD, Alzheimer’s disease; MD, mean difference; RR, Relative Risk; CI, Confidence Interval; ADAS-Cog, Alzheimer’s Disease Assessment Scale–Cognitive Subscale; CDR-SB, Clinical Dementia Rating–Sum of Boxes; MMSE, Mini-Mental State Examination; ADCS-ADL, Alzheimer’s Disease Cooperative Study–Activities of Daily Living; NPI, Neuropsychiatric Inventory; AEs, adverse events; SAEs, serious adverse events. Bold indicates that the result is statistically significant.

### 3.6. Risk of bias in included studies

**Figure 4:**
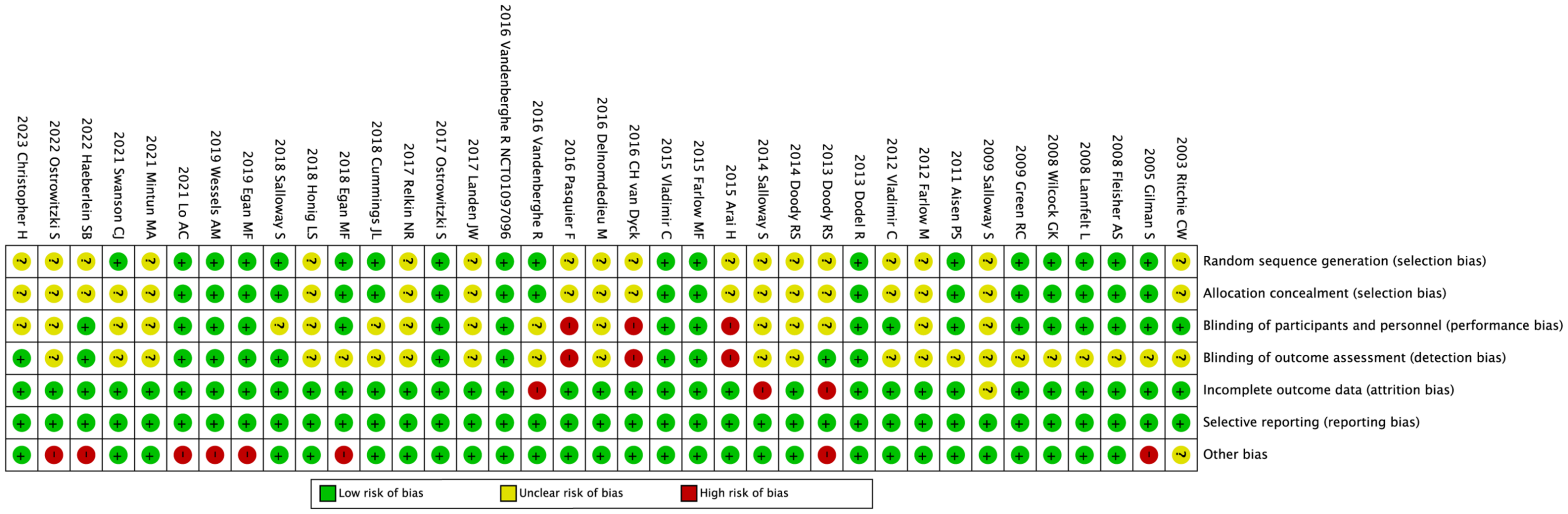
Risk of bias: a summary table for each risk of bias item for each study.

The risk of bias for 37 enrolled studies is illustrated in Figure 4; the risk of bias for the five RCTs from Clinicaltrials.gov is unclear and is not displayed in Figure 4. The risks of bias in random sequence generation and allocation concealment were unclear in eighteen clinical trials; the rest were low risk of bias. The risk of performance bias was deemed unclear in sixteen studies, high in three, and low in the rest. For blinding of outcome assessment, the risk of bias was low in twelve trials and high in three trials; the remaining risks of bias were unknown. For incomplete outcome data, unclear risks of bias were observed in one RCT, high risks of bias were observed in three RCTs, and the rest had a low risk of bias. For selective reporting, the risk of bias was low in all studies. Aside from these items, unclear risks of bias were also observed in one RCT, and risks of bias were high in eight RCTs. We also conducted sensitivity analyses which demonstrated that all the statistics were robust (Supplementary Figures S23–S30).

## 4. Discussion

**Figure 5:**
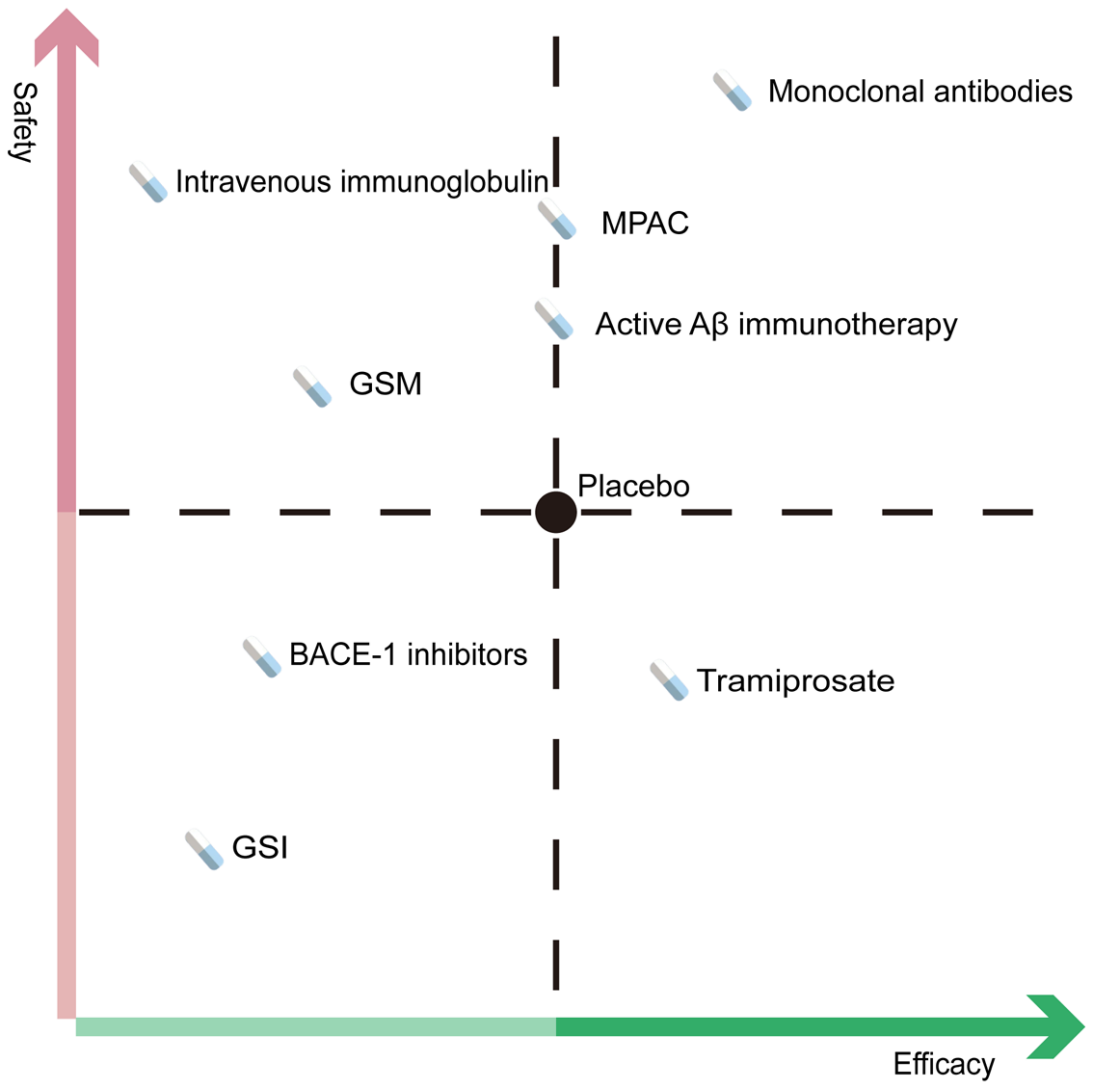
Overall conclusion of meta-analysis.

The present study included 42 studies with 33,689 individuals randomly assigned to anti-Aβ agents or placebo. Our results showed that anti-Aβ drugs are not superior to placebo in delaying cognitive deterioration in patients with AD and lead to a higher risk of AEs and SAEs. Only anti-Aβ monoclonal antibodies outperformed the placebo in delaying cognitive deterioration as measured by ADAS-Cog, CDR-SB, MMSE, and ADCS-ADL without increasing safety risks. In addition, GSI and BACE1 inhibitors exacerbated cognitive decline compared with placebo and were associated with an increased risk of AEs and SAEs. GSM and intravenous immunoglobulin increased cognitive decline in secondary efficacy outcomes but did not increase safety concerns. Although active Aβ immunotherapy was not effective in delaying cognitive decline, it was safe in patients with AD. The overall conclusion is represented in Figure 5. In both short and long-term follow-up studies, subgroup analysis revealed that there was no difference between anti-Aβ drugs and placebo in delaying cognitive decline. Long-term follow-up studies, on the other hand, linked a higher risk of SAEs. In the subgroup analysis that included only patients with early AD, anti-Aβ drugs showed better results than the analysis that included mild to moderate AD patients in delaying cognitive deterioration and without increasing the risk of SAEs.

Our results showed that two secretase inhibitors had a negative effect on cognitive function in AD patients. BACE1 inhibitors have been found to show superior effects in mice but not in human trials, and some BACE1 inhibitors have safety concerns (Moussa-Pacha et al., 2020). This is similar to our results. Our subgroup analysis found an interesting result that in AD patients with only mild cognitive impairment, BACE1 inhibitors were associated with an exacerbation of cognitive decline in even more secondary efficacy outcomes and safety concerns remain. However, because only one RCT was included in this subgroup analysis, this result needs to be interpreted with caution. Our results indicated that GSI exacerbated cognitive deterioration. This is also consistent with the findings of a previous study in which acute administration of GSI improved cognitive deficits in mice while chronic administration impaired normal cognition (Mitani et al., 2012). GSI raises significant safety concerns, possibly because γ-secretase also cleaves Notch protein, a protein that regulates cell proliferation, differentiation and growth (Bray, 2016). Notch signaling inhibition may result in gastrointestinal disorders, thymic atrophy, lymphocytopenia, and hair color changes (Panza et al., 2010). Furthermore, GSI inevitably increases the β-C terminal fragment of the APP, which may have adverse synaptic effects (Hur, 2022). GSM regulates γ-secretase activity rather than inhibiting the entire γ-secretase activity and does not cause APP-CTF accumulation or Notch inhibition, and thus has a better safety profile compared to GSI (Crump et al., 2013). This is also consistent with our conclusion that GSM does not increase the risk of adverse events. Nonetheless, GSM did not outperform the placebo in terms of delayed cognitive decline. It has been suggested that the combination of GSI and GSM may be synergistic and may be an attractive strategy for AD (Yang et al., 2021). However, according to the present meta-analysis, it should be carefully considered whether BACE1 and γ-secretase should be investigated further as potential targets for the treatment of AD.

Our results are consistent with some previous studies that active Aβ immunotherapy, MPAC, or tramiprosate did not show efficacy for AD (Lannfelt et al., 2014; Yadollahikhales and Rojas, 2023). Although current active Aβ immunotherapy has not shown satisfactory clinical results, some DNA-based vaccines are currently in clinical trials and may show satisfactory clinical results in the future. We included only one RCT on tramiprosate, and it is worth mentioning that although our results showed that tramiprosate had a poor safety profile, the results of the RCT showed that the safety profile of tramiprosate was dose-related, and the incidence of AEs with 100 mg of tramiprosate did not differ from that of placebo. Therefore, future clinical trials about tramiprosate should focus on the effect of dose.

Successive failures have brought the Aβ hypothesis into considerable question (Kametani and Hasegawa, 2018). Skeptics of the ACH argue that AD progression may be caused by complicating factors and the Aβ hypothesis is insufficient to explain disease progression (Egan et al., 2018). Furthermore, the accumulation of Aβ plaques in AD may be a response to certain upstream events and represent a collateral phenomenon (Panza et al., 2019). Clearance of Aβ thus did not affect clinical cognitive function (Alexander et al., 2021). However, some argue that these failures do not disprove ACH. There are several explanations for the persistently negative clinical trial results: the accumulation of Aβ occurs in the first few years of dementia symptoms in AD patients, and reducing Aβ production after the patient develops cognitive impairment provides no clinical benefit (Sperling et al., 2014). This is consistent with our findings that anti-Aβ drugs showed better results in patients with early AD, although still not clinically effective. Another possibility is that some clinical trials have included people without evidence of brain Aβ pathology, which may have contributed to the failures (Abbott and Dolgin, 2016; Panza et al., 2019).

The FDA approved aducanumab on June 7, 2021, based on the substitute endpoint that aducanumab reduces Aβ (Knopman and Perlmutter, 2021). However, to date, there is no clinical evidence that a reduction in Aβ results in cognitive improvement (Alexander et al., 2021). As a result, the approval of aducanumab has also generated massive controversy (Knopman and Perlmutter, 2021). Lecanemab received its first approval in the United States on January 6, 2023, following the results of a large RCT showing that lecanemab was effective in delaying cognitive decline (Hoy, 2023; Larkin, 2023). It is the second approved drug targeting Aβ as well as the second approved monoclonal antibody for AD. The clinical results of lecanemab are also regarded as a historic moment of disease-modifying therapies for AD (Mead and Fox, 2023). A previous meta-analysis of monoclonal antibodies for AD found that patients receiving monoclonal antibodies demonstrated lower clinical deterioration for the CDR-SB score (Lacorte et al., 2022). This is consistent with our findings that anti-Aβ monoclonal antibodies are the only effective targeting Aβ drugs currently available for AD. The positive results of anti-Aβ monoclonal antibodies, on the other hand, show that Aβ is still a valuable therapeutic target. The reason for the failures of the previous trials may be because Aβ plaque needs to be reduced to a low enough level to show a corresponding clinical benefit (Karran and De Strooper, 2022).

Lu et al. suggest that anti-Aβ drugs are unlikely to have an effect on slowing cognition with anti-Aβ interventions in patients with AD, however, the drug classes that increase Aβ clearance may be effective, possibly due to the inclusion of anti-Aβ monoclonal antibodies in drugs that increase Aβ clearance (Lu et al., 2020). A meta-analysis of intravenous immunoglobulin for AD indicated that intravenous immunoglobulin did not show effectiveness in slowing cognitive decline in AD patients but with good safety (Liu and Wang, 2019). This is generally consistent with our results, except that we additionally found that intravenous immunoglobulin even exacerbated cognitive deterioration in CDR-SB, an outcome they did not use as an outcome indicator. Aβ-targeted therapy for AD is complex, but the results from our subgroup analysis suggest that the future prospects of anti-Aβ monoclonal antibody therapy for AD are promising. In addition to determining the optimal treatment strategy, future research still needs to focus on the optimal timing of intervention in AD, and from our findings, early intervention may lead to better outcomes. Overall, pharmacologic interventions for AD are still evolving and future prospects depend on ongoing research and clinical trials. Current research continues to explore the efficacy and safety of various approaches to treating AD, including Aβ-targeted therapies, tau-targeted therapies, lifestyle interventions and combination therapies. Pathologic changes associated with tau are considered as the pathological events downstream caused by the accumulation of Aβ (He et al., 2018). However, studies of tau have indicated that tau pathology can progress independently of Aβ accumulation (van der Kant et al., 2020). Several preclinical studies have shown that lowering the levels of soluble tau reverses neurodegeneration and memory loss in mice even at advanced stages of the disease (DeVos et al., 2017; Busche et al., 2019). Tau-targeted therapies could therefore also be a potential strategy for treating AD as an alternative or complementary therapy to Aβ-targeted therapies.

Inevitably, there were several limitations of the present meta-analysis. First, although a comprehensive literature search was conducted to include 42 studies, there were differences between the number of RCTs for each class of drugs. GSM and MPAC included only two RCTs respectively, and tramiprosate included only one RCT, thus the analysis of these drugs had limited credibility. Besides, we combined data from experimental groups with different doses into one group, which may reduce the credibility of the results because we did not take into account the discrepancies caused by different doses, and it is clear that higher drug doses are associated with better clinical outcomes but lower safety. Although we performed subgroup analyses based on the degree of cognitive impairment of the included patients and the follow-up time, differences in study design, inclusion and exclusion criteria, and baseline characteristics (e.g., gender, study area, ethnicity) may also have contributed to differences. The AE results revealed a high degree of heterogeneity. Our sensitivity analysis, however, revealed that removing either RCT had no effect on the AE results. Furthermore, AE demonstrated high heterogeneity in the subgroup analysis. Thus, we did not find significant influences on heterogeneity, which is one of the limitations of our study.

## 5. Conclusion

In conclusion, current evidence indicates that anti-Aβ drugs do not delay cognitive decline in patients with AD. Intervention early in AD may lead to better outcomes, but not clinically significant. Anti-Aβ monoclonal antibodies effectively slow cognitive deterioration as measured by ADAS-Cog, CDR-SB, and ADCS-ADL, offering new hope for developing targeted Aβ drugs. BACE1 inhibitors and GSI exacerbate cognitive deterioration and cause safety concerns. Intravenous immunoglobulin and GSM increased cognitive decline but have acceptable safety profiles. No evidence indicates active Aβ immunotherapy, MPAC, or tramiprosate have effects on cognitive function and tramiprosate is associated with serious adverse events.

## Data availability statement

The original contributions presented in the study are included in the article/Supplementary material, further inquiries can be directed to the corresponding authors.

## Author contributions

JL: Writing – original draft, Writing – review & editing. XinW: Writing – original draft, Writing – review & editing. XT: Formal analysis, Writing – original draft. SW: Writing – original draft. RQ: Writing – original draft. XiaW: Writing – original draft. ZC: Project administration, Writing – review & editing. ZW: Project administration, Writing – review & editing. GC: Writing – review & editing, Project administration.

## Glossary

AD: Alzheimer’s disease
Aβ: amyloid-β
ACH: Aβ cascade hypothesis
FDA: Food and Drug Administration
APP: Aβ precursor protein
BACE1: β-site APP cleaving enzyme-1
GSI: γ-secretase inhibitors
GSM: γ-secretase modulators
RCT: randomized controlled trial
MPAC: metal-protein–attenuating compounds
ADAS-Cog: Alzheimer’s Disease Assessment Scale–Cognitive Subscale
CDR-SB: Clinical Dementia Rating–Sum of Boxes
MMSE: Mini-Mental State Examination
ADCS-ADL: Alzheimer’s Disease Cooperative Study–Activities of Daily Living
NPI: Neuropsychiatric Inventory
AE: adverse event
SAE: serious adverse event
MD: mean difference
CI: confidence interval
RR: risk ratio
SD: standard deviation
