# Supplementary material for: The efficacy and safety of anti-Aβ agents for delaying cognitive decline in Alzheimer’s disease: a meta-analysis
Source: Front Aging Neurosci. 2023 Nov 6;15:1257973. doi: 10.3389/fnagi.2023.1257973 (PMC10661413; doi:10.3389/fnagi.2023.1257973)

Journal name: Frontiers in Aging Neuroscience

The efficacy and safety of anti-Aβ agents for delaying cognitive decline in Alzheimer's disease: A meta-analysis

**Jiaxuan Li ^1,^** ^†^**, Xin Wu ^1^** ^†^**, Xin Tan ^2^, Shixin Wang ^1^, Ruisi Qu ^1^, Xiaofeng Wu ^3^, Zhouqing Chen ^1, *^, Zhong Wang** **^1, *^, Gang Chen ^1, *^**

^1^ Department of Neurosurgery & Brain and Nerve Research Laboratory, The First Affiliated Hospital of Soochow University, Suzhou, Jiangsu Province, China

^2^ Department of Neurology, The Affiliated Suzhou Hospital of Nanjing Medical University, Suzhou

Municipal Hospital, Suzhou, Jiangsu Province, China

^3^ Department of Ultrasound, the First Affiliated Hospital of Soochow University, Suzhou, Jiangsu Province, China

^†^ Jiaxuan Li and Xin Wu contributed equally to this work.

**^*^ Correspondence:**

Gang Chen, Department of Neurosurgery, The First Affiliated Hospital of Soochow University, 188 Shizi Street, Suzhou 215006, China. Email address: nju_neurosurgery@163.com.

Zhong Wang, Department of Neurosurgery, The First Affiliated Hospital of Soochow University, 188 Shizi Street, Suzhou 215006, China. Email address: wangz8761@163.com.

Zhouqing Chen, Department of Neurosurgery, The First Affiliated Hospital of Soochow University, 188 Shizi Street, Suzhou 215006, China. Email address: zqchen6@163.com

**Figure legends**

Table S1: The detailed search strategy.

Table S2: Characteristics of the included studies.

Table S3: Inclusion, Exclusion criteria, Study design and Outcome assessments of the Included Studies.

Table S4: Quality assessment of the included studies.

Figure S1: Forest plot of meta-analysis: CDR-SB

Figure S2: Forest plot of meta-analysis: MMSE

Figure S3: Forest plot of meta-analysis: ADCS-ADL

Figure S4: Forest plot of meta-analysis: NPI

Figure S5: Forest plot of meta-analysis: SAEs

Figure S6: Forest plot of meta-analysis: Death

Figure S7: Forest plot of sub-analysis based on different follow-up times: ADAS-Cog

Figure S8: Forest plot of sub-analysis based on different follow-up times: CDR-SB

Figure S9: Forest plot of sub-analysis based on different follow-up times: MMSE

Figure S10: Forest plot of sub-analysis based on different follow-up times: ADCS-ADL

Figure S11: Forest plot of sub-analysis based on different follow-up times: NPI

Figure S12: Forest plot of sub-analysis based on different follow-up times: AEs

Figure S13: Forest plot of sub-analysis based on different follow-up times: SAEs

Figure S14: Forest plot of sub-analysis based on different follow-up times: Death

Figure S15: Forest plot of ADAS-Cog in patients with mild cognitive impairment

Figure S16: Forest plot of CDR-SB in patients with mild cognitive impairment

Figure S17: Forest plot of MMSE in patients with mild cognitive impairment

Figure S18: Forest plot of ADCS-ADL in patients with mild cognitive impairment

Figure S19: Forest plot of NPI in patients with mild cognitive impairment

Figure S20: Forest plot of AEs in patients with mild cognitive impairment

Figure S21: Forest plot of SAEs in patients with mild cognitive impairment

Figure S22: Forest plot of Death in patients with mild cognitive impairment

Figure S23: Forest plot of sensitive analysis: ADAS-Cog

Figure S24: Forest plot of sensitive analysis: CDR-SB

Figure S25: Forest plot of sensitive analysis: MMSE

Figure S26: Forest plot of sensitive analysis: ADCS-ADL

Figure S27: Forest plot of sensitive analysis: NPI

Figure S28: Forest plot of sensitive analysis: AEs

Figure S29: Forest plot of sensitive analysis: SAEs

Figure S30: Forest plot of sensitive analysis: Death

Figure S31: Funnel plot of sensitive analysis: ADAS-Cog

Figure S32: Funnel plot of sensitive analysis: CDR-SB

Figure S33: Funnel plot of sensitive analysis: MMSE

Figure S34: Funnel plot of sensitive analysis: ADCS-ADL

Figure S35: Funnel plot of sensitive analysis: NPI

Figure S36: Funnel plot of sensitive analysis: AEs

Figure S37: Funnel plot of sensitive analysis: SAEs

Figure S38: Funnel plot of sensitive analysis: Death

**Table S1: Detailed Search Strategy**

**Search date: January 18, 2023**

**Pubmed：**

| Search | Query | Results |
| --- | --- | --- |
| #1 | "Randomized controlled trial"[Filter] | 585,985 |
| #2 | "Alzheimer Disease"[Mesh] | 114,553 |
| #3 | (((((((((((((((((((((((((((((((((( (Alzheimer Dementia[Title/Abstract])) OR (Alzheimer Dementias[Title/Abstract])) OR (Dementia, Alzheimer[Title/Abstract])) OR (Alzheimer's Disease[Title/Abstract])) OR (Dementia, Senile[Title/Abstract])) OR (Senile Dementia[Title/Abstract])) OR (Dementia, Alzheimer Type[Title/Abstract])) OR (Alzheimer Type Dementia[Title/Abstract])) OR (Alzheimer-Type Dementia (ATD[Title/Abstract]))) OR (Alzheimer Type Dementia (ATD[Title/Abstract]))) OR (Dementia, Alzheimer-Type (ATD[Title/Abstract]))) OR (Alzheimer Type Senile Dementia[Title/Abstract])) OR (Primary Senile Degenerative Dementia[Title/Abstract])) OR (Dementia, Primary Senile Degenerative[Title/Abstract])) OR (Alzheimer Sclerosis[Title/Abstract])) OR (Sclerosis, Alzheimer[Title/Abstract])) OR (Alzheimer Syndrome[Title/Abstract])) OR (Alzheimer's Diseases[Title/Abstract])) OR (Alzheimer Diseases[Title/Abstract])) OR (Alzheimers Diseases[Title/Abstract])) OR (Senile Dementia, Alzheimer Type[Title/Abstract])) OR (Acute Confusional Senile Dementia[Title/Abstract])) OR (Senile Dementia, Acute Confusional[Title/Abstract])) OR (Dementia, Presenile[Title/Abstract])) OR (Presenile Dementia[Title/Abstract])) OR (Alzheimer Disease, Late Onset[Title/Abstract])) OR (Late Onset Alzheimer Disease[Title/Abstract])) OR (Alzheimer's Disease, Focal Onset[Title/Abstract])) OR (Focal Onset Alzheimer's Disease[Title/Abstract])) OR (Familial Alzheimer Disease (FAD[Title/Abstract]))) OR (Alzheimer Disease, Familial (FAD[Title/Abstract]))) OR (Familial Alzheimer Diseases (FAD[Title/Abstract]))) OR (Alzheimer Disease, Early Onset[Title/Abstract])) OR (Early Onset Alzheimer Disease[Title/Abstract])) OR (Presenile Alzheimer Dementia[Title/Abstract]) | 153,688 |
| #4 | #2 OR #3 | 180,384 |
| #5 | "Amyloid beta-Peptides"[Mesh] | 38,127 |
| #6 | (((((((((((((((((((((((((((((Amyloid beta Peptides[Title/Abstract]) OR (beta-Peptides, Amyloid[Title/Abstract])) OR (Alzheimer beta-Protein[Title/Abstract])) OR (Alzheimer beta Protein[Title/Abstract])) OR (beta-Protein, Alzheimer[Title/Abstract])) OR (Alzheimer's ABP[Title/Abstract])) OR (ABP, Alzheimer's[Title/Abstract])) OR (Alzheimer ABP[Title/Abstract])) OR (Alzheimers ABP[Title/Abstract])) OR (Alzheimer's Amyloid Fibril Protein[Title/Abstract])) OR (beta-Amyloid Protein[Title/Abstract])) OR (Protein, beta-Amyloid[Title/Abstract])) OR (beta Amyloid Protein[Title/Abstract])) OR (Amyloid beta-Peptide[Title/Abstract])) OR (Amyloid beta Peptide[Title/Abstract])) OR (beta-Peptide, Amyloid[Title/Abstract])) OR (Amyloid beta-Protein[Title/Abstract])) OR (Amyloid beta Protein[Title/Abstract])) OR (beta-Protein, Amyloid[Title/Abstract])) OR (Amyloid beta-Proteins[Title/Abstract])) OR (Amyloid beta Proteins[Title/Abstract])) OR (beta-Proteins, Amyloid[Title/Abstract])) OR (Amyloid Fibril Protein, Alzheimer's[Title/Abstract])) OR (Amyloid Protein A4[Title/Abstract])) OR (Protein A4, Amyloid[Title/Abstract])) OR (beta Amyloid[Title/Abstract])) OR (Amyloid, beta[Title/Abstract])) OR (Amyloid AD-AP[Title/Abstract])) OR (AD-AP, Amyloid[Title/Abstract])) OR (Amyloid AD AP[Title/Abstract]) | 64,270 |
| #7 | #5 OR #6 | 67,236 |
| #8 | #1 AND #4 AND #7 | 288 |
| #9 | "Immunoglobulins, Intravenous"[Mesh] | 15,268 |
| #10 | ((((((((((((((((((((((((((((((((((((((((((Antibodies, Intravenous[Title/Abstract]) OR (Intravenous Antibodies[Title/Abstract])) OR (Immune Globulin, Intravenous[Title/Abstract])) OR (Intravenous Immune Globulin[Title/Abstract])) OR (Intravenous Immunoglobulins[Title/Abstract])) OR (Intravenous IG[Title/Abstract])) OR (IV Immunoglobulins[Title/Abstract])) OR (Immunoglobulins, IV[Title/Abstract])) OR (IVIG[Title/Abstract])) OR (IV Immunoglobulin[Title/Abstract])) OR (Immunoglobulin, IV[Title/Abstract])) OR (Intravenous Immunoglobulin[Title/Abstract])) OR (Immunoglobulin, Intravenous[Title/Abstract])) OR (Flebogamma DIF[Title/Abstract])) OR (Gamunex[Title/Abstract])) OR (Globulin-N[Title/Abstract])) OR (Globulin N[Title/Abstract])) OR (Intraglobin[Title/Abstract])) OR (Intraglobin F[Title/Abstract])) OR (Intravenous Immunoglobulins, Human[Title/Abstract])) OR (Human Intravenous Immunoglobulins[Title/Abstract])) OR (Immunoglobulins, Human Intravenous[Title/Abstract])) OR (Immune Globulin Intravenous (Human[Title/Abstract]))) OR (Immunoglobulins, Intravenous, Human[Title/Abstract])) OR (Human Intravenous Immunoglobulin[Title/Abstract])) OR (Immunoglobulin, Human Intravenous[Title/Abstract])) OR (Intravenous Immunoglobulin, Human[Title/Abstract])) OR (Gammagard[Title/Abstract])) OR (Gamimune[Title/Abstract])) OR (Gamimmune[Title/Abstract])) OR (Modified Immune Globulin (Anti-Echovirus Antibody[Title/Abstract]))) OR (Privigen[Title/Abstract])) OR (Sandoglobulin[Title/Abstract])) OR (Venoglobulin[Title/Abstract])) OR (Venoglobulin-I[Title/Abstract])) OR (Venoglobulin I[Title/Abstract])) OR (Venimmune[Title/Abstract])) OR (Iveegam[Title/Abstract])) OR (Alphaglobin[Title/Abstract])) OR (Endobulin[Title/Abstract])) OR (Gamimune N[Title/Abstract])) OR (Gamimmune N[Title/Abstract])) OR (Gammonativ[Title/Abstract]) | 23,165 |
| #11 | 7 OR #9 OR #10 | 95,154 |
| #12 | ((((((((((((((((((((((((((((((((((((((((((((((((((((((((((((((((((((((((((((((((((((((((((((((((((((((((((((R)-4-cyclopropyl-7,8-difluoro-5-(4-(trifluoromethyl)phenylsulfonyl)-4,5-dihydro-1H-pyrazolo(4,3-c)quinoline[Title/Abstract]) OR ((1-(4-methyl-1-(6-(trifluoromethyl)-3-pyridinyl)pentyl)-2-(4-(trifluoromethyl)phenyl)-4-piperidinyl)acetic acid[Title/Abstract])) OR ((S,E)-1-(2-methoxy-4-((4-(3,4,5-trifluorophenyl)-3,4,7,8-tetrahydropyrido(2,1-c)(1,2,4)oxadiazin-9(6H)-ylidene)methyl)phenyl)-4-methyl-1H-imidazol-3-ium-hemi-(E)-3-carboxyacrylate[Title/Abstract])) OR (1-((1,1,1-trifluoro-7-methyloctan-4-yl)-2-(4-(trifluoromethyl)phenyl)piperidin-4-yl)acetic acid[Title/Abstract])) OR (1-(1-(4-fluorophenyl)ethyl)-3-((3-methoxy-4-(4-methyl-1H-imidazol-1-yl)phenyl)methylene)-2-piperidinone[Title/Abstract])) OR (1-(3',4'-dichloro-2-fluoro(1,1'-biphenyl)-4-yl)cyclopropanecarboxylic acid[Title/Abstract])) OR (2',3',4',5,5',6,7,8,9,10-decahydro-2-(5-(4-fluorophenyl)-1-methylpyrazol-3-yl)-5'-(2,2,2-trifluoroethyl)spiro(6,9-methanobenzocycloocten-11,3'-(1,2,5)thiadiazole) 1',1'-dioxide[Title/Abstract])) OR (2,2-dimethyl-N-(6-oxo-6,7-dihydro-5H-dibenzo(b,d)azepin-7-yl)-N'-(2,2,3,3,3-pentafluoropropyl)malonamide[Title/Abstract])) OR (2-(((3,5-difluorophenyl)acetyl)amino)-N-(1-methyl-2-oxo-5-phenyl-2,3-dihydro-1H-1,4-benzodiazepin-3-yl)propanamide[Title/Abstract])) OR (2-(2-(3,5-difluorophenyl)-2-hydroxyacetamido)-N-(3-methyl-4-oxo-4,5-dihydro-3H-benzo(d)(1,2)diazepin-5-yl)propanamide[Title/Abstract])) OR (2-(2-(3,5-difluorophenyl)-acetylamino)-N-(1-methyl-2-oxo-5-phenyl-2,3-dihydro-1H-benzo(e)(1,4)diazepin-3-yl)propionamide[Title/Abstract])) OR (2-(3,5-bis(4-(trifluoromethyl)phenyl)phenyl)-4-methylpentanoic acid[Title/Abstract])) OR (2-(3-(3-methoxy-4-(2-methyl-1,3-oxazol-5-yl)phenyl) -8-(3,4,5-trifluorophenoxy)-5,6,7,8-tetrahydro(1,2,4)triazolo(4,3-a)pyridin-8-yl)propan-2-ol[Title/Abstract])) OR (2-(5-chloro-6-(2,2,2-trifluoroethoxy)-4'-(trifluoromethyl)biphenyl-3-yl)-3-cyclobutylpropanoic acid[Title/Abstract])) OR (2-(N-((3-(1,2,4-oxadiazol-3-yl)bicyclo(1.1.1)pentan-1-yl)methyl)-4-chlorophenylsulfonamido)-5,5,5-trifluoropentanamide[Title/Abstract])) OR (24-diamino-5-phenylthiazole[Title/Abstract])) OR (DAPT cpd[Title/Abstract])) OR (3-((cyclohexylmethyl)(3,3-dimethylbutyl)amino)-2-(4-(trifluoromethyl)phenyl)cyclohexyl)acetic acid[Title/Abstract])) OR (3-(3,4-difluorophenyl)-2-(4-fluorophenyl)-4-hydroxy-N-(1-methyl-2-oxo-5-phenyl-2,3-dihydro-1H-benzo(e)(1,4)diazepin-3-yl)butyramide[Title/Abstract])) OR (3-(4-((4-chlorophenyl)sulfonyl)-4-(2,5-difluorophenyl)cyclohexyl)propanoic acid[Title/Abstract])) OR (4-cyclopropyl-8-fluoro-5-(6-(trifluoromethyl)pyridin-3-ylsulfonyl)-4,5-dihydro-2H-pyrazolo(4,3-c)quinoline[Title/Abstract])) OR (7-(4-fluorophenyl)-N2-(3-methoxy-4-(3-methyl-1H-1,2,4-triazol-1-yl)phenyl)-N4-methyl-6,7-dihydro-5H-cyclopenta(d)pyrimidine-2,4-diamine[Title/Abstract])) OR (AZ1136[Title/Abstract])) OR (AZ3303[Title/Abstract])) OR (AZ4800[Title/Abstract])) OR (begacestat[Title/Abstract])) OR (benzyloxycarbonyl-isoleucyl-leucinal[Title/Abstract])) OR (Z-IL-CHO[Title/Abstract])) OR (Z-Ile-Leu-CHO[Title/Abstract])) OR (GSI-XII cpd[Title/Abstract])) OR (benzyloxycarbonyl-leucyl-leucyl-norleucinal[Title/Abstract])) OR (GSI I cpd[Title/Abstract])) OR (Z-Leu-Leu-Nle-CHO[Title/Abstract])) OR (Z-LLNle-CHO[Title/Abstract])) OR (GSI compound[Title/Abstract])) OR (GSI-I cpd[Title/Abstract])) OR (BIIB042[Title/Abstract])) OR (BMS 708163[Title/Abstract])) OR (BMS708163[Title/Abstract])) OR (BMS-708163[Title/Abstract])) OR (avagacestat[Title/Abstract])) OR (BMS-986115[Title/Abstract])) OR ((2R,3S)-N-((3S)-5-(3-fluorophenyl)-9-methyl-2-oxo-2,3-dihydro-1H-1,4-benzodiazepin-3-yl)-2,3-bis(3,3,3-trifluoropropyl)succinamide[Title/Abstract])) OR (butanediamide, N1-((3S)-5-(3-fluorophenyl)-2,3-dihydro-9-methyl-2-oxo-1H-1,4-benzodiazepin-3-yl)-2,3-bis(3,3,3-trifluoropropyl)-, (2R,3S)-[Title/Abstract])) OR (BPN-15606[Title/Abstract])) OR (N-(1-(4-fluorophenyl)ethyl)-6-(6-methoxy-5-(4-methyl-1H-imidazol-1-yl)pyridin-2-yl)-4-methylpyridazin-3-amine[Title/Abstract])) OR (curcumin-phenylalanine[Title/Abstract])) OR (curcumin-valine[Title/Abstract])) OR (cyclohexyl 2,7,7-trimethyl-4-(4-nitrophenyl)-5-oxo-1,4,5,6,7,8-hexahydroquinoline-3-carboxylate[Title/Abstract])) OR (dibenzazepine[Title/Abstract])) OR (iminostilbene[Title/Abstract])) OR (GSI XX[Title/Abstract])) OR (iminostilbene, 10-(13)C-labeled[Title/Abstract])) OR (ELN594[Title/Abstract])) OR (FRM-36143[Title/Abstract])) OR (L 685458[Title/Abstract])) OR (GSI-X cpd[Title/Abstract])) OR (L-685,458[Title/Abstract])) OR (L-685458[Title/Abstract])) OR (1-benzyl-4-(1-(1-carbamoyl-2-phenylethylcarbamoyl-3-methylbutylcarbamoyl)-2-hydroxy-5-phenylpentyl)carbamic acid tert-butyl ester[Title/Abstract])) OR (L 685,458[Title/Abstract])) OR (LY900009[Title/Abstract])) OR (MRK 003[Title/Abstract])) OR (MRK003[Title/Abstract])) OR (MRK-003[Title/Abstract])) OR (MRK 560[Title/Abstract])) OR (MRK560[Title/Abstract])) OR (MRK-560[Title/Abstract])) OR (N-(4-((4-chlorophenyl)sulfonyl)-4-(2,5-difluorophenyl)cyclohexyl)-1,1,1-trifluoromethanesulfonamide[Title/Abstract])) OR (MW167[Title/Abstract])) OR (MW167 cpd[Title/Abstract])) OR (N-(N-(3,5-difluorophenacetyl)alanyl)phenylglycine tert-butyl ester[Title/Abstract])) OR (DAPT butylester[Title/Abstract])) OR (N-(N-(3,5-difluorophenylacetyl)alanyl)phenyloglycine t-butyl ester[Title/Abstract])) OR (N-(N-(3,5-difluorophenacetyl)alanyl)phenylglycine t-butyl ester[Title/Abstract])) OR (N-(2FPhAc)Ala-phenyl-Gly t-butyl ester[Title/Abstract])) OR (GSI-IX cpd[Title/Abstract])) OR (N-(N-(3,5-difluorophenacetyl)alanyl)-S-phenylglycine t-butyl ester[Title/Abstract])) OR (DAPT peptide[Title/Abstract])) OR (N-(N-(3,5-difluorophenylacetyl)alanyl)phenylglycine 4-benzoylbenzyl amide[Title/Abstract])) OR (NN-DAPBB amide[Title/Abstract])) OR (N2-((2S)-2-(3,5-difluorophenyl)-2-hydroxyethanoyl)-N1-((7S)-5-methyl-6-oxo-6,7-dihydro-5H-dibenzo(b,d)azepin-7-yl)-L-alaninamide[Title/Abstract])) OR (nirogacestat[Title/Abstract])) OR (PF 03084014[Title/Abstract])) OR (PF03084014[Title/Abstract])) OR (PF-03084014[Title/Abstract])) OR (2-(5,7-difluoro-1,2,3,4-tetrahydronaphthalen-3-ylamino)-N-(1-(2-methyl-1-(neopentylamino)propan-2-yl)-1H-imidazol-4-yl)pentanamide[Title/Abstract])) OR (SCH 1500022[Title/Abstract])) OR (SCH1500022[Title/Abstract])) OR (SCH-1500022[Title/Abstract])) OR (SCH 900229[Title/Abstract])) OR (SPI-1865[Title/Abstract])) OR (tarenflurbil[Title/Abstract])) OR (MPC-7869[Title/Abstract])) OR (MPC7869[Title/Abstract])) OR (flurizan[Title/Abstract])) OR (TMP-001[Title/Abstract])) OR (TMP001[Title/Abstract])) OR (Semagacestat[Title/Abstract])) OR (N2-(2-(3,5-difluorophenyl)-2-hydroxyethanoyl)-N1-(5-methyl-6-oxo-6,7-dihydro-5H-dibenzo(b,d)azepin-7-yl)alaninamide[Title/Abstract])) OR (LY 411575[Title/Abstract])) OR (LY411575[Title/Abstract])) OR (LY-411575[Title/Abstract])) OR (LY411,575[Title/Abstract])) OR (LY 411,575[Title/Abstract])) OR (LY-411,575[Title/Abstract])) OR (LY 450139[Title/Abstract])) OR (LY450139[Title/Abstract]) | 47,692 |
| #13 | (((((((((((((((((((((((((((((((GRL 8234[Title/Abstract]) OR (GRL8234[Title/Abstract])) OR (GRL-8234[Title/Abstract])) OR (N-(2-(2,5-diphenyl-pyrrol-1-yl)-acetyl)guanidine[Title/Abstract])) OR (glutamyl-valyl-asparagyl-statine-valyl-alanyl-glutamyl-phenylalaninamide[Title/Abstract])) OR (EVNstatineVAEF-NH2[Title/Abstract])) OR (Glu-Val-Asn-statine-Val-Ala-Glu-Phe-NH2[Title/Abstract])) OR (N-benzyloxycarbonyl-valyl-leucyl-leucinal[Title/Abstract])) OR (Z-VLL-CHO[Title/Abstract])) OR (N-benzyloxycarbonyl-Val-Leu-leucinal[Title/Abstract])) OR (benzyl 5-((((5-aminopentyl)amino)carbonyl)methoxy)3-(((alpha-methyl-4-fluorobenzyl)amino)carbonyl)benzenesulfonate[Title/Abstract])) OR (benzyl ACAMFACBS[Title/Abstract])) OR (N-(1-(3,5-difluorobenzyl)-2-hydroxy-4-isobutylcarbamoylpentyl)-N',N'-dipropylisophthalamide[Title/Abstract])) OR (ylgucpd88[Title/Abstract])) OR (verubecestat[Title/Abstract])) OR (N-(3-(3-amino-5,6-dihydro-2,5-dimethyl-1,1-dioxido-2H-1,2,4-thiadiazin-5-yl)-4-fluorophenyl)-5-fluoro-2-pyridinecarboxamide[Title/Abstract])) OR (MK-8931[Title/Abstract])) OR (SCH-900931[Title/Abstract])) OR (N-(3-(2-amino-6H-1,3,4-thiadiazin-5-yl)-4-(benzyloxy)phenyl)-1-naphthamide hydrobromide[Title/Abstract])) OR (6-hydroxy-4-oxo-N-(10-((1,2,3,4-tetrahydroacridin-9-yl)amino)decyl)-4H-chromene-2-carboxamide[Title/Abstract])) OR (NB 216[Title/Abstract])) OR (NB216 cpd[Title/Abstract])) OR (NB-216[Title/Abstract])) OR (GSK188909[Title/Abstract])) OR (N-methyl-N-(3-((1-phenylethyl)aminocarbonyl)-5-((1-benzyl-4-cyclopropylamino-3-hydroxybut-2-yl)aminocarbonyl)phenyl) methanesulfonamide[Title/Abstract])) OR (N-methyl-PACBAC-MSA[Title/Abstract])) OR (Umibecestat[Title/Abstract])) OR (N-(6-((3R,6R)-5-Amino-3,6-dimethyl-6-(trifluoromethyl)-3,6-dihydro-2H-1,4-oxazin-3-yl)-5-fluoro-2-pyridinyl)-3-chloro-5-(trifluoromethyl)-2-pyridinecarboxamide[Title/Abstract])) OR (2-Pyridinecarboxamide, N-(6-((3R,6R)-5-amino-3,6-dihydro-3,6-dimethyl-6-(trifluoromethyl)-2H-1,4-oxazin-3-yl)-5-fluoro-2-pyridinyl)-3-chloro-5-(trifluoromethyl)-[Title/Abstract])) OR (CNP520[Title/Abstract])) OR (NB-360[Title/Abstract])) OR (2,3,4,4-tetramethyl-5-methylenecyclopent-2-enone[Title/Abstract]) | 115 |
| #14 | "Antibodies, Monoclonal"[Mesh] | 271,759 |
| #15 | ((Monoclonal Antibodies[Title/Abstract]) OR (Monoclonal Antibody[Title/Abstract])) OR (Antibody, Monoclonal[Title/Abstract]) | 203,290 |
| #16 | ((((((((((((PBT2 compound[Title/Abstract]) OR (5,7-dichloro-2-((dimethylamino)methyl)-8-hydroxyquinoline[Title/Abstract])) OR (tramiprosate[Title/Abstract])) OR (3-APS[Title/Abstract])) OR (3-aminopropanesulfonic acid[Title/Abstract])) OR (3-aminopropylsulfonic acid[Title/Abstract])) OR (homotaurine[Title/Abstract])) OR (3-amino-1-propanesulfonic acid[Title/Abstract])) OR (6-(4-methyl-1-(pyrimidin-2-ylmethyl)pyrrolidin-3-yl)-1-(tetrahydro-2H-pyran-4-yl)-1,5-dihydro-4H-pyrazolo(3,4-d)pyrimidin-4-one[Title/Abstract])) OR (6-((3S,4S)-4-methyl-1-(pyrimidin-2-ylmethyl)pyrrolidin-3-yl)-1-(tetrahydro-2H-pyran-4-yl)-1,5-dihydro-4H-pyrazolo(3,4-d)pyrimidin-4-one[Title/Abstract])) OR (PF-04447943[Title/Abstract])) OR (AN1792[Title/Abstract])) OR (CAD106[Title/Abstract]) | 473 |
| #17 | #14 OR #15 OR #16 | 358,551 |
| #18 | #11 OR #12 OR #13 OR #17 | 496,612 |
| #19 | #1 AND #4 AND #18 | **311** |

**Embase：**

| Search | Query | Results |
| --- | --- | --- |
| #1 | 'alzheimer disease'/exp OR 'alzheimer disease' | 243,218 |
| #2 | 'senile dementia':ab,kw,ti | 3851 |
| #3 | 'alzheimer dementia':ab,kw,ti | 72,004 |
| #4 | 'alzheimer dementias':ab,kw,ti | 6,285 |
| #5 | 'dementia, alzheimer':ab,kw,ti | 1,418 |
| #6 | 'dementia, senile':ab,kw,ti | 47 |
| #7 | 'dementia, alzheimer type':ab,kw,ti | 79 |
| #8 | 'alzheimer type dementia':ab,kw,ti | 937 |
| #9 | 'alzheimers disease':ab,kw,ti | 1,861 |
| #10 | 'alzheimer-type dementia (atd)':ab,kw,ti | 127 |
| #11 | 'alzheimer type dementia (atd)':ab,kw,ti | 127 |
| #12 | 'dementia, alzheimer-type (atd)':ab,kw,ti | 2 |
| #13 | 'alzheimer type senile dementia':ab,kw,ti | 28 |
| #14 | 'primary senile degenerative dementia':ab,kw,ti | 3 |
| #15 | 'dementia, primary senile degenerative':ab,kw,ti | 1 |
| #16 | 'alzheimer sclerosis':ab,kw,ti | 9,084 |
| #17 | 'sclerosis, alzheimer':ab,kw,ti | 450 |
| #18 | 'alzheimer syndrom':ab,kw,ti | 46 |
| #19 | 'alzheimer diseases':ab,kw,ti | 288 |
| #20 | 'alzheimers diseases':ab,kw,ti | 13 |
| #21 | 'senile dementia, alzheimer type':ab,kw,ti | 37 |
| #22 | 'acute confusional senile dementia':ab,kw,ti | 0 |
| #23 | 'senile dementia, acute confusional':ab,kw,ti | 0 |
| #24 | 'dementia, presenile':ab,kw,ti | 11 |
| #25 | 'presenile dementia':ab,kw,ti | 722 |
| #26 | 'alzheimer disease, late onset':ab,kw,ti | 3 |
| #27 | 'late onset alzheimer disease':ab,kw,ti | 515 |
| #28 | 'alzheimers disease, focal onset':ab,kw,ti | 0 |
| #29 | 'focal onset alzheimers disease':ab,kw,ti | 0 |
| #30 | 'familial alzheimer disease (fad)':ab,kw,ti | 102 |
| #31 | 'alzheimer disease, familial (fad)':ab,kw,ti | 0 |
| #32 | 'familial alzheimer diseases (fad)':ab,kw,ti | 0 |
| #33 | 'alzheimer disease, early onset':ab,kw,ti | 1 |
| #34 | 'early onset alzheimer disease':ab,kw,ti | 246 |
| #35 | 'presenile alzheimer dementia':ab,kw,ti | 571 |
| #36 | #1 OR #2 OR #3 OR #4 OR #5 OR #6 OR #7 OR #8 OR #9 OR #10 OR #11 OR #12 OR #13 OR #14 OR #15 OR #16 OR #17 OR #18 OR #19 OR #20 OR #21 OR #22 OR #23 OR #24 OR #25 OR #26 OR #27 OR #28 OR #29 OR #30 OR #31 OR #32 OR #33 OR #34 OR #35 | 259,051 |
| #37 | 'amyloid beta protein'/exp OR ' amyloid beta protein' | 68308 |
| #38 | 'amyloid beta peptides':ab,kw,ti | 748 |
| #39 | 'beta-peptides, amyloid':ab,kw,ti | 0 |
| #40 | 'alzheimer beta-protein':ab,kw,ti | 1 |
| #41 | 'beta-protein, alzheimer':ab,kw,ti | 1 |
| #42 | 'alzheimers abp':ab,kw,ti | 0 |
| #43 | 'abp, alzheimers':ab,kw,ti | 0 |
| #44 | 'alzheimer abp':ab,kw,ti | 43 |
| #45 | 'alzheimers abp':ab,kw,ti | 0 |
| #46 | 'alzheimers amyloid fibril protein':ab,kw,ti | 0 |
| #47 | 'protein, beta-amyloid':ab,kw,ti | 51 |
| #48 | 'amyloid beta-peptide':ab,kw,ti | 1935 |
| #49 | 'amyloid beta peptide':ab,kw,ti | 1935 |
| #50 | 'beta-peptide, amyloid':ab,kw,ti | 5 |
| #51 | 'beta amyloid protein':ab,kw,ti | 482 |
| #52 | 'beta-protein, amyloid':ab,kw,ti | 7 |
| #53 | 'amyloid beta proteins':ab,kw,ti | 57 |
| #54 | 'beta-proteins, amyloid':ab,kw,ti | 0 |
| #55 | 'amyloid fibril protein, alzheimers':ab,kw,ti | 0 |
| #56 | 'amyloid protein a4':ab,kw,ti | 7 |
| #57 | 'protein a4, amyloid':ab,kw,ti | 0 |
| #58 | 'beta amyloid':ab,kw,ti | 8189 |
| #59 | 'amyloid, beta':ab,kw,ti | 15376 |
| #60 | 'amyloid ad-ap':ab,kw,ti | 0 |
| #61 | 'ad-ap, amyloid':ab,kw,ti | 0 |
| #62 | #37 OR #38 OR #39 OR #40 OR #41 OR #42 OR #43 OR #44 OR #45 OR #46 OR #47 OR #48 OR #49 OR #50 OR #51 OR #52 OR #53 OR #54 OR #55 OR #56 OR #57 OR #58 OR #59 OR #60 OR #61 | 74614 |
| #63 | #36 AND #62 | 55324 |
| #64 | #63 AND 'randomized controlled trial'/de | 486 |
| #65 | 'immunoglobulin'/exp OR'immunoglobulin' | 855603 |
| #66 | 'immunoglobulins intravenous':ab,kw,ti | 85 |
| #67 | 'antibodies, intravenous':ab,kw,ti | 52 |
| #68 | 'intravenous antibodies':ab,kw,ti | 9 |
| #69 | 'intravenous immune globulin':ab,kw,ti | 1491 |
| #70 | 'immune globulin, intravenous':ab,kw,ti | 148 |
| #71 | 'intravenous immunoglobulins':ab,kw,ti | 5231 |
| #72 | 'intravenous ig':ab,kw,ti | 292 |
| #73 | 'iv immunoglobulins':ab,kw,ti | 282 |
| #74 | 'immunoglobulins, iv':ab,kw,ti | 44 |
| #75 | 'ivig':ab,kw,ti | 22012 |
| #76 | 'iv immunoglobulin':ab,kw,ti | 864 |
| #77 | 'immunoglobulin, iv':ab,kw,ti | 93 |
| #78 | 'intravenous immunoglobulin':ab,kw,ti | 21320 |
| #79 | 'immunoglobulin, intravenous':ab,kw,ti | 89 |
| #80 | 'flebogamma dif':ab,kw,ti | 25 |
| #81 | 'gamunex':ab,kw,ti | 128 |
| #82 | 'globulin n':ab,kw,ti | 150 |
| #83 | 'intraglobin':ab,kw,ti | 35 |
| #84 | 'intraglobin f':ab,kw,ti | 8 |
| #85 | 'intravenous immunoglobulins, human':ab,kw,ti | 0 |
| #86 | 'human intravenous immunoglobulins':ab,kw,ti | 60 |
| #87 | 'immunoglobulins, human intravenous':ab,kw,ti | 0 |
| #88 | 'immune globulin intravenous (human)':ab,kw,ti | 36 |
| #89 | 'immunoglobulins, intravenous, human':ab,kw,ti | 0 |
| #90 | 'human intravenous immunoglobulin':ab,kw,ti | 267 |
| #91 | 'immunoglobulin, human intravenous':ab,kw,ti | 1 |
| #92 | 'intravenous immunoglobulin, human':ab,kw,ti | 3 |
| #93 | 'gammagard':ab,kw,ti | 172 |
| #94 | 'gamimune':ab,kw,ti | 43 |
| #95 | 'gamimmune':ab,kw,ti | 8 |
| #96 | 'modified immune globulin (anti-echovirus antibody)':ab,kw,ti | 0 |
| #97 | 'privigen':ab,kw,ti | 217 |
| #98 | 'sandoglobulin':ab,kw,ti | 158 |
| #99 | 'venoglobulin':ab,kw,ti | 14 |
| #100 | 'venoglobulin i':ab,kw,ti | 8 |
| #101 | 'venimmune':ab,kw,ti | 0 |
| #102 | 'iveegam':ab,kw,ti | 6 |
| #103 | 'alphaglobin':ab,kw,ti | 502 |
| #104 | 'endobulin':ab,kw,ti | 26 |
| #105 | 'gamimune n':ab,kw,ti | 21 |
| #106 | 'gamimmune n':ab,kw,ti | 2 |
| #107 | 'gammonativ':ab,kw,ti | 6 |
| #108 | #65 OR #66 OR #67 OR #68 OR #69 OR #70 OR #71 OR #72 OR #73 OR #74 OR #75 OR #76 OR #77 OR #78 OR #79 OR #80 OR #81 OR #82 OR #83 OR #84 OR #85 OR #86 OR #87 OR #88 OR #89 OR #90 OR #91 OR #92 OR #93 OR #94 OR #95 OR #96 OR #97 OR #98 OR #99 OR #100 OR #101 OR #102 OR #103 OR #104 OR #105 OR #106 OR #107 | 859450 |
| #109 | 'secretase inhibitor'/exp OR'secretase inhibitor' | 5477 |
| #110 | 'monoclonal antibody'/exp OR'monoclonal antibody' | 308854 |
| #111 | 'antibodies, monoclonal':ab,kw,ti | 555 |
| #112 | 'monoclonal antibodies':ab,kw,ti | 128268 |
| #113 | 'antibody, monoclonal':ab,kw,ti | 198 |
| #114 | #110 OR #111 OR #112 OR #113 | 350143 |
| #115 | #62 OR #108 OR #109 OR #114 | 1229906 |
| #116 | #36 AND #115 | 61002 |
| #117 | #130 AND 'randomized controlled trial'/de | **590** |

**Cochrane:**

| Search | Query | Results |
| --- | --- | --- |
| #1 | MeSH descriptor: [Alzheimer Disease] explode all trees | 3874 |
| #2 | (Alzheimer Dementia):ab,kw,ti OR (Alzheimer Dementias):ab,kw,ti OR (Dementia, Alzheimer):ab,kw,ti OR (Alzheimer's Disease):ab,kw,ti OR (Dementia, Senile):ab,kw,ti OR (Senile Dementia):ab,kw,ti OR (Dementia, Alzheimer Type):ab,kw,ti OR (Alzheimer Type Dementia):ab,kw,ti OR (Alzheimer-Type Dementia (ATD)):ab,kw,ti OR (Alzheimer Type Dementia (ATD)):ab,kw,ti OR (Dementia, Alzheimer-Type (ATD)):ab,kw,ti OR (Alzheimer Type Senile Dementia):ab,kw,ti OR (Primary Senile Degenerative Dementia):ab,kw,ti OR (Dementia, Primary Senile Degenerative):ab,kw,ti OR (Alzheimer Sclerosis):ab,kw,ti OR (Sclerosis, Alzheimer):ab,kw,ti OR (Alzheimer Syndrome):ab,kw,ti OR (Alzheimer's Diseases):ab,kw,ti OR (Alzheimer Diseases):ab,kw,ti OR (Alzheimers Diseases):ab,kw,ti OR (Senile Dementia, Alzheimer Type):ab,kw,ti OR (Acute Confusional Senile Dementia):ab,kw,ti OR (Senile Dementia, Acute Confusional):ab,kw,ti OR (Dementia, Presenile):ab,kw,ti OR (Presenile Dementia):ab,kw,ti OR (Alzheimer Disease, Late Onset):ab,kw,ti OR (Late Onset Alzheimer Disease):ab,kw,ti OR (Alzheimer's Disease, Focal Onset):ab,kw,ti OR (Focal Onset Alzheimer's Disease):ab,kw,ti OR (Familial Alzheimer Disease (FAD)):ab,kw,ti OR (Alzheimer Disease, Familial (FAD)):ab,kw,ti OR (Familial Alzheimer Diseases (FAD)):ab,kw,ti OR (Alzheimer Disease, Early Onset):ab,kw,ti OR (Early Onset Alzheimer Disease):ab,kw,ti OR (Presenile Alzheimer Dementia):ab,kw,ti | 12857 |
| #3 | #1 OR #2 | 12857 |
| #4 | MeSH descriptor: [Amyloid beta-Peptides] explode all trees | 236 |
| #5 | (Amyloid beta Peptides):ab,kw,ti OR (beta-Peptides, Amyloid):ab,kw,ti OR (Alzheimer beta-Protein):ab,kw,ti OR (Alzheimer beta Protein):ab,kw,ti OR (beta-Protein, Alzheimer):ab,kw,ti OR (Alzheimer's ABP):ab,kw,ti OR (ABP, Alzheimer's):ab,kw,ti OR (Alzheimer ABP):ab,kw,ti OR (Alzheimers ABP):ab,kw,ti OR (Alzheimer's Amyloid Fibril Protein):ab,kw,ti OR (beta-Amyloid Protein):ab,kw,ti OR (Protein, beta-Amyloid):ab,kw,ti OR (beta Amyloid Protein):ab,kw,ti OR (Amyloid beta-Peptide):ab,kw,ti OR (Amyloid beta Peptide):ab,kw,ti OR (beta-Peptide, Amyloid):ab,kw,ti OR (Amyloid beta-Protein):ab,kw,ti OR (Amyloid beta Protein):ab,kw,ti OR (beta-Protein, Amyloid):ab,kw,ti OR (Amyloid beta-Proteins):ab,kw,ti OR (Amyloid beta Proteins):ab,kw,ti OR (beta-Proteins, Amyloid):ab,kw,ti OR (Amyloid Fibril Protein, Alzheimer's):ab,kw,ti OR (Amyloid Protein A4):ab,kw,ti OR (Protein A4, Amyloid):ab,kw,ti OR (beta Amyloid):ab,kw,ti OR (Amyloid, beta):ab,kw,ti OR (Amyloid AD-AP):ab,kw,ti OR (AD-AP, Amyloid):ab,kw,ti OR (Amyloid AD AP):ab,kw,ti | 947 |
| #6 | #4 OR #5 | 947 |
| #7 | MeSH descriptor: [Immunoglobulins, Intravenous] explode all trees | 912 |
| #8 | (Antibodies, Intravenous):ab,kw,ti OR (Intravenous Antibodies):ab,kw,ti OR (Immune Globulin, Intravenous):ab,kw,ti OR (Intravenous Immune Globulin):ab,kw,ti OR (Intravenous Immunoglobulins):ab,kw,ti OR (Intravenous IG):ab,kw,ti OR (IV Immunoglobulins):ab,kw,ti OR (Immunoglobulins, IV):ab,kw,ti OR (IVIG):ab,kw,ti OR (IV Immunoglobulin):ab,kw,ti OR (Immunoglobulin, IV):ab,kw,ti OR (Intravenous Immunoglobulin):ab,kw,ti OR (Immunoglobulin, Intravenous):ab,kw,ti OR (Flebogamma DIF):ab,kw,ti OR (Gamunex):ab,kw,ti OR (Globulin-N):ab,kw,ti OR (Globulin N):ab,kw,ti OR (Intraglobin):ab,kw,ti OR (Intraglobin F):ab,kw,ti OR (Intravenous Immunoglobulins, Human):ab,kw,ti OR (Human Intravenous Immunoglobulins):ab,kw,ti OR (Immunoglobulins, Human Intravenous):ab,kw,ti OR (Immune Globulin Intravenous (Human)):ab,kw,ti OR (Immunoglobulins, Intravenous, Human):ab,kw,ti OR (Human Intravenous Immunoglobulin):ab,kw,ti OR (Immunoglobulin, Human Intravenous):ab,kw,ti OR (Intravenous Immunoglobulin, Human):ab,kw,ti OR (Gammagard):ab,kw,ti OR (Gamimune):ab,kw,ti OR (Gamimmune):ab,kw,ti OR (Modified Immune Globulin (Anti-Echovirus Antibody)):ab,kw,ti OR (Privigen):ab,kw,ti OR (Sandoglobulin):ab,kw,ti OR (Venoglobulin):ab,kw,ti OR (Venoglobulin-I):ab,kw,ti OR (Venoglobulin I):ab,kw,ti OR (Venimmune):ab,kw,ti OR (Iveegam):ab,kw,ti OR (Alphaglobin):ab,kw,ti OR (Endobulin):ab,kw,ti OR (Gamimune N):ab,kw,ti OR (Gamimmune N):ab,kw,ti OR (Gammonativ) | 7647 |
| #9 | #7 OR #8 | 7647 |
| #10 | MeSH descriptor: [Antibodies, Monoclonal] explode all trees | 16088 |
| #11 | (Monoclonal Antibodies):ab,kw,ti OR (Monoclonal Antibody):ab,kw,ti OR (Antibody, Monoclonal):ab,kw,ti | 19337 |
| #12 | #10 OR #11 | 25294 |
| #13 | (DAPT cpd):ab,kw,ti OR (AZ1136):ab,kw,ti OR (AZ3303):ab,kw,ti OR (AZ4800):ab,kw,ti OR (begacestat):ab,kw,ti OR (benzyloxycarbonyl-isoleucyl-leucinal):ab,kw,ti OR (Z-IL-CHO):ab,kw,ti OR (Z-Ile-Leu-CHO):ab,kw,ti OR (GSI-XII cpd):ab,kw,ti OR (benzyloxycarbonyl-leucyl-leucyl-norleucinal):ab,kw,ti OR (GSI I cpd):ab,kw,ti OR (Z-Leu-Leu-Nle-CHO):ab,kw,ti OR (Z-LLNle-CHO):ab,kw,ti OR (GSI compound):ab,kw,ti OR (GSI-I cpd):ab,kw,ti OR (BIIB042):ab,kw,ti OR (BMS 708163):ab,kw,ti OR (BMS708163):ab,kw,ti OR (BMS-708163):ab,kw,ti OR (avagacestat):ab,kw,ti OR (BMS-986115):ab,kw,ti OR (BPN-15606):ab,kw,ti OR (curcumin-phenylalanine):ab,kw,ti OR (curcumin-valine):ab,kw,ti OR (dibenzazepine):ab,kw,ti OR (iminostilbene):ab,kw,ti OR (GSI XX):ab,kw,ti OR (ELN594):ab,kw,ti OR (FRM-36143):ab,kw,ti OR (L 685458):ab,kw,ti OR (GSI-X cpd):ab,kw,ti OR (L-685,458):ab,kw,ti OR (L-685458):ab,kw,ti OR (L 685,458):ab,kw,ti OR (LY900009):ab,kw,ti OR (MRK 003):ab,kw,ti OR (MRK003):ab,kw,ti OR (MRK-003):ab,kw,ti OR (MRK 560):ab,kw,ti OR (MRK560):ab,kw,ti OR (MRK-560):ab,kw,ti OR (MW167):ab,kw,ti OR (MW167 cpd):ab,kw,ti OR (DAPT butylester):ab,kw,ti OR (GSI-IX cpd):ab,kw,ti OR (DAPT peptide):ab,kw,ti OR (NN-DAPBB amide):ab,kw,ti OR (nirogacestat):ab,kw,ti OR (PF 03084014):ab,kw,ti OR (PF03084014):ab,kw,ti OR (PF-03084014):ab,kw,ti OR (SCH 1500022):ab,kw,ti OR (SCH1500022):ab,kw,ti OR (SCH-1500022):ab,kw,ti OR (SCH 900229):ab,kw,ti OR (SPI-1865):ab,kw,ti OR (tarenflurbil):ab,kw,ti OR (MPC-7869):ab,kw,ti OR (MPC7869):ab,kw,ti OR (flurizan):ab,kw,ti OR (TMP-001):ab,kw,ti OR (TMP001):ab,kw,ti OR (Semagacestat):ab,kw,ti OR (LY 411575):ab,kw,ti OR (LY411575):ab,kw,ti OR (LY-411575):ab,kw,ti OR (LY411,575):ab,kw,ti OR (LY 411,575):ab,kw,ti OR (LY-411,575):ab,kw,ti OR (LY 450139):ab,kw,ti OR (LY450139):ab,kw,ti | 128 |
| #14 | (GRL 8234):ab,kw,ti OR (GRL8234):ab,kw,ti OR (GRL-8234):ab,kw,ti OR (glutamyl-valyl-asparagyl-statine-valyl-alanyl-glutamyl-phenylalaninamide):ab,kw,ti OR (EVNstatineVAEF-NH2):ab,kw,ti OR (benzyl ACAMFACBS):ab,kw,ti OR (ylgucpd88):ab,kw,ti OR (verubecestat):ab,kw,ti OR (MK-8931):ab,kw,ti OR (SCH-900931):ab,kw,ti OR (NB 216):ab,kw,ti OR (NB216 cpd):ab,kw,ti OR (NB-216):ab,kw,ti OR (GSK188909):ab,kw,ti OR (Umibecestat):ab,kw,ti OR (CNP520):ab,kw,ti OR (NB-360):ab,kw,ti | 59 |
| #15 | (PBT2 compound):ab,kw,ti OR (tramiprosate):ab,kw,ti OR (PF-04447943):ab,kw,ti OR (AN1792):ab,kw,ti OR (CAD106):ab,kw,ti | 89 |
| #16 | #13 OR #14 OR #15 | 267 |
| #17 | #6 OR #9 OR #12 OR #16 | 31303 |
| #18 | #17 AND #3 | **1014** |

Clinicaltrials.gov：

| Search | Query | Results |
| --- | --- | --- |
| #1 | Status: All studies, condition or disease: Alzheimer Disease, and other terms: Immunoglobulins, Intravenous \| Study type: interventional (Clinical Trial) | 9 |
| #2 | Status: All studies, condition or disease: Alzheimer Disease, and other terms: (DAPT cpd) OR (AZ1136) OR (AZ3303) OR (AZ4800) OR (begacestat) OR (benzyloxycarbonyl-isoleucyl-leucinal) OR (Z-IL-CHO) OR (Z-Ile-Leu-CHO) OR (GSI-XII cpd) OR (benzyloxycarbonyl-leucyl-leucyl-norleucinal) OR (GSI I cpd) OR (Z-Leu-Leu-Nle-CHO) \| Study type: interventional (Clinical Trial) | 2 |
| #3 | Status: All studies, condition or disease: Alzheimer Disease, and other terms: (Z-LLNle-CHO) OR (GSI compound) OR (GSI-I cpd) OR (BIIB042) OR (BMS 708163) OR (BMS708163) OR (BMS-708163) OR (avagacestat) OR (BMS-986115) OR (BPN-15606) OR (curcumin-phenylalanine) OR (curcumin-valine) OR (dibenzazepine) OR (iminostilbene) \| Study type: interventional (Clinical Trial) | 12 |
| #4 | Status: All studies, condition or disease: Alzheimer Disease, and other terms: (GSI XX) OR (ELN594) OR (FRM-36143) OR (L 685458) OR (GSI-X cpd) OR (L-685,458) OR (L-685458) OR (L 685,458) OR (LY900009) OR (MRK 003) OR (MRK003) OR (MRK-003) OR (MRK 560) OR (MRK560) OR (MRK-560) \| Study type: interventional (Clinical Trial) | 0 |
| #5 | Status: All studies, condition or disease: Alzheimer Disease, and other terms: (MW167) OR (MW167 cpd) OR (DAPT butylester) OR (GSI-IX cpd) OR (DAPT peptide) OR (NN-DAPBB amide) OR (nirogacestat) OR (PF 03084014) OR (PF03084014) OR (PF-03084014) OR (SCH 1500022) \| Study type: interventional (Clinical Trial) | 0 |
| #6 | Status: All studies, condition or disease: Alzheimer Disease, and other terms: (SCH1500022) OR (SCH-1500022) OR (SCH 900229) OR (SPI-1865) OR (tarenflurbil) OR (MPC-7869) OR (MPC7869) OR (flurizan) OR (TMP-001) OR (TMP001) OR (Semagacestat) OR (LY 411575) OR (LY411575) OR (LY-411575) OR (LY411,575) OR (LY 411,575) \| Study type: interventional (Clinical Trial) | 6 |
| #7 | Status: All studies, condition or disease: Alzheimer Disease, and other terms: (LY-411,575) OR (LY 450139) OR (LY450139) \| Study type: interventional (Clinical Trial) | 5 |
| #8 | Status: All studies, condition or disease: Alzheimer Disease, and other terms: (GRL 8234) OR (GRL8234) OR (GRL-8234) OR (glutamyl-valyl-asparagyl-statine-valyl-alanyl-glutamyl-phenylalaninamide) OR (EVNstatineVAEF-NH2) OR (benzyl ACAMFACBS) OR (ylgucpd88) OR (verubecestat) \| Study type: interventional (Clinical Trial) | 5 |
| #9 | Status: All studies, condition or disease: Alzheimer Disease, and other terms: (MK-8931) OR (SCH-900931) OR (NB 216) OR (NB216 cpd) OR (NB-216) OR (GSK188909) OR (Umibecestat) OR (CNP520) OR (NB-360) \| Study type: interventional (Clinical Trial) | 8 |
| #10 | Status: All studies, condition or disease: Alzheimer Disease, and other terms: (PBT2 compound) OR (tramiprosate) OR (PF-04447943) OR (AN1792) OR (CAD106) \| Study type: interventional (Clinical Trial) | 16 |
| #11 | Status: All studies, condition or disease: Alzheimer Disease, and other terms: Amyloid beta-Peptides \| Study type: interventional (Clinical Trial) | 81 |
| #12 | Status: All studies, condition or disease: Alzheimer Disease, and other terms: Antibodies, Monoclonal, Study type: interventional (Clinical Trial) | 15 |
| #13 | Status: All studies, condition or disease: Alzheimer Disease, and other terms: Secretase Inhibitors \| Study type: interventional (Clinical Trial) | 7 |
| **Total** |  | **166** |

**Table S2: Characteristics of the included studies.**

| **Study** | **Region** | **Drugs type** | **Design** | **Age range(year)** | **Treatment group** | **No. of participants** | **Mean age (years)** | **Female (%)** | **MMSE at baseline** | **Study period** | **Outcome Events** |
| --- | --- | --- | --- | --- | --- | --- | --- | --- | --- | --- | --- |
| Ritchie et al.  2003 | Australia | MPAC | Double-blind, placebo-controlled, parallel-group randomized study | N/A | Clioquinol | 16 | N/A | N/A | N/A | 36 weeks | ADAS-cog, SAE, Death |
|  |  |  |  |  | placebo |  |  |  |  |  |  |
| Gilman et al.  2005 | US and Europe | Active Aβ Immunotherapy | Randomized, placebo-controlled, double-blind, phase IIa clinical trial | 50-85 | AN1792 | 300 | 71.0 ± 8.3 | 54.0 | 20.4 ± 3.3 | 15 months | ADAS-Cog, MMSE, AE, SAE, Death |
|  |  |  |  |  | Placebo | 72 | 72.4 ± 7.8 | 59.7 | 20.2 ± 3.5 |  |  |
| Fleisher et al.  2008  NCT00244322 | US | GSI | Multicenter, randomized, double-blind, dose-escalation, placebo-controlled trial | ≥ 50 | Semagacestat 100 mg | 22 | 70.8 ± 8.6 | 43.0 | 21.4 ± 3.8 | 26 weeks | ADAS-Cog, ADCS-ADL，AE, SAE |
|  |  |  |  |  | Semagacestat 140 mg | 14 | 68.1 ± 9.3 | 64.0 | 23.1 ± 3.4 |  |  |
|  |  |  |  |  | Placebo | 15 | 68.7 ± 10.1 | 33.0 | 18.9 ± 4.3 |  |  |
| Lannfelt et al.  2008  NCT00471211 | Sweden and Australia | MPAC | Phase IIa, double-blind, randomized, placebo-controlled trial | >55 | PBT2 50mg | 20 | 72.4 (58-83) | 45.0 | 23.2 ± 2.8 | 12 weeks | ADAS-Cog, MMSE, AE |
|  |  |  |  |  | PBT2 250mg | 29 | 72.1 (58-83) | 52.0 | 23.5 ± 2.7 |  |  |
|  |  |  |  |  | Placebo | 29 | 71.6 (60-83) | 52.0 | 22.2 ± 2.6 |  |  |
| Wilcock et al.  2008 | Canada and UK | GSM | Phase II, multicenter, double-blind study | ≥ 55 | Tarenflurbil 400mg | 62 | 73.4 ± 7.9 | 48.0 | 20.5 ± 3.6 | 12 months | ADAS-Cog, CDR-SB, MMSE, NPI, AE, Death |
|  |  |  |  |  | Tarenflurbil 800mg | 66 | 75.8 ± 7.8 | 52.0 | 21.3 ± 2.9 |  |  |
|  |  |  |  |  | Placebo | 61 | 74.4 ± 7.5 | 48.0 | 21.6 ± 3.1 |  |  |
| Green et al.  2009  NCT00105547 | US | GSM | Multicenter, randomized, double-blind, placebo-controlled trial | ≥ 55 | Tarenflurbil | 840 | 74.6 ± 8.5 | 49.4 | 23.3 ± 2.0 | 18 months | ADAS-Cog, CDR-SB, MMSE, ADCS-ADL, NPI, AE, SAE, Death |
|  |  |  |  |  | Placebo | 809 | 74.7 ± 8.4 | 52.5 | 23.3 ± 2.0 |  |  |
| Salloway et al.  2009  NCT00112073 | US | Monoclonal Antibodies | Phase II, multicenter, randomized, double-blind, placebo-controlled study | 50-85 | Bapineuzumab | 122 | 70.1 ± 9.06 | 50.0 | 20.9 ± 0.29 | 78 weeks | ADAS-Cog, CDR-SB, MMSE, AE, SAE, Death |
|  |  |  |  |  | Placebo | 107 | 67.9 ± 8.79 | 59.8 | 20.7 ± 0.3 |  |  |
| Aisen et al.  2011 | North America | Tramiprosate | Randomized, double-blind, placebo-controlled, parallel-group study | ≥ 50 | Tramiprosate 100 mg | 335 | 73.8 (50-92) | 49.9 | 21.1 (15.0-26.0) | 78 weeks | ADAS-Cog, CDR-SB, AE, SAE, Death |
|  |  |  |  |  | Tramiprosate 150 mg | 329 | 73.6 (48-94) | 55.9 | 21.1 (15.0-26.0) |  |  |
|  |  |  |  |  | Placebo | 341 | 74.2 (51-92) | 53.4 | 21.0 (12.0-26.0) |  |  |
| Farlow et al.  2012  NCT00329082 | US | Monoclonal Antibodies | Phase II, randomized, double-blind, placebo-controlled clinical trial | ≥ 55 | Solanezumab 100mgQ4W | 10 | 71.2 ± 9.2 | 53.8 | 20.2 ± 3.8 | 12 weeks | ADAS-Cog |
|  |  |  |  |  | Solanezumab 100mgQW | 11 |  |  |  |  |  |
|  |  |  |  |  | Solanezumab 400mgQ4W | 10 |  |  |  |  |  |
|  |  |  |  |  | Solanezumab 400mgQW | 11 |  |  |  |  |  |
|  |  |  |  |  | Placebo | 10 |  |  |  |  |  |
| Coric et al.  2012  NCT00810147 | US, Denmark, Finland, and Sweden | GSI | Multicenter, randomized, double-blind, placebo-controlled, 5-arm, fixed-dose, parallel-group study | 50-90 | Avagacestat 25 mg | 42 | 73.6 ± 8.2 | 38.1 | 21.4 ± 3.6 | 24 weeks | ADAS-Cog, CDR-SB, MMSE, ADCS-ADL |
|  |  |  |  |  | Avagacestat 50 mg | 41 | 74.4 ± 8.8 | 43.9 | 21.2 ± 4.0 |  |  |
|  |  |  |  |  | Avagacestat 100 mg | 42 | 72.9 ± 8.1 | 57.1 | 21.1 ± 4.4 |  |  |
|  |  |  |  |  | Avagacesta 125 mg | 42 | 74.0 ± 8.1 | 40.5 | 21.6 ± 4.4 |  |  |
|  |  |  |  |  | Placebo | 42 | 73.7 ± 10.6 | 59.5 | 21.5 ± 4.4 |  |  |
| Dodel et al.  2013  NCT00812565 | USA and Germany | Intravenous Immunoglobulin | Double-blind, block-randomised, placebo-controlled, parallel group, multicentre trial | 50-85 | Intravenous immunoglobulin | 41 | 69.4 ± 7.3 | 37.0 | 21.3 ± 2.8 | 24 weeks | ADAS-Cog, CDR-SB, MMSE, ADCS-ADL, AE, SAE, Death |
|  |  |  |  |  | Placebo | 14 | 72.0 ± 10.2 | 64.0 | 21.9 ± 2.4 |  |  |
| Doody et al.  2013  NCT00594568 | Global | GSI | Double-blind, placebo-controlled trial | ≥ 55 | Semagacestat 100 mg | 506 | 73.0 ± 8.0 | 55.0 | 20.8 ± 3.5 | 76 weeks | ADAS-Cog, CDR-SB, MMSE, ADCS-ADL, NPI, AE, SAE, Death |
|  |  |  |  |  | Semagacestat 140 mg | 527 | 73.3 ± 8.5 | 50.0 | 20.7 ± 3.5 |  |  |
|  |  |  |  |  | Placebo | 501 | 73.3 ± 8.1 | 55.0 | 20.9 ± 3.6 |  |  |
| Doody et al.  2014  EXPEDITION 1  NCT00905372 | Global | Monoclonal Antibodies | Phase III, randomized, double-blind, placebo-controlled trials | ≥ 55 | Solanezumab | 506 | 75.0 ± 7.9 | 59.1 | 21 ± 4 | 18 months | ADAS-Cog, CDR-SB, MMSE, ADCS-ADL, NPI, Death |
|  |  |  |  |  | Placebo | 506 | 74.4 ± 8.0 | 56.7 | 21 ± 3 |  |  |
| Doody et al.  2014  EXPEDITION 2  NCT00904683 | Global | Monoclonal Antibodies | Phase III, randomized, double-blind, placebo-controlled trials | ≥ 55 | Solanezumab | 521 | 72.5 ± 8.0 | 54.3 | 21 ± 3 | 18 months | ADAS-Cog, CDR-SB, MMSE, ADCS-ADL, NPI, Death |
|  |  |  |  |  | Placebo | 519 | 72.4 ± 7.8 | 55.1 | 21 ± 3 |  |  |
| Salloway et al.  2014  Study 302  NCT00574132 | US | Monoclonal Antibodies | Double-blind, randomized, placebo-controlled, phase III, trials | 50-88 | Bapineuzumab | 658 | 72.0 ± 8.0 | 54.4 | 20.8 ± 3.1 | 78 weeks | ADAS-Cog, CDR-SB, MMSE, AE, SAE, Death |
|  |  |  |  |  | Placebo | 432 | 72.3 ± 8.4 | 56.0 | 20.7 ± 3.2 |  |  |
| Salloway et al.  2014  Study 301  NCT00575055 | US | Monoclonal Antibodies | Double-blind, randomized, placebo-controlled, phase III, trials | 50-88 | Bapineuzumab 0.5mg/kg | 314 | 73.1 ± 9.3 | 52.5 | 21.2 ± 3.4 | 78 weeks | ADAS-Cog, CDR-SB, MMSE, AE, SAE, Death |
|  |  |  |  |  | Bapineuzumab 1.0mg/kg | 307 | 73.5 ± 9.1 | 57.0 | 21.2 ± 3.3 |  |  |
|  |  |  |  |  | Placebo | 493 | 71.9 ± 10.1 | 50.3 | 21.2 ± 3.2 |  |  |
| Arai et al.  2015  NCT00752232  NCT00959192 | Japan | Active Aβ Immunotherapy | Phase IIa, randomized, third-party, unblinded multiple ascending-dose study | 50-85 | ACC-001 3μg + QS-21 | 6 | 71.33 ± 9.35 | 16.7 | N/A | 78 weeks | ADAS-Cog, MMSE, AE, SAE, Death |
|  |  |  |  |  | ACC-001 10μg | 6 | 69.5 ± 6.92 | 66.7 |  |  |  |
|  |  |  |  |  | ACC-001 10μg + QS-21 | 6 | 73.0 ± 7.67 | 83.3 |  |  |  |
|  |  |  |  |  | ACC-001 30 µg | 6 | 70.17 ± 3.13 | 66.7 |  |  |  |
|  |  |  |  |  | ACC-00130 µg + QS-21 | 6 | 66.0 ± 10.79 | 33.3 |  |  |  |
|  |  |  |  |  | QS-21 | 6 | 67.0 ± 9.19 | 66.7 |  |  |  |
|  |  |  |  |  | Placebo | 4 | 69.5 ± 4.80 | 75.0 |  |  |  |
| Farlow et al.  2015  NCT00733863  NCT00795418 | France, Sweden, Switzerland, UK and US | Active Aβ Immunotherapy | Phase IIa, multicenter, randomized, double-blind, placebo-controlled, parallel-group study | 40-85 | CAD106 | 47 | 68.2 ± 8.2 | 38.3 | 22.5 ± 2.1 | 56 weeks | ADAS-Cog, AE, SAE, Death |
|  |  |  |  |  | Placebo | 11 | 66.3 ± 6.9 | 45.5 | 22.8 ± 2.3 |  |  |
| Coric et al.  2015  NCT00890890 | Global | GSI | Multicenter, double-blind, 2-arm, placebo-controlled, parallel- group, randomized clinical trial | 45-90 | Avagacestat | 132 | 71.9 ± 7.63 | 54.7 | 27.0 ± 1.91 | 104 weeks | ADAS-Cog, CDR-SB, MMSE, ADCS-ADL, AE, SAE |
|  |  |  |  |  | Placebo | 131 | 71.6 ± 7.78 | 52.0 | 27.1 ± 1.67 |  |  |
| Dyck et al.  2016  NCT01227564 | US | Active Aβ Immunotherapy | Phase II, multicenter, randomized, third-party unblinded, placebo-controlled, parallel-group trial | 50-80 | ACC-001 3μg | 22 | 66.8 ± 7.31 | 50.0 | 27.3 ± 1.64 | 24 months | ADAS-Cog, CDR-SB, MMSE, NPI, AE, SAE |
|  |  |  |  |  | ACC-001 10 μg | 20 | 68.5 ± 7.02 | 65.0 | 27.7 ± 1.63 |  |  |
|  |  |  |  |  | Placebo | 21 | 69.6 ± 6.82 | 42.9 | 28.0 ± 1.47 |  |  |
| Delnomdedieu et al.  2016  NCT01193608 | US and Korea | Monoclonal Antibodies | Randomized, double-blind, placebo-controlled, dose-escalation study | 50-89 | AAB-003 0.5mg/kg | 6 | 64.5 ± 6.4 | 66.7 | 22.7 ± 1.37 | 39 weeks | ADAS-Cog, CDR-SB, MMSE, NPI, AE, SAE, Death |
|  |  |  |  |  | AAB-003 1 mg/kg | 6 | 67.2 ± 7.4 | 50.0 | 22.5 ± 2.43 |  |  |
|  |  |  |  |  | AAB-003 2 mg/kg | 16 | 70.7 ± 10.8 | 87.5 | 20.8 ± 3.71 |  |  |
|  |  |  |  |  | AAB-003 4 mg/kg | 17 | 71.5 ± 8.8 | 52.9 | 20.1 ± 3.00 |  |  |
|  |  |  |  |  | AAB-003 8 mg/kg | 24 | 64.5 ± 7.6 | 58.3 | 21.2 ± 2.82 |  |  |
|  |  |  |  |  | Placebo | 19 | 71.1 ± 7.6 | 42.1 | 21.2 ± 3.78 |  |  |
| Pasquier et al.  2016  NCT00479557 NCT00498602 | US and Europe | Active Aβ Immunotherapy | Phase IIa, multicenter, randomized, third-party–unblinded, adjuvant- and placebo-controlled, multiple ascending–dose study | 50-85 | ACC-001 3 μg + QS-21 | 36 | 65.7 ± 7.8 | 44.4 | 21.4 ± 3.0 | 104w | ADAS-Cog, CDR-SB, MMSE, NPI, AE, SAE, Death |
|  |  |  |  |  | ACC-001 10 μg + QS-21 | 61 | 69.3 ± 9.6 | 47.5 | 21.4 ± 3.4 |  |  |
|  |  |  |  |  | ACC-001 30 μg + QS-21 | 40 | 69.0 ± 9.3 | 55.0 | 21.0 ± 2.9 |  |  |
|  |  |  |  |  | ACC-001 10μg | 35 | 71.9 ± 9.0 | 54.3 | 21.6 ± 3.2 |  |  |
|  |  |  |  |  | ACC-001 30μg | 12 | 70.9 ± 9.6 | 66.7 | 21.8 ± 3.6 |  |  |
|  |  |  |  |  | QS-21 | 44 | 68.8 ± 8.1 | 70.5 | 21.7 ± 3.1 |  |  |
|  |  |  |  |  | Placebo | 17 | 69.4 ± 7.9 | 82.4 | 21.2 ± 3.2 |  |  |
| Vandenberghe et al.  2016  Study 3000  NCT00667810 | Global | Monoclonal Antibodies | Phase III, multicenter, randomized, double-blind, placebo-controlled trials | 50-88 | Bapineuzumab 0.5mg/kg | 255 | 71.1 | 55.7 | 20.8 ± 3.2 | 18 months | ADAS-Cog, CDR-SB, MMSE, NPI, AE, SAE |
|  |  |  |  |  | Bapineuzumab 1.0mg/kg | 253 | 70.7 | 57.3 | 20.8 ± 3.1 |  |  |
|  |  |  |  |  | Placebo | 328 | 69.7 | 57.9 | 20.8 ± 3.1 |  |  |
| Vandenberghe et al.  2016  Study 3001  NCT00676143 | Global | Monoclonal Antibodies | Phase III, multicenter, randomized, double-blind, placebo-controlled trials | 50-88 | Bapineuzumab | 650 | 70.9 | 64.5 | 20.9 ± 3.1 | 18 months | ADAS-Cog, CDR-SB, MMSE, NPI, AE, SAE |
|  |  |  |  |  | Placebo | 431 | 70.2 | 60.1 | 21.0 ± 3.0 |  |  |
| Vandenberghe et al.  2016  NCT01097096 | Global | Active Aβ Immunotherapy | Phase II, randomized, double-blind, placebo-controlled, multicenter study | < 85 | CAD106 150 μg | 69 | 67.7 ± 9.0 | 46.4 | 22.8 ± 2.2 | 78 weeks | ADAS-Cog, CDR-SB, MMSE, ADCS-ADL, NPI, AE, SAE, Death |
|  |  |  |  |  | CAD106 450 μg | 37 | 66.3 ± 9.4 | 64.9 | 23.2 ± 2.2 |  |  |
|  |  |  |  |  | Placebo | 15 | 68.0 ± 8.4 | 53.3 | 22.1 ± 2.2 |  |  |
| Landen et al.  2017 | Sweden | Monoclonal Antibodies | Phase II, double-blind, randomized, placebo-controlled study | ≥50 | Ponezumab (Cohort Q) | 12 | 65.1 ± 7.4 | 33.3 | 22.5 ± 2.75 | 18 months | ADAS-Cog, AE, SAE, Death |
|  |  |  |  |  | Placebo | 6 | 71.3 ± 8.5 | 50.0 | 20.8 ± 2.99 |  |  |
|  |  |  |  |  | Ponezumab (Cohort M) | 12 | 69.8 ± 7.5 | 25.0 | 21.2 ± 3.04 |  |  |
|  |  |  |  |  | Placebo | 6 | 65.8 ± 8.3 | 83.3 | 22.5 ± 4.04 |  |  |
| Ostrowitzki et al.  2017  NCT01224106 | Global | Monoclonal Antibodies | Phase III, multicenter, randomized, double-blind, placebo-controlled, parallel-group study | 50-85 | Gantenerumab 105mg | 271 | 70.3 ± 7.0 | 58.5 | 25.7 ± 2.3 | 2 years | ADAS-Cog, CDR-SB, MMSE, AE, SAE, Death |
|  |  |  |  |  | Gantenerumab 225mg | 260 | 71.3 ± 7.1 | 56.1 | 25.7 ± 2.2 |  |  |
|  |  |  |  |  | Placebo | 266 | 69.5 ± 7.5 | 56.0 | 25.7 ± 2.1 |  |  |
| Relkin et al.  2017  NCT00818662 | US and Canada | Intravenous Immunoglobulin | Randomized, parallel-group, placebo-controlled trial | 50-89 | Intravenous immunoglobulin 0.4g/kg | 129 | 70.6 ± 9.7 | 54.3 | 21.3 ± 3.2 | 18 months | ADAS-Cog, ADCS-ADL, NPI, AE, SAE, Death |
|  |  |  |  |  | Intravenous immunoglobulin 0.2g/kg | 138 | 70.1 ± 8.3 | 55.8 | 21.5 ± 3.1 |  |  |
|  |  |  |  |  | Placebo | 123 | 70.2 ± 9.9 | 53.7 | 21.1 ± 3.2 |  |  |
| Cummings et al.  2018  NCT01343966 | North America and Europe | Monoclonal Antibodies | Phase II, multicenter, randomized, double-blind, placebo-controlled, parallel-group study | 50-80 | Crenezumab SC | 122 | 71.2 ± 6.3 | 54.1 | 21.7 ± 2.8 | 73 weeks | ADAS-Cog, CDR-SB, ADCS-ADL, AE, SAE, Death |
|  |  |  |  |  | Placebo SC | 62 | 70.3 ± 7.2 | 48.4 | 21.5 ± 2.6 |  |  |
|  |  |  |  |  | Crenezumab IV | 165 | 70.9 ± 6.9 | 50.9 | 21.9 ± 2.7 |  |  |
|  |  |  |  |  | Placebo IV | 84 | 69.9 ± 7.1 | 57.1 | 21.6 ± 2.5 |  |  |
| Egan et al.  2018  NCT01739348 | Global | BACE-1 Inhibitor | Randomized, double-blind, placebo-controlled trial | 55-85 | Verubecestat 12mg | 652 | 71.3 ± 7.4 | 53.7 | 20.4 ± 3.3 | 78 weeks | ADAS-Cog, CDR-SB, MMSE, ADCS-ADL, NPI, AE, SAE, Death |
|  |  |  |  |  | Verubecestat 40mg | 652 | 71.8 ± 7.6 | 58.1 | 20.2 ± 3.3 |  |  |
|  |  |  |  |  | Placebo | 653 | 72.4 ± 7.6 | 54.2 | 20.3 ± 3.3 |  |  |
| Honig et al.  2018  NCT01900665 | Global | Monoclonal Antibodies | Double-blind, placebo-controlled, phase III trial | 55-90 | Solanezumab | 1057 | 72.7 ± 7.8 | 56.8 | 22.8 ± 2.8 | 76 weeks | ADAS-Cog, CDR-SB, MMSE, ADCS-ADL, AE, SAE, Death |
|  |  |  |  |  | Placebo | 1072 | 73.3 ± 8.0 | 58.9 | 22.6 ± 2.9 |  |  |
| Salloway et al.  2018  NCT01397578 | US, Spain, and France | Monoclonal Antibodies | Double-blind, placebo-controlled, randomized phase II study | 50-80 | Crenezumab SC | 26 | 66.7 ± 9.5 | 53.8 | 21.5 ± 2.4 | 73 weeks | ADAS-Cog, CDR-SB, AE, SAE, Death |
|  |  |  |  |  | Placebo SC | 13 | 68.9 ± 8.3 | 61.5 | 22.3 ± 2.4 |  |  |
|  |  |  |  |  | Crenezumab IV | 35 | 71.4 ± 7.1 | 68.6 | 20.8 ± 2.3 |  |  |
|  |  |  |  |  | Placebo IV | 17 | 69.8 ± 7.7 | 35.3 | 20.5 ± 2.2 |  |  |
| Egan et al.  2019  NCT01953601 | Global | BACE-1 Inhibitor | Randomized, double-blind, placebo-controlled trial | 50-85 | Verubecestat 12mg | 485 | 71.7 ± 7.1 | 47.4 | 26.3 ± 1.8 | 104 weeks | ADAS-Cog, CDR-SB, MMSE, ADCS-ADL, NPI, AE, SAE, Death |
|  |  |  |  |  | Verubecestat 40mg | 484 | 71.0 ± 7.4 | 50.4 | 26.4 ± 1.7 |  |  |
|  |  |  |  |  | Placebo | 485 | 71.6 ± 7.1 | 44.0 | 26.3 ± 1.8 |  |  |
| Wessels et al.  2019  AMARANTH  NCT02245737 | Global | BACE-1 Inhibitor | Multicenter, randomized, double-blind, placebo-controlled, parallel-group trial | 55-85 | Lanabecestat 20mg | 739 | 71.2 ± 7.5 | 53.5 | 23.7 ± 2.6 | 24 months | ADAS-Cog, CDR-SB, MMSE, NPI, AE, SAE, Death |
|  |  |  |  |  | Lanabecestat 50mg | 739 | 71.2 ± 7.0 | 52.0 | 23.7 ± 2.6 |  |  |
|  |  |  |  |  | Placebo | 740 | 71.4 ± 6.9 | 53.8 | 23.8 ± 2.6 |  |  |
| Wessels et al.  2019  DAYBREAK-ALZ  NCT02783573 | Global | BACE-1 Inhibitor | Randomized, double-blind, placebo-controlled and delayed-Start Study | 55-85 | Lanabecestat 20mg | 590 | 72.3 ± 7.0 | 56.8 | 22.9 ± 1.8 | 78 weeks | ADAS-Cog, CDR-SB, MMSE, NPI, AE, SAE, Death |
|  |  |  |  |  | Lanabecestat 50mg | 570 | 72.6 ± 7.0 | 59.6 | 22.8 ± 1.9 |  |  |
|  |  |  |  |  | Placebo | 562 | 72.1 ± 7.1 | 61.9 | 22.8 ± 1.9 |  |  |
| Lo et al.  2021  NCT02791191 | US, Canada, Australia and Japan | BACE-1 Inhibitor | Double-blind, randomized, placebo-controlled trial | 55-85 | LY3202626 3mg | 55 | 73.91 ± 6.72 | 52.7 | N/A | 52 weeks | ADAS-Cog, MMSE, NPI, AE, SAE, Death |
|  |  |  |  |  | LY3202626 12mg | 128 | 72.73 ± 7.15 | 67.2 |  |  |  |
|  |  |  |  |  | Placebo | 133 | 72.54 ± 7.8 | 57.9 |  |  |  |
| Mintun et al.  2021  NCT03367403 | US and Canada | Monoclonal Antibodies | Multicenter, randomized, double-blind, placebo-controlled phase III trial | 60-85 | Donanemab | 131 | 75.0 ± 5.6 | 51.9 | 23.6 ± 3.1 | 76 weeks | ADAS-Cog, CDR-SB, MMSE, AE, SAE, Death |
|  |  |  |  |  | Placebo | 126 | 75.4 ± 5.4 | 51.6 | 23.7 ± 2.9 |  |  |
| Swanson et al.  2021  NCT01767311 | Global | Monoclonal Antibodies | Multicenter, double-blind, placebo-controlled clinical trial | 50-90 | Lecanemab 2.5 mg/kg biweekly | 52 | 70.5 ± 8.0 | 50.0 | 25.7 ± 2.5 | 18 months | ADAS-Cog, CDR-SB, AE, SAE, Death |
|  |  |  |  |  | Lecanemab 5 mg/kg monthly | 48 | 70.7 ± 6.5 | 50.0 | 25.3 ± 2.6 |  |  |
|  |  |  |  |  | Lecanemab 5 mg/kg biweekly | 89 | 71.7 ± 7.1 | 54.0 | 25.6 ± 2.3 |  |  |
|  |  |  |  |  | Lecanemab 10 mg/kg Monthly | 246 | 71.0 ± 6.6 | 45.0 | 25.7 ± 2.4 |  |  |
|  |  |  |  |  | Lecanemab 10 mg/kg biweekly | 152 | 72.7 ± 7.0 | 42.0 | 25.6 ± 2.4 |  |  |
|  |  |  |  |  | Placebo | 238 | 71.8 ± 7.0 | 58.0 | 26.0 ± 2.3 |  |  |
| Haeberlein et al.  2022  EMERGE  NCT02484547 | Global | Monoclonal Antibodies | Randomized, double-blind, placebo-controlled, global, phase III study | 50-85 | Aducanumab low dose | 543 | 70.6 ± 7.4 | 50.0 | 26.3 ± 1.7 | 78 weeks | ADAS-Cog, CDR-SB, MMSE, ADCS-ADL, AE, SAE, Death |
|  |  |  |  |  | Aducanumab high dose | 547 | 70.6 ± 7.5 | 52.0 | 26.3 ± 1.7 |  |  |
|  |  |  |  |  | Placebo | 548 | 70.8 ± 7.4 | 53.0 | 26.4 ± 1.8 |  |  |
| Haeberlein et al.  2022  ENGAGE  NCT02477800 | Global | Monoclonal Antibodies | Randomized, double-blind, placebo-controlled, global, phase III studies | 50-85 | Aducanumab low dose | 547 | 70.4 ± 7.0 | 52.0 | 26.4 ± 1.8 | 78 weeks | ADAS-Cog, CDR-SB, MMSE, ADCS-ADL, AE, SAE, Death |
|  |  |  |  |  | Aducanumab high dose | 555 | 70.0 ± 7.7 | 53.0 | 26.4 ± 1.8 |  |  |
|  |  |  |  |  | Placebo | 545 | 69.8 ± 7.7 | 53.0 | 26.4 ± 1.7 |  |  |
| Ostrowitzki et al.  2022  CREAD  NCT02670083 | Global | Monoclonal Antibodies | Phase III multicenter randomized double-blind placebo-controlled parallel-group study | 50-85 | Crenezumab | 404 | 71.0 ± 7.9 | 58.4 | 23.7 ± 3.0 | 100 weeks | ADAS-Cog, CDR-SB, MMSE, ADCS-ADL, NPI, AE, SAE, Death |
|  |  |  |  |  | Placebo | 409 | 70.3 ± 8.4 | 60.4 | 23.4 ± 2.9 |  |  |
| Ostrowitzki et al.  2022  CREAD 2  NCT03114657 | Global | Monoclonal Antibodies | Phase III multicenter randomized double-blind placebo-controlled parallel-group study | 50-85 | Crenezumab | 407 | 71.1 ± 7.5 | 56.8 | 23.6 ± 2.8 | 100 weeks | ADAS-Cog, CDR-SB, MMSE, ADCS-ADL, NPI, AE, SAE, Death |
|  |  |  |  |  | Placebo | 399 | 70.7 ± 7.9 | 56.4 | 23.5 ± 2.9 |  |  |
| Christopher et al.  2023  NCT03887455 | Global | Monoclonal Antibodies | Multicenter, double-blind, placebo-controlled, parallel-group trial | 50-90 | Lecanemab | 859 | 71.4 ± 7.9 | 51.6 | 25.5 ± 2.2 | 18 months | ADAS-Cog, CDR-SB, ADCS-ADL, AE, SAE, Death |
|  |  |  |  |  | Placebo | 875 | 71.0 ± 7.8 | 53.0 | 25.6 ± 2.2 |  |  |
| NCT00722046 | US, Canada, Australia, Korea, Belgium and UK | Monoclonal Antibodies | Phase II multicenter randomized double-blind placebo-controlled study | ≥ 50 | Ponezumab 0.1 mg/kg | 25 | 70.8 ± 8.2 | 48 | N/A | 18 months | ADAS-Cog, AE, SAE, Death |
|  |  |  |  |  | Ponezumab 0.5 mg/kg | 25 | 71.9 ± 9.4 | 60 |  |  |  |
|  |  |  |  |  | Ponezumab 1 mg/kg | 25 | 72.2 ± 8.4 | 44 |  |  |  |
|  |  |  |  |  | Placebo | 24 | 70.0 ± 7.8 | 54.2 |  |  |  |
|  |  |  |  |  | Ponezumab 3 mg/kg | 32 | 70.5 ± 8.9 | 62.5 |  |  |  |
|  |  |  |  |  | Ponezumab 8.5 mg/kg | 31 | 71.8 ± 7.3 | 54.8 |  |  |  |
|  |  |  |  |  | Placebo | 32 | 70.4 ± 10.3 | 53.1 |  |  |  |
| NCT00762411 | Global | GSI | Randomized placebo-controlled, parallel-group study | ≥ 55 | Semagacestat 140 mg | 555 | 73.4 ± 8.0 | 56.9 | N/A | 76 weeks | ADAS-Cog, ADCS-ADL, AE, SAE, Death |
|  |  |  |  |  | Placebo | 553 | 73.0 ± 8.0 | 59.1 |  |  |  |
| NCT01524887 | Global | Intravenous Immunoglobulin | Phase III randomized double-blind, placebo-controlled Study | 50-89 | Intravenous immunoglobulin 0.4g/kg | 83 | 72.0 ± 8.36 | 51.8 | N/A | 18 months  (Actual time frame: 9 months) | ADAS-Cog, ADCS-ADL, NPI, AE, SAE |
|  |  |  |  |  | Intravenous immunoglobulin 0.2g/kg | 85 | 69.9 ± 8.59 | 43.5 |  |  |  |
|  |  |  |  |  | Placebo | 83 | 70.6 ± 9.98 | 62.7 |  |  |  |
| NCT01561430 | US, Italy, Ja pan, Spain, Netherlands | BACE-1 Inhibitor | Phase I/Phase II randomized, parallel-group study | ≥ 55 | Lanabecestat 15mg | 9 | 65.59 ± 7.57 | 55.6 | N/A | 26 weeks | ADAS-Cog, CDR-SB, MMSE, AE, SAE |
|  |  |  |  |  | Lanabecestat 35mg | 23 | 72.5 ± 8.38 | 43.5 |  |  |  |
|  |  |  |  |  | Lanabecestat 70mg | 18 | 70.23 ± 8.6 | 38.9 |  |  |  |
|  |  |  |  |  | Placebo | 20 | 67.73 ± 7.13 | 55.0 |  |  |  |
| NCT02956486 | Global | BACE-1 Inhibitor | Phase III placebo-controlled, double-blind, parallel-group study | 50-85 | Elenbecestat | 1108 | 71.9 ± 7.18 | 48.6 | N/A | 24 months | ADAS-Cog, CDR-SB, MMSE, AE, SAE, Death |
|  |  |  |  |  | Placebo | 1104 | 72.1 ± 7.09 | 53.6 |  |  |  |

Data are mean ±SD, mean or mean (range). N/A: Not applicable; MPAC: metal-protein–attenuating compounds; ADAS-Cog: Alzheimer's Disease Assessment Scale–Cognitive Subscale; CDR-SB: Clinical Dementia Rating–Sum of Boxes; MMSE: Mini-Mental State Examination; ADCS-ADL: Alzheimer’s Disease Cooperative Study–Activities of Daily Living; NPI: Neuropsychiatric Inventory; AE: adverse events; SAE: serious adverse events; GSI: γ-Secretase Inhibitor; GSM: γ-Secretase Modulators.

**Table S3: Inclusion, exclusion criteria, study design and outcome assessments of the included studies**

| **Trials** | **Ritchie et al.** |
| --- | --- |
| ***Inclusion*** ***Criteria*** | Criteria for participation in the study included informed consent; a diagnosis of probable AD by means of criteria of the National Institute of Neurological and Communicative Disorders and Stroke and the Alzheimer's Disease and Related Disorders Association; ADAS-cog score of 20 to 45; MMSE score of 10 to 24; and receipt of a 5- to 10-mg dose of donepezil for at least 6 months. |
| ***Study Design*** | The study was a double-blind, placebo-controlled, parallel-group randomized design. Thirty-six patients and their caregivers were recruited. The duration of the study was 36 weeks. The oral dosage of clioquinol was 125 mg twice daily from weeks 0 to 12, 250 mg twice daily from weeks 13 to 24, and 375 mg twice daily from weeks 25 to 36. |
| ***Efficacy Outcomes*** | The primary efficacy variable was change from the baseline score on the ADAS-cog at weeks 4, 12, 24, and 36. Plasma Aβ, zinc, and copper levels were all measured every 4 weeks. |
| ***Safety Outcomes*** | Serious adverse events and death. |

| **Trials** | **Gilman et al.** |
| --- | --- |
| ***Inclusion Criteria*** | Eligible patients were 50 to 85 years of age, met the criteria for a diagnosis of probable AD as defined by the National Institute of Neurologic and Communicative Disorders and Stroke–AD and Related Disorders Association,[9](https://aan.m7h.net/content/64/9/1553#ref-9) and had an MRI brain scan supporting the clinical diagnosis of AD. Additional inclusion criteria were a score of 15 to 26 on the MMSE, a Rosen-Modified Hachinski Ischemic score of ≤4, and written, informed consent from the patient and the patient’s caregiver for the original protocol and subsequent amendments. |
| ***Exclusion Criteria*** | Patients were excluded if they had clinically significant neurologic disease, other than AD, that might affect cognition; a major psychiatric disorder, systemic illness, or symptoms that could affect the patient’s ability to complete the study; a Hamilton Psychiatric Rating Scale for Depression score of >12; used anticonvulsant, antiparkinsonian, anticoagulant, narcotic, or immunosuppressive medications within 3 months prior to baseline; used medication with the potential to affect cognition (unless maintained on a stable low to moderate dose regimen for at least 3 months prior to baseline); or used medications for cognitive enhancement other than a stable dosing regimen of an acetylcholinesterase inhibitor (≥6 months). |
| ***Study Design*** | This randomized, placebo-controlled, double-blind, phase IIa clinical trial was conducted at 28 centers in the United States and Europe between September 2001 and December 2002. A total of 372 patients with mild to moderate AD were randomly assigned in a double-blind manner to receive treatment with a suspension of AN1792 225 μg and QS-21 50 μg containing 0.4% polysorbate-80, or normal saline (placebo) in a 4:1 ratio. |
| ***Efficacy Outcomes*** | MMSE; ADAS–Cog; AD Cooperative Study–Clinical Global Impression of Change (ADCS–CGIC), seven-point scale; Disability Assessment for Dementia (DAD); Clinical Dementia Rating (CDR) scale,; and a Neuropsychological Test Battery (NTB). |
| ***Safety Outcomes*** | Adverse events, serious adverse events and death. |

| **Trials** | **Fleisher et al.**  **NCT00244322** |
| --- | --- |
| ***Inclusion Criteria*** | Participants were 50 years or older and diagnosed as having probable AD, as defined by the National Institute of Neurological and Communicative Disorders and Stroke and the Alzheimer Disease and Related Disorders Association criteria. Individuals receiving stable doses of cholinesterase inhibitor drugs or memantine were included. |
| ***Exclusion Criteria*** | Patients were excluded if they had a history of irritable bowel syndrome, chronic diarrhea, peptic ulcer, or gastroesophageal reflux disease; a history of cardiac disease; significant electrocardiographic (ECG) abnormalities; hematologic disorders; hepatic or renal disease; active malignancy within 5 years; or clinically important depressive, neuropsychiatric, cerebrovascular, or respiratory disease. |
| ***Study Design*** | This is a multicenter, randomized, double-blind, placebo-controlled, dose-escalation trial. Participants were randomized to receive LY450139 or placebo using a 2:1 randomization scheme via a telephone-based interactive voice-response system. Patients randomized to the LY450139 groups received 60 mg/d for 2 weeks, then 100 mg/d for the next 6 weeks. At 8 weeks, the treatment arm was randomized again to receive 6 additional weeks of treatment at either 100 or 140 mg/d. |
| ***Efficacy Outcomes*** | At baseline and at weeks 8 and 14, assessments were made of cognition and daily functioning using ADAS-Cog and ADCS-ADL. |
| ***Safety Outcomes*** | Adverse events and serious adverse events. |

| **Trials** | **Lannfelt et al.**  **NCT00471211** |
| --- | --- |
| ***Inclusion Criteria*** | Eligible patients were community dwelling, aged over 55 years, and satisfied the following criteria: had a MMSE score between 20 and 26 points or ADAS-cog score between 10 and 25 points (both scores must have been consistent with early AD); met the National Institute of Neurological and Communicative Disorders and Stroke and the Alzheimer's Disease and Related Disorders Association (NINCDS-ADRDA) criteria; taking a stable dose of acetylcholinesterase inhibitor (donepezil, galantamine, or rivastigmine) for at least 4 months; a modified Hachinski score of 4 points or less; and CT or MRI results consistent with AD. |
| ***Exclusion Criteria*** | The main exclusion criteria were: evidence or history of any other type of dementia or neurological disease, including clinically significant stroke; history of optic neuritis or diagnosis of age-related macular degeneration; abnormal liver function tests (twice the upper limit of normal range); creatinine clearance less than 60% of healthy rate; random blood glucose greater than 50% upper limit of healthy concentration; uncontrolled hypertension at screening; any clinically relevant, unstable haematological or biochemical disorder; WHO international classification of diseases (ICD-10) criteria-based diagnosis of major psychiatric disorder or alcohol or substance abuse; current use of memantine; use of drugs with substantial anticholinergic or antihistaminergic effects; or use of drugs with a narrow therapeutic margin that are metabolised by the cytochrome p450 1A2 isoenzyme. |
| ***Study Design*** | Phase IIa, double-blind, randomized, placebo-controlled trial. Eligible patients with early AD were randomly assigned to receive PBT2 (50 mg or 250 mg) or placebo, orally, once per day, for 12 weeks. |
| ***Efficacy Outcomes*** | The NTB was done at every visit. The ADAS-cog and MMSE were done only at screening and week 12. |
| ***Safety Outcomes*** | Adverse events. |

| **Trials** | **Wilcock et al.**  **(084527)**  **(20365/0001/A 69316)** |
| --- | --- |
| ***Inclusion Criteria*** | English-speaking adults 55 years of age or older who were living in the community were eligible to participate. Patients were required to meet the *Diagnostic and statistical manual of mental disorders*, 4th edition (DSM-IV, text revision) criteria for dementia and the NINCDS-ADRDA criteria for probable AD. The following were additional inclusion criteria at screening: no clinically significant focal intracranial lesion on CT or MRI scans within the previous 12 months; MMSE score of 15–26, inclusive; a modified Hachinski ischaemic score of less than 4; at least 6 years of education or sufficient work history to exclude mental retardation; adequate vision and hearing to participate in study assessments; and an English-speaking caregiver who saw the patient at least 4 days per week and accompanied him or her to clinic visits. |
| ***Exclusion Criteria*** | Patients were excluded if they had any of the following: evidence of epilepsy, focal brain lesion, or head injury with loss of consciousness or immediate confusion after the injury; any major psychiatric disorder (eg, DSM-IV-defined psychosis, major depression, bipolar disorder, or alcohol or substance abuse); history of hypersensitivity to any non-steroidal anti-inflammatory drug (NSAID) or cyclo-oxygenase-2-specific inhibitor; recent history of chronic use of NSAIDs or aspirin (>325 mg per day); history of upper gastrointestinal bleeding that required transfusion or surgery within the previous 3 years; documented evidence of an active gastric or duodenal ulcer within the previous 3 months; history of NSAID-associated ulcers; history of active malignancy except for basal cell carcinoma or squamous cell carcinoma of the skin, or prostate cancer, within the preceding 24 months; a chronic or acute renal, hepatic, or metabolic disorder; recent history of investigational drug use or major surgery; an uncontrolled cardiac condition (New York Heart Association class III or IV); anticoagulant therapy within 12 weeks or therapy with any CYP2C9 inhibitor within 2 weeks; or memantine therapy within 30 days before screening. |
| ***Study Design*** | 31 sites in Canada and the UK enrolled patients from November 3, 2003, to April 24, 2006. Patients were randomised (1:1:1) in blocks of six to receive 12 months’ treatment with 400 mg tarenflurbil twice per day, 800 mg tarenflurbil twice per day, or placebo. |
| ***Efficacy Outcomes*** | Cognition was assessed with the ADAS-cog, including the delayed recall subscale, at baseline (day 1 clinic visit) and at 3-month intervals thereafter until month 24. Functional ability was evaluated with the ADCS-ADL, and global function with the CDR-sb. Global function was also assessed as an exploratory outcome measure with the clinician-interview-based impression of change plus caregiver input (CIBIC+). The ADCS-ADL was done on day 1 and at months 6 and 12, and then at 3-month intervals until month 24; the CDR-sb and CIBIC+ were done at 3-month intervals from day 1 to month 24. The NPI and MMSE were exploratory outcomes. |
| ***Safety Outcomes*** | Adverse events, serious adverse events and death. |

| **Trials** | **Green et al.**  **NCT00105547** |
| --- | --- |
| ***Inclusion Criteria*** | 55 years or older and living in the community, meeting criteria for dementia by the *Diagnostic and Statistical Manual of Mental Disorders* (Fourth Edition) (*DSM-IV*), and having probable AD by National Institute of Neurological and Communicative Disorders and Stroke–Alzheimer's Disease and Related Disorders Association criteria. Additional inclusion criteria at screening included no clinically significant focal intracranial pathology as assessed by CT or MRI within the previous 12 months, a modified Hachinski ischemic score of less than 4, at least 6 years of education or sufficient work history to exclude mental retardation, adequate vision and hearing to participate in study assessments, and a reliable caregiver who saw the patient at least 4 days a week and could accompany the patient to each clinic visit. |
| ***Exclusion Criteria*** | Participants were excluded if they had evidence of epilepsy; focal brain lesion; head injury with loss of consciousness or confusion after the injury; Diagnostic and Statistical Manual of Mental Disorders, 4th Edition, Text Revision (DSM-IV-TR) criteria for any major psychiatric disorder including psychosis, major depression, bipolar disorder, or alcohol or substance abuse; history of upper gastrointestinal tract bleeding requiring surgery, transfusion, or both within 3 years or documented evidence of active gastric or duodenal ulcer disease within 3 months; history or evidence of active malignancy, except for prostate cancer, basal cell carcinoma, or squamous cell carcinoma of the skin within 24 months of entry; a chronic or acute renal, hepatic, or metabolic disorder; any use of AD immunotherapy or recent use of any investigational therapy or major surgery; an uncontrolled cardiac condition (New York Heart Association class III or IV); anticoagulant therapy such as warfarin within 12 weeks of enrollment; use of any CYP2C9 enzyme inhibitor or the CYP2C9 enzyme substrates losartan, phenytoin, tamoxifen, torsemide, and fluvastatin within 2 weeks of enrollment; recent history of chronic use of nonsteroidal anti-inflammatory drugs (NSAIDs) at any dose or aspirin greater than 325 mg/d; or history of hypersensitivity to any NSAIDs including cyclooxygenase 2 (COX-2)–specific inhibitors. Race was determined by self-report and was assessed to evaluate possible drug effect modification. |
| ***Study Design*** | A randomized, double-blind, 2-group parallel study was conducted to compare tarenflurbil with placebo for 18 months involving 133 participating trial sites. Patients were predominantly recruited from dementia clinics and were enrolled from February 21, 2005, through April 30, 2008. |
| ***Efficacy Outcomes*** | Co-primary efficacy outcomes were cognition as assessed by the ADAS-Cog and functional ability as assessed by the ADCS-ADL. A key secondary outcome measure assessed global function with the CDR-sb. Additional secondary outcomes included the MMSE, NPI, quality of life scale (QOL-AD, 13-52 points), Caregiver Burden Inventory (96-point scale). Blood samples were collected and stored for population pharmacokinetic analysis and for apolipoprotein E (*APOE*) genotype testing. |
| ***Safety Outcomes*** | Adverse events, serious adverse events and death. |

| **Trials** | **Salloway et al.**  **NCT00112073** |
| --- | --- |
| ***Inclusion Criteria*** | Eligible patients were aged 50 to 85 years inclusive, met criteria for probable AD, and had an MRI consistent with AD. Additional inclusion criteria were a MMSE score of 16–26 and a Rosen Modified Hachinski Ischemic score ≤4. |
| ***Exclusion Criteria*** | Patients were excluded for clinically significant neurologic disease other than AD; a major psychiatric disorder, history of stroke or seizures, a Hamilton Rating Scale score for Depression >12; current anticonvulsant, antiparkinsonian, anticoagulant, or narcotic medications; recent immunosuppressive or cancer chemotherapy medications; or cognitive enhancers other than acetylcholinesterase inhibitors or memantine at a stable dose for at least 120 days before screening. |
| ***Study Design*** | A total of 234 patients were randomly assigned to receive either IV bapineuzumab or placebo, in an 8:7 ratio, in 1 of 4 sequential dose cohorts (0.15, 0.5, 1.0, or 2.0 mg/kg). Adaptive stratified randomization was used to achieve a balance of baseline acetylcholinesterase inhibitor or memantine use and screening MMSE score (low = 16–21 vs high = 22–26). Patients received study drug as a 1-hour IV infusion every 13 weeks for 6 infusions during the 18-month study. |
| ***Efficacy Outcomes*** | The ADAS-Cog and Disability Assessment for Dementia (DAD) scales were co-primary outcomes. The Neuropsychological Test Battery (NTB) and MMSE (range 0–30) were evaluated at the same intervals as the primary measures, and the CDR-SB was administered every 6 months. |
| ***Safety Outcomes*** | Adverse events, serious adverse events and death. |

| **Trials** | **Aisen et al.** |
| --- | --- |
| ***Inclusion Criteria*** | Participants (men and women ≥ 50 years) had a diagnosis of probable AD (DSM-IV-TR and NINCDS-ADRDA) and a MMSE score between 16 and 26. Laboratory assessments, electrocardiograms (ECG) and CT/MRI results were compatible with probable AD. Patients were required to be on a stable dose of a cholinesterase inhibitor (ChEI) which may have been combined with memantine, for a minimum of four months prior to the screening visit. Stable doses (≥ 1 month prior to screening) of anxiolytics, sedatives, hypnotics, antidepressants, antipsychotics, anticonvulsants, oestrogens, statins, and vitamin E (≤ 2050 IU/day) were allowed. |
| ***Exclusion Criteria*** | Patients with any other causes of dementia were excluded, as were those with a body mass index < 19 or > 28, a life expectancy < 2 years, or a clinically significant and uncontrolled medical disease. The study protocol was approved by the ethics review board of each site. |
| ***Study Design*** | This was a randomized, double-blind, placebo-controlled, parallel-group study conducted at 67 study centres across the United States and Canada. Patients were randomized to tramiprosate 100 mg BID, tramiprosate 150 mg BID, or placebo BID for 78 consecutive weeks, including an 8-week dose escalation phase. Treatment was allocated according to a randomization list issued by an independent biostatistician, using a computer random number generator, and balanced to ensure a ratio of 1 : 1 : 1 across groups. Study medication consisted of modified-release coated tablets, identical in external appearance and containing 50 mg of tramiprosate or placebo. All doses were administered orally as three tablets. |
| ***Efficacy Outcomes*** | Clinical efficacy measures were ADAS-cog, CDR-SB. |
| ***Safety Outcomes*** | Adverse events, serious adverse events and death. |

| **Trials** | **Farlow et al.**  **NCT00329082** |
| --- | --- |
| ***Inclusion Criteria*** | Patients were men or nonfertile women aged ≥50 years with mild-to-moderate probable AD, a Folstein MMSE score of 15 through 26, and a Geriatric Depression Scale score of ≤10 on the staff-administered short form. |
| ***Exclusion Criteria*** | Patients with major or unstable illnesses were excluded. |
| ***Study Design*** | This was a phase 2, parallel-group, double-blind, and randomized (4:1) comparison of solanezumab with placebo in mild-to-moderate AD patients. Fifty-two patients received 12 weekly intravenous infusions of normal saline and/or solanezumab (day 0 through day 77), with additional assessments performed on days 91, 112, 147, 210, and 365, primarily to assess safety. Doses of solanezumab were 100 mg every 4 weeks (Q4W), 100 mg weekly (QW), 400 mg Q4W, or 400 mg QW. |
| ***Efficacy Outcomes*** | ADAS-cog was administered at baseline and days 28, 77, and 112. |
| ***Safety Outcomes*** | N/A |

| **Trials** | **Coric et al.**  **NCT00810147** |
| --- | --- |
| ***Inclusion Criteria*** | Patients met the clinical diagnosis of probable AD based on the National Institute of Neurological and Communicative Disorders and Stroke and the Alzheimer's Disease and Related Disorders Association and *Diagnostic and Statistical Manual of Mental Disorders (Fourth Edition, Text Revision)* criteria with a MMSE score of 16 to 26. Patients had a documented cognitive decline for 6 months or longer, brain computed tomography or magnetic resonance imaging (MRI) within 12 months before baseline that had normal results or demonstrated atrophy consistent with AD, no more than mild to moderate white matter disease, 2 or fewer lacunar infarcts, and a Hachinski Ischemia Scale score of 4 or less. Patients were medically stable and had access to a reliable study partner for 10 or more hours per week. |
| ***Exclusion Criteria*** | Exclusion criteria included the following: a medical condition other than AD that could explain the patient's dementia, previous stroke history, immunocompromised, current diagnosis of peptic ulceration or GI bleeding within the last year, positive fecal immunochemical test result for occult blood in the stool at screening, chronic inflammatory bowel disease, chronic or frequent diarrhea or loose stools, history of GI surgery that resulted in malabsorption, vitamin B12 or folate deficiency, hematologic or solid malignant tumor diagnoses within 5 years, Geriatric Depression Scale score of 6 or higher at screening, or exposure to an investigational agent affecting Aβ levels or function within 12 months before screening. Patients with elevated liver enzyme levels, diabetes mellitus, or a glomerular filtration rate less than 30 mL/min/1.73 m2 were also excluded. Patients treated with approved AD medications had stable treatment for 2 months or longer before screening or were to remain free of such medications. |
| ***Study Design*** | This multicenter, global, randomized, double-blind, placebo-controlled, 5-arm, fixed-dose, parallel-group study was performed in a 24-week treatment period. Patients were randomly assigned in equal proportions across the 5 masked treatment groups: placebo or avagacestat once daily at dosages of 25, 50, 100, or 125 mg. |
| ***Efficacy Outcomes*** | Clinical outcome measures (ADAS-cog, CDR-SB, MMSE and ADCS-ADL) were performed at baseline, week 12, and week 24. |
| ***Safety Outcomes*** | N/A |

| **Trials** | **Dodel et al.**  **NCT00812565** |
| --- | --- |
| ***Inclusion Criteria*** | The inclusion criteria were: probable Alzheimer's disease according to the National Institute of Neurological and Communicative Disorders and Stroke and the Alzheimer's Disease and Related Disorders Association criteria, mini-mental state examination score of 16–26, age 50–85 years at baseline, a modified Hachinski-Rosen score of less than 5, and an MRI scan consistent with Alzheimer's disease. Patients had to have been taking a stable dose of an approved Alzheimer's disease drug for at least 3 months before screening. |
| ***Exclusion Criteria*** | Exclusion criteria were: any suspected cause of dementia other than Alzheimer's disease, history or presence of another significant disease of the CNS, a score of more than 7 on the geriatric depression scale, present significant psychiatric disorder, insulin-dependent diabetes mellitus, uncontrolled hypertension, severe liver or kidney disease, history of thromboembolic events, or history of hypersensitivity to blood-derived or plasma-derived products or intravenous immunoglobulin in the previous 6 months. |
| ***Study Design*** | We did this double-blind, block-randomised, placebo-controlled, parallel group, multicentre trial at 12 sites. |
| ***Efficacy Outcomes*** | Clinical efficacy measures were ADAS-Cog, CDR-SB, MMSE, and ADCS-ADL. |
| ***Safety Outcomes*** | Adverse events, serious adverse events and death. |

| **Trials** | **Doody et al.**  **NCT00594568** |
| --- | --- |
| ***Inclusion Criteria*** | Meets criteria for mild to moderate Alzheimer's disease (AD) with MMSE score of 16-26; Modified Hachinski Ischemia Scale score of less than or equal to 4; Geriatric Depression Scale score of less than or equal to 6; A magnetic resonance imaging (MRI) or computerized tomography (CT) scan in the last 2 years with no findings inconsistent with a diagnosis of AD; If female, must be without menstruation for at least 12 consecutive months or have had both ovaries removed |
| ***Exclusion Criteria*** | Is not capable of swallowing whole oral medication; Has serious or unstable illnesses; Does not have a reliable caregiver; Chronic alcohol or drug abuse within the past 5 years; Has ever had active vaccination for AD |
| ***Study Design*** | Double-blind, placebo-controlled trial in which 1537 patients with probable Alzheimer's disease underwent randomization to receive 100 mg of semagacestat, 140 mg of semagacestat, or placebo daily. |
| ***Efficacy Outcomes*** | Efficacy measures included the ADAS-cog, with higher scores indicating greater cognitive impairment, the ADCS-ADL scale (with higher scores indicating better function), the CDR-SB, the NPI, the Resource Utilization in Dementia Lite (RUD-Lite) scale, the European Quality of Life–5 Dimensions (EQ-5D) scale (proxy version), and the MMSE. |
| ***Safety Outcomes*** | Adverse events, serious adverse events and death. |

| **Trials** | **Doody et al.**  **NCT00905372 and NCT00904683** |
| --- | --- |
| ***Inclusion Criteria*** | Meets criteria for mild to moderate Alzheimer's Disease (AD) with MMSE score of 16 through 26 at screening; Modified Hachinski Ischemia Scale score of less than or equal to 4; Geriatric Depression Scale score of less than or equal to 6; A magnetic resonance imaging (MRI) or computerized tomography (CT) scan in the last 2 years with no findings inconsistent with a diagnosis of AD; If receiving concurrent AD treatment, must be on the medication for at least 4 months at a stable dose for at least 2 months prior to randomization |
| ***Exclusion Criteria*** | Has serious or unstable illness(es); Does not have a reliable caregiver who is in frequent contact with patient (at least 10 hours per week); Meets National Institute of Neurological Disorders and Stroke/Association Internationale pour la Recherche et l'Enseignement en Neurosciences (NINDS/AIREN) criteria for vascular dementia; Does not have good venous access, such that intravenous (IV) drug delivery would be difficult; Has had multiple episodes of head trauma or history within the last 5 years of a serious infectious disease affecting the brain; Has allergies to humanized monoclonal antibodies; Chronic alcohol and/or drug abuse within the past 5 years; Has any contraindications for MRI studies; Requires treatment with another monoclonal antibody |
| ***Study Design*** | In two phase 3, double-blind trials (EXPEDITION 1 and EXPEDITION 2), we randomly assigned 1012 and 1040 patients, respectively, with mild-to-moderate Alzheimer's disease to receive placebo or solanezumab (administered intravenously at a dose of 400 mg) every 4 weeks for 18 months. |
| ***Efficacy Outcomes*** | Efficacy measures included the ADAS-cog, with higher scores indicating greater cognitive impairment, the ADCS-ADL scale (with higher scores indicating better function), the CDR-SB, the NPI, the Resource Utilization in Dementia Lite (RUD-Lite) scale, the European Quality of Life–5 Dimensions (EQ-5D) scale (proxy version), and the MMSE. |
| ***Safety Outcomes*** | Death. |

| **Trials** | **Salloway et al.**  **NCT00574132 and NCT00575055** |
| --- | --- |
| ***Inclusion Criteria*** | Eligible patients were 50 to 88 years of age, met the criteria for probable Alzheimer's disease of the National Institute of Neurological and Communicative Disorders and Stroke and the Alzheimer's Disease and Related Disorders Association, and had a magnetic resonance imaging (MRI) scan that showed results consistent with Alzheimer's disease, a score on the MMSE of 16 to 26 (with scores ranging from 0 to 30 and higher scores indicating less impairment), and a score on the Hachinski Ischemic scale, as modified by Rosen et al., of 4 or lower (with scores ranging from 0 to 12 and higher scores indicating greater degrees of ischemia). |
| ***Exclusion Criteria*** | Exclusion criteria were neurologic disease other than Alzheimer's disease; a screening brain MRI scan that showed evidence of an abnormality (two or more microhemorrhages, a prior hemorrhage larger than 1 cm3, two or more lacunar infarcts, a prior infarct larger than 1 cm^3^, or space-occupying lesions); a major psychiatric disorder; a history of stroke or seizures; and treatment with cognitive enhancers other than stable doses of acetylcholinesterase inhibitors or memantine. |
| ***Study Design*** | Double-blind, randomized, placebo-controlled, phase 3 trials involving patients with mild-to-moderate Alzheimer's disease — one involving 1121 carriers of the apolipoprotein E (APOE) ε4 allele and the other involving 1331 noncarriers. |
| ***Efficacy Outcomes*** | The primary outcome measures, the ADAS-cog11 and DAD scores, were assessed at baseline, at treatment visits, and at week 78. Other cognitive and functional outcome measures included scores on the Neuropsychological Test Battery (which was scored on a standardized z scale, with higher scores indicating less impairment), the CDR–SB (with scores ranging from 0 to 18 and higher scores indicating greater impairment), the MMSE, and the Dependence Scale (with scores ranging from 0 to 15 and higher scores indicating greater need for assistance). |
| ***Safety Outcomes*** | Adverse events, serious adverse events and death. |

| **Trials** | **Arai et al.**  **NCT00752232 and NCT00959192** |
| --- | --- |
| ***Inclusion Criteria*** | Diagnosis of mild to moderate Alzheimer's Disease; MMSE of 16-26. |
| ***Exclusion Criteria*** | Significant Neurological Disease; Major Psychiatric Disorder; Clinically significant systemic illness. |
| ***Study Design*** | Phase 2a, randomized, third-party, unblinded multiple ascending-dose study. |
| ***Efficacy Outcomes*** | Efficacy measures included the ADAS-cog and MMSE. |
| ***Safety Outcomes*** | Adverse events, serious adverse events and death. |

| **Trials** | **Farlow et al.**  **NCT00733863 and NCT00795418** |
| --- | --- |
| ***Inclusion Criteria*** | Patients were male or female (not of childbearing potential), aged 40 to 85 years and had a diagnosis of AD according to the Diagnostic and Statistical Manual of Mental Disorders, Fourth Edition and probable AD according to the Work Group of the National Institute of Neurological and Communicative Disorders and Stroke and the Alzheimer’s Disease and Related Disorders Association. At the time of study entry, patients were required to have mild AD (MMSE score 20 to 26), be untreated or on a stable dose (previous 6 weeks) of ChEI or other AD treatment, and to be in at least daily contact with a primary caregiver. |
| ***Exclusion Criteria*** | Exclusion criteria included other medical or neurological conditions contributing significantly to the patient’s dementia; a history (past 2 years) or current diagnosis of central nervous system (CNS) inflammation indicative of meningoencephalitis or another immune disorder; a history of clinical stroke, intracranial hemorrhage or aneurysm; current diagnosis of significant cerebrovascular disease; and evidence of more than two cerebral microhemorrhages identified by a magnetic resonance imaging (MRI) central reader. |
| ***Study Design*** | Two phase IIa, 52-week, multicenter, randomized, double-blind, placebo-controlled core studies (2201; 2202) were conducted in patients with mild Alzheimer’s disease (AD) aged 40 to 85 years. Patients were randomized to receive 150μg CAD106 or placebo given as three subcutaneous (2201) or subcutaneous/intramuscular (2202) injections. |
| ***Efficacy Outcomes*** | The clinical assessments ADAS-Cog. |
| ***Safety Outcomes*** | Adverse events, serious adverse events and death. |

| **Trials** | **Coric et al.**  **NCT00890890** |
| --- | --- |
| ***Inclusion Criteria*** | Randomized patients with PDAD met the following criteria: (1) clinical symptoms of MCI but not DSM-IV-TR criteria for dementia and (2) CSF biomarker re- sults consistent with the presence of amyloidopathy (Aβ42 level of <200 pg/mL or total tau to Aβ42 ratio of ≥0.39). Clinical MCI criteria required a subjective memory problem verified by a study partner, as well as demonstration of abnormal memory functioning as documented by at least 1 of the 4 following criteria: (1) scoring below the educational level–adjusted cutoff (1.5 SDs below the mean) on the Logical Memory II subscale from the Wechsler Memory Scale–Revised, (2) Free and Cued Selective Reminding Test Total Recall score of 39 or less, (3) Free and Cued Selective Reminding Test Free Recall score of 24 or less, or (4) Free and Cued Selective Reminding Test Delayed Free Recall score of 8 or less. Other inclusion criteria included MMSE score between 24 and 30, and Clinical Dementia Rating global score of 0.5 with a memory box score of 0.5 or less. In addition, screening magnetic resonance imaging (MRI) had to meet all of the following criteria: (1) provide a qualitative assessment showing either a normal MRI commensurate with age or atrophy consistent with an AD diagnosis, (2) reveal no focal asymmetric lobar atrophy or other findings suggesting that the primary cause of dementia was better attributed to a cause other than AD, (3) reveal no more than mild to moderate white matter disease, (4) reveal no more than 4 cerebral microhemorrhages, and (5) reveal no current or prior evidence of macrohemorrhages (>10 mm). |
| ***Exclusion Criteria*** | Exclusion criteria were as follows: (1) presence of a condition other than AD to explain the patient’s cognitive symptoms, (2) previous stroke, (3) positive fecal test for occult blood at screening, (4) chronic inflammatory bowel disease, (5) frequent diarrhea or loose stools, (6) vitamin B12 or folate deficiency, (7) Geriatric Depression Scale24 score of 6 or higher at screening, and (8) exposure to an investigational agent related to Aβ modulation within 12 months before screening. Patients who received stable doses of approved AD medications for at least 2 months prior to screening or who remained free of such medications throughout the trial were also excluded. |
| ***Study Design*** | The treatment period of this multicenter, global, randomized, double-blind, 2-arm, placebo-controlled, parallel- group, randomized clinical trial was planned to extend until at least 2 years after the last patient was randomized. |
| ***Efficacy Outcomes*** | Clinical outcome measures, including the ADAS–cog, CDR-SB, and ADCS-ADL were performed at screening, baseline, and approximately every 12 weeks thereafter. Other outcome measures (MMSE and Free and Cued Selective Reminding Test) were performed at screening and/or baseline and approximately every 24 weeks thereafter. |
| ***Safety Outcomes*** | Adverse events and serious adverse events. |

| **Trials** | **Dyck et al.**  **NCT01227564** |
| --- | --- |
| ***Inclusion Criteria*** | Concern about a change in cognition expressed by the subject or by an informant that knows the subject well; MMSE score ≥ 25; Global Clinical Dementia Rating = 0.5; General cognition and functional performance sufficiently preserved such that a diagnosis of Alzheimer's dementia cannot not be made by the site physician at the time of screening; Amyloid burden detected on screening brain PET scan. |
| ***Exclusion Criteria*** | Significant neurological disease other than early Alzheimer's disease; Major psychiatric disorder or symptom; Contraindication to undergo brain MRI; Unstable medical conditions. |
| ***Study Design*** | Phase 2, multicenter, randomized, third-party unblinded, placebo-controlled, parallel-group trial |
| ***Efficacy Outcomes*** | Clinical outcome measures, including the ADAS–cog, CDR-SB, MMSE and ADCS-ADL. |
| ***Safety Outcomes*** | Adverse events and serious adverse events. |

| **Trials** | **Delnomdedieu et al.**  **NCT01193608** |
| --- | --- |
| ***Inclusion Criteria*** | Diagnosis of probable Alzheimer's Disease with MMSE score of 16-26, and brain MRI consistent with the diagnosis of Alzheimer's Disease; Concurrent use of cholinesterase inhibitor or memantine allowed, if stable; Caregiver will participate and be able to attend clinic visits with patient |
| ***Exclusion Criteria*** | Significant neurological disease other than Alzheimer's Disease; Major psychiatric disorder; Contraindication to undergo brain MRI (e.g., pacemaker, CSF shunt, or foreign metal objects in the body); Women of childbearing potential. |
| ***Study Design*** | The FIH study was a randomized, double-blind, placebo-controlled, safety, tolerability, and pharmacokinetic (PK) adaptive design, dose-escalation study of AAB-003 in male and female subjects with mild to moderate AD. Five AAB-003 dose levels were studied in escalating fashion: 0.5, 1, 2, 4 and 8 mg/kg. |
| ***Efficacy Outcomes*** | Efficacy of AAB-003 was evaluated using the ADAS-Cog, Disability Assessment for Dementia (DAD), NPI, Clinical Dementia Rating (CDR) and MMSE. |
| ***Safety Outcomes*** | Adverse events, serious adverse events and death. |

| **Trials** | **Pasquier et al.**  **NCT00479557 and NCT00498602** |
| --- | --- |
| ***Inclusion Criteria*** | Diagnosis of probable Alzheimer's Disease with MMSE score of 16-26 (except Germany: 21-26); Brain MRI consistent with Alzheimer Disease; Concurent use of Chloniesterase inhibitor or memantine allowed if stable. |
| ***Exclusion Criteria*** | Significant Neurological Disease other than Alzheimer's disease; Major psychiatric disorder; Contraindication to undergo brain MRI; Clinically significant systemic illness |
| ***Study Design*** | Two phase 2a, multicenter, randomized, third-party–unblinded, adjuvant- and placebo-controlled, multiple ascending–dose, safety, tolerability, and immunogenicity studies of ACC-001 with and without QS-21 adjuvant enrolled patients with mild-to-moderate AD dementia at 17 sites in the European Union and 13 sites in the United States beginning in May 2007, with the last subject visit in February 2013 |
| ***Efficacy Outcomes*** | Clinical outcome measures, including the ADAS-Cog, CDR-SB, MMSE, and NPI. |
| ***Safety Outcomes*** | Adverse events, serious adverse events and death. |

| **Trials** | **Vandenberghe et al.**  **NCT00667810 and NCT00676143** |
| --- | --- |
| ***Inclusion Criteria*** | Eligible patients were 50 to 88 years of age, met the criteria for probable Alzheimer's disease of the National Institute of Neurological and Communicative Disorders and Stroke and the Alzheimer's Disease and Related Disorders Association, and had a magnetic resonance imaging (MRI) scan that showed results consistent with Alzheimer's disease, a score on the MMSE of 16 to 26 (with scores ranging from 0 to 30 and higher scores indicating less impairment), and a score on the Hachinski Ischemic scale, as modified by Rosen et al., of 4 or lower (with scores ranging from 0 to 12 and higher scores indicating greater degrees of ischemia). |
| ***Exclusion Criteria*** | Exclusion criteria were neurologic disease other than Alzheimer's disease; a screening brain MRI scan that showed evidence of an abnormality (two or more microhemorrhages, a prior hemorrhage larger than 1 cm3, two or more lacunar infarcts, a prior infarct larger than 1 cm3, or space-occupying lesions); a major psychiatric disorder; a history of stroke or seizures; and treatment with cognitive enhancers other than stable doses of acetylcholinesterase inhibitors or memantine. |
| ***Study Design*** | Studies 3000 and 3001 were multicenter, randomized, double-blind, placebo-controlled, 18-month clinical trials in which investigators evaluated the efficacy and safety of bapineuzumab 0.5 mg/kg versus placebo (ratio 3:2) in ApoE ε4 carriers and bapineuzumab 0.5 mg/kg, 1.0 mg/kg, or placebo (ratio 3:3:4) in ApoE ε4 noncarriers. |
| ***Efficacy Outcomes*** | Efficacy endpoints were change from baseline to week 78 in ADAS-Cog score and Disability Assessment for Dementia (DAD) total score. Change from baseline to week 78 in Dependence Scale, CDR-SOB, and Neuropsychological Test Battery (NTB) total Z-scores were additional endpoints. |
| ***Safety Outcomes*** | Adverse events and serious adverse events. |

| **Trials** | **Vandenberghe et al.**  **NCT 01097096** |
| --- | --- |
| ***Inclusion Criteria*** | Eligible patients aged <85 years with mild AD (Mini–Mental State Examination [MMSE] score: 20–26) diagnosed according to the Diagnostic and Statistical Manual of Mental Disorders version IV and National Institute of Neurological and Communicative Disorders and Stroke and the Alzheimer's Disease and Related Disorders Association criteria were included. |
| ***Exclusion Criteria*** | Exclusion criteria included a history in the past 2 years of central nervous system (CNS) inflammation and on screening, brain magnetic resonance imaging (MRI) of more than one or two microhemorrhages (depending on the field strength). |
| ***Study Design*** | This was a phase 2, 90-week, randomized, double-blind, placebo-controlled, multicenter study. Patients were centrally randomized into two semi-overlapping cohorts across nine treatment groups with an overall ratio of 7:1 CAD106 versus placebo |
| ***Efficacy Outcomes*** | Changes in clinical status were assessed using Alzheimer's Disease Assessment Scale–Cognitive Subscale (including delayed recall), Alzheimer's disease Cooperative Study–Activities of Daily Living, Clinical Dementia Rating scale, MMSE, and Neuropsychiatric Inventory Questionnaire. |
| ***Safety Outcomes*** | Adverse events, serious adverse events and death. |

| **Trials** | **Landen et al.** |
| --- | --- |
| ***Inclusion Criteria*** | Subjects were men and women aged ≥50 years with a MMSE score of 16 to 26 and otherwise healthy. The diagnosis of probable AD was consistent with criteria from the Nation Institute of Neurological and Communicative Disorders and Stroke and the Alzheimer's Disease and Related Disorders Association and the *Diagnostic and Statistical Manual of Mental Disorders, Fourth Edition - Text Revision*. Subjects had a Rosen-Modified Hachinski Ischemia Score ≤4 to minimize the potential confounding effect of vascular dementia. |
| ***Exclusion Criteria*** | Subjects were ineligible if they had a personal or family history of presenilin mutations or other contributors to dementia; diagnosis or history of cerebrovascular disease, severe carotid stenosis, cerebral hemorrhage, intracranial tumor, subarachnoid hemorrhage, or subdural hematoma. Subjects were excluded if brain magnetic resonance imaging (MRI) showed cortical infarct, >2 microhemorrhages (ARIA-H), strategically located subcortical gray matter infarct (e.g., hippocampus, thalamus, and caudate head), or multiple (≥2) white matter lacunes. Subjects were on a stable dose of background acetylcholinesterase inhibitor and/or memantine. |
| ***Study Design*** | This was a Phase II, double-blind, randomized, placebo-controlled study conducted at three centers in Sweden. Subjects were enrolled in cohort Q (quarterly) or cohort M (monthly). Cohort Q was fully enrolled before enrollment in Cohort M. Subjects in cohort Q received ponezumab 10 mg/kg or placebo quarterly (every 3 months). Cohort M received an initial single loading dose of ponezumab 10 mg/kg or placebo, followed by monthly doses of ponezumab 7.5 mg/kg or placebo, respectively. |
| ***Efficacy Outcomes*** | Cognitive function was assessed using the ADAS-Cog at baseline and months 3, 6, 9, 13, and 18. MMSE was performed at screening, baseline, and at month 13. Functional status was measured using the 100-point Disability Assessment for Dementia (DAD) scale at baseline and months 6, 13, and 18. |
| ***Safety Outcomes*** | Adverse events, serious adverse events and death. |

| **Trials** | **Ostrowitzki et al.**  **NCT01224106** |
| --- | --- |
| ***Inclusion Criteria*** | Patients in SCarlet RoAD were 50–85 years of age and met International Working Group criteria for prodromal AD, with biomarker evidence of amyloid pathology and largely preserved functional abilities such that a diagnosis of dementia could not be made. Clinical status was documented by an MMSE score ≥ 24, a CDR global score of 0.5 with an accompanying memory box score of 0.5 or 1.0, abnormal memory function based on an FCSRT score of either < 17 free recall, < 40 total recall, or < 20 free recall, and < 42 total recall; a score ≤ 4 on the modified Hachinski Ischemic Scale; and absence of depression documented by a score ≤ 6 on the Geriatric Depression Scale (GDS). Evidence of amyloid pathology was required as determined by a CSF Aβ1–42 level ≤ 600 ng/L. |
| ***Exclusion Criteria*** | Exclusion criteria were neurological disease other than AD, abnormal brain MRI at screening (including three or more microhemorrhages [1.5 T], two or more lacunar infarcts, extensive/confluent deep white matter lesions, or any space occupying lesions), a major psychiatric disorder, and a history of stroke or any clinically unstable medical illness. Symptomatic treatment with memantine or acetylcholinesterase inhibitors was not permitted at any time during the study; patients requiring such antidementia therapy were to be discontinued from the study. |
| ***Study Design*** | SCarlet RoAD was a phase III, multicenter, randomized, double-blind, placebo-controlled, parallel-group, 2-year study of gantenerumab in prodromal AD |
| ***Efficacy Outcomes*** | The primary endpoint was the change from baseline in CDR-SB at week 104. Secondary endpoints included changes in cognition, behavior, and daily function over 104 weeks. Cognition was assessed using the ADAS-Cog, the MMSE, a computerized cognitive battery (Cambridge Neuropsychological Test Automated Battery [CANTAB]), and the FCSRT. Behavior and daily functioning were assessed using the Neuropsychiatric Inventory Questionnaire (NPI-Q) and the Functional Activities Questionnaire (FAQ), respectively. |
| ***Safety Outcomes*** | Adverse events, serious adverse events and death. |

| **Trials** | **Relkin et al.**  **NCT00818662** |
| --- | --- |
| ***Inclusion Criteria*** | Eligible participants were community-dwelling, medically stable adults, aged 50–89 years, clinically diagnosed with probable AD dementia according to the criteria of the National Institute of Neurologic and Communicative Disorders and Stroke—Alzheimer's Disease and Related Disorders Association[15](https://aan.m7h.net/content/88/18/1768.full#ref-15) of mild to moderate severity, with an available study partner to report their status throughout the trial. Participants had MMSE scores between 16 and 26 inclusive. Treatment with a cholinesterase inhibitor (donepezil or rivastigmine or galantamine), an NMDA antagonist (memantine), or both was allowed if dosing was stable for at least 12 weeks prior to screening. MRI brain scan consistent with AD was required for inclusion. |
| ***Exclusion Criteria*** | Participants with untreated hypercholesterolemia, immunoglobulin A deficiency, or renal insufficiency were excluded owing to increased risks of IVIg treatment. participants with contraindications to MRI were excluded, as were those found to have 2 or more cerebral microhemorrhages, brain infarctions greater than 1 cm3, or space-occupying brain lesions. Participants were excluded if they showed evidence of other causes of dementia, unstable general medical conditions, or untreated major psychiatric disorders, or were taking other investigational AD medications. |
| ***Study Design*** | This was a randomized, parallel-group, placebo-controlled trial in which the blinded study medication was administered to individuals taking stable doses of approved AD medications. The study was conducted at 41 sites in the United States and 4 sites in Canada between December 2008 and February 2013. |
| ***Efficacy Outcomes*** | The ADAS-Cog was assessed at baseline and every 3 months through month 18. The ADCS-ADL was administered at baseline, 9 months, and 18 months. Additional cognitive measures including the modified MMSE and select neuropsychological tests were administered at baseline and 9 and 18 months. The NPI, the Quality of Life in Alzheimer's Disease Scale (QOL-AD), and the Alzheimer's Disease Cooperative Study Clinician's Global Impression of Change (ADCS-CGIC) scales were also assessed. |
| ***Safety Outcomes*** | Adverse events, serious adverse events and death. |

| **Trials** | **Cummings et al.**  **NCT01343966** |
| --- | --- |
| ***Inclusion Criteria*** | Eligible patients were 50 to 80 years old, met the criteria for mild to moderate probable AD according to the National Institute of Neurologic and Communicative Disorders and Stroke—Alzheimer's Disease and Related Disorders Association criteria and had a MMSE score of 18 to 26 points. Additional inclusion criteria were a Geriatric Depression Scale score of <6, a CDR-SB score of ≥0.5, and an ADAS-Cog Delayed Word Recall score of ≥5. Treatment with approved AD drugs such as acetylcholinesterase inhibitors or memantine was permitted if initiated ≥3 months and stabilized ≥2 months before randomization. |
| ***Exclusion Criteria*** | Severe or unstable medical condition that, in the opinion of the investigator or Sponsor, would interfere with the patient's ability to complete the study assessments or would require the equivalent of institutional or hospital care; History or presence of clinically evident vascular disease potentially affecting the brain; History of severe, clinically significant (persistent neurologic deficit or structural brain damage) central nervous system trauma; Hospitalization within 4 weeks prior to screening; Previous treatment with MABT5102A or any other therapeutic that targets A beta; Treatment with any biologic therapy within 5 half-lives or 3 months prior to screening, whichever is longer, with the exception of routinely recommended vaccinations, which are allowed |
| ***Study Design*** | The study was conducted in 2 overlapping parts. Patients were randomly assigned in a 2:1 ratio to receive crenezumab 300 mg SC every 2 weeks (the low-dose cohort) or placebo in part 1 and to crenezumab 15 mg/kg IV every 4 weeks (the high-dose cohort) or placebo in part 2. |
| ***Efficacy Outcomes*** | The primary efficacy outcome measures were changes in the ADAS-Cog and CDR-SB scores from baseline to week 73. The secondary efficacy outcome measure, the ADCS-ADL score, was analyzed in the same manner as the primary efficacy outcome measures. |
| ***Safety Outcomes*** | Adverse events, serious adverse events and death. |

| **Trials** | **Egan et al.**  **NCT01739348** |
| --- | --- |
| ***Inclusion Criteria*** | Diagnosis of probable AD based on both a) the NINCDS-ADRDA criteria and b) the DSM-IV-TR criteria for AD; AD is of mild to moderate severity; Clear history of cognitive and functional decline over at least one year that is either a) documented in medical records or b) documented by history from an informant who knows the subject well; Able to read at a 6th-grade level or equivalent, and must have a history of academic achievement and/or employment sufficient to exclude mental retardation; If a participant is receiving an acetylcholinesterase inhibitor, memantine, medical food/supplement (e.g., vitamin E) and/or herbal medications for AD, the dose must have been stable for at least three months before Screening, and the participant must be willing to remain on the same dose for the duration of the trial. Participants may need to be on AD treatments in accordance with local requirements; Participants must have a reliable and competent trial partner/caregiver who must have a close relationship with the subject |
| ***Exclusion Criteria*** | History of stroke; Evidence of a neurological disorder other than the disease being studied (i.e., probable AD); History of seizures or epilepsy within the last 5 years before Screening; Evidence of a clinically relevant or unstable psychiatric disorder, excluding major depression in remission; Participant is at imminent risk of self-harm or of harm to others; History of alcoholism or drug dependency/abuse within the last 5 years before Screening; Participant does not have a magnetic resonance imaging (MRI) scan obtained within 12 months of Screening and is unwilling or not eligible to undergo an MRI scan at the Screening Visit. With Sponsor approval, a head computed tomography (CT) scan may be substituted for MRI scan to evaluate eligibility; History of hepatitis or liver disease that has been active within the six months prior to Screening Visit; Recent or ongoing, uncontrolled, clinically significant medical condition within 3 months of the Screening Visit (e.g., diabetes, hypertension, thyroid or endocrine disease, congestive heart failure, angina, cardiac or gastrointestinal disease, dialysis, or abnormal renal function) other than the condition being studied such that participation in the trial would pose a significant medical risk to the subject. Controlled co-morbid conditions are not exclusionary if stable within three months of the Screening Visit; History or current evidence of long QT syndrome, corrected QT (QTc) interval ≥470 milliseconds (for male subjects) or ≥480 milliseconds (for female subjects), or torsades de pointes; History of malignancy occurring within the five years before Screening, except for adequately treated basal cell or squamous cell skin cancer, in situ cervical cancer, or localized prostate carcinoma; or malignancy which has been treated with potentially curative therapy with no evidence of recurrence for ≥3 year post-therapy; Clinically significant vitamin B12 or folate deficiency in the six months before Screening Visit; Use of any investigational drugs within 30 days (or longer depending on drug) before Screening or participation in studies involving repeated cognitive testing within 30 days before Screening. Participation in an observational study, such as those involving annual cognitive assessments and/or neuroimaging, may be allowed if approved by Sponsor; History of a hypersensitivity reaction to more than three drugs; Has tested positive for human immunodeficiency virus (HIV); Close family member (including the caregiver, the spouse or any children) who is among the personnel of the investigational or sponsor staff directly involved with this trial |
| ***Study Design*** | The trial was conducted at 238 centers in 21 countries from November 2012 through April 2017. The trial consisted of a randomized, double-blind, placebo-controlled, parallel-group, 78-week trial period. In the phase 2 lead-in safety period, patients were randomly assigned to receive, once daily, one of three oral dose levels of verubecestat (12 mg, 40 mg, or 60 mg) or placebo. |
| ***Efficacy Outcomes*** | Evaluation of clinical efficacy included assessment of cognition according to the cognitive subscale of the ADAS-cog (with higher scores indicating worse dementia) and according to the MMSE, assessment of dementia according to the CDR-SB (with higher scores indicating worse dementia), and assessment of daily function according to the 23-item version of the ADCS-ADL (with lower scores indicating worse function). Assessment of neuropsychiatric symptoms was performed with the use of the NPI (with higher scores indicating more severe symptoms). |
| ***Safety Outcomes*** | Adverse events, serious adverse events and death. |

| **Trials** | **Honig et al.**  **NCT01900665** |
| --- | --- |
| ***Inclusion Criteria*** | Male and female patients, 55 to 90 years of age, who met the diagnostic criteria for probable Alzheimer’s disease according to the National Institute of Neurological and Communicative Disorders and Stroke and the Alzheimer’s Disease and Related Disorders Association. |
| ***Exclusion Criteria*** | Does not have a reliable caregiver who is in frequent contact with the participant (defined as at least 10 hours per week), will accompany the participant to the office and/or be available by telephone at designated times, and will monitor administration of prescribed medications; Meets National Institute of Neurological Disorders and Stroke/Association Internationale pour la Recherche et l'Enseignement en Neurosciences (NINDS/AIREN) criteria for vascular dementia; Has current serious or unstable illnesses including cardiovascular, hepatic, renal, gastroenterologic, respiratory, endocrinologic, neurologic (other than AD), psychiatric, immunologic, or hematologic disease and other conditions that, in the investigator's opinion, could interfere with the analyses of safety and efficacy in this study; or has a life expectancy of <2 years; Has had a history within the last 5 years of a serious infectious disease affecting the brain or head trauma resulting in protracted loss of consciousness; Has a history within the last 5 years of a primary or recurrent malignant disease with the exception of resected cutaneous squamous cell carcinoma in situ, basal cell carcinoma, cervical carcinoma in situ, or in situ prostate cancer with a normal prostate-specific antigen posttreatment; Has a known history of human immunodeficiency virus (HIV), clinically significant multiple or severe drug allergies, or severe posttreatment hypersensitivity reactions; Has received acetylcholinesterase inhibitor (AChEIs), memantine and/or other AD therapy for less than 4 months or has less than 2 months of stable therapy on these treatments; Has received medications that affect the central nervous system (CNS), except treatments for AD, for less than 4 weeks; Has a history of chronic alcohol or drug abuse/dependence within the past 5 years; Has a Visit 1 MRI with results showing >4 Amyloid-related Imaging Abnormality (ARIA), -hemorrhage /hemosiderin deposition (ARIA-H) or presence of ARIA-E (edema/effusions) |
| ***Study Design*** | Patients were randomly assigned in double-blind fashion to receive intravenous infusions of either solanezumab at a dose of 400 mg or placebo every 4 weeks for 76 weeks. Patients who completed the double-blind period could participate in an optional 24-month open-label period. |
| ***Efficacy Outcomes*** | The primary efficacy measure was the change from baseline to 80 weeks in the score on the ADAS-cog (with higher scores indicating greater cognitive impairment). Key secondary efficacy measures included scores on the following assessments: the MMSE; the ADCS-ADL (with lower scores indicating greater functional impairment); the ADCS instrumental subscale (ADCS-iADL), which assesses complex activities such as using public transportation, managing finances, or shopping (scores range from 0 to 56, with lower scores indicating greater functional loss); the CDR-SB (with higher scores indicating greater impairment); the FAQ; and the Integrated Alzheimer’s Disease Rating Scale (iADRS; scores range from 0 to 146, with lower scores indicating worse performance). |
| ***Safety Outcomes*** | Adverse events, serious adverse events and death. |

| **Trials** | **Salloway et al.**  **NCT01397578** |
| --- | --- |
| ***Inclusion Criteria*** | Patients were eligible to participate if they were 50–80 years of age, met the criteria for mild-to-moderate probable AD according to the National Institute of Neurologic and Communicative Disorders and Stroke/Alzheimer’s Disease and Related Disorders Association criteria, with MMSE score of 18–26 points at the time of screening. Additional inclusion criteria were a Geriatric Depression Scale (GDS-15) score of < 6, a CDR-SB score of ≥ 0.5, and an ADAS-Cog Delayed Word Recall score of ≥ 5. Patients were required to have evidence of elevated amyloid burden consistent with a diagnosis of AD indicating moderate-to-frequent neuritic plaques (Aβ-positive) as assessed by a central expert blinded visual reading of the screening florbetapir PET scan. Treatment with approved AD drugs such as acetylcholinesterase inhibitors (AChEIs) or memantine initiated ≥ 3 months and stabilized ≥ 2 months prior to randomization was permitted. |
| ***Exclusion Criteria*** | Severe or unstable medical condition that, in the opinion of the investigator or Sponsor, would interfere with the patient's ability to complete the study assessments or would require the equivalent of institutional or hospital care; History or presence of clinically evident vascular disease potentially affecting the brain (e.g., stroke, clinically significant carotid or vertebral stenosis or plaque, aortic aneurysm, intracranial aneurysm, cerebral hemorrhage, arteriovenous malformation); History of severe, clinically significant (persistent neurologic deficit or structural brain damage) central nervous system trauma (e.g., cerebral contusion); Hospitalization within 4 weeks prior to screening; Previous treatment with MABT5102A or any other therapeutic that targets Abeta; Treatment with any biologic therapy within 5 half-lives or 3 months prior to screening, whichever is longer, with the exception of routinely recommended vaccinations, which are allowed |
| ***Study Design*** | This study was conducted at 21 sites in the US, one site in Spain, and one site in France. The study was conducted in two parts. In part 1, patients were randomly assigned 2:1 (crenezumab:placebo) to 300 mg subcutaneous (SC) crenezumab every 2 weeks (q2w) or placebo (low-dose SC cohort); in part 2, patients were randomly assigned 2:1 (crenezumab:placebo) to 15 mg/kg intravenous (IV) crenezumab every 4 weeks (q4w) or placebo (high-dose IV cohort). |
| ***Efficacy Outcomes*** | The primary outcome measure was the change from baseline to week 73 in ADAS-Cog and CDR-SB. |
| ***Safety Outcomes*** | Adverse events, serious adverse events and death. |

| **Trials** | **Egan et al.**  **NCT01953601** |
| --- | --- |
| ***Inclusion Criteria*** | Patients were eligible for enrollment in the trial if they were between 50 and 85 years of age and if they did not meet criteria for dementia but had had a subjective decrease in memory for at least 1 year corroborated by an informant. Other eligibility criteria were a score on the Repeatable Battery for the Assessment of Neuropsychological Status Delayed Memory Index of at least 1 SD below the age- and education-appropriate population mean, corresponding to a score of 85 or less (scores range from 40 to 160, with lower scores indicating worse memory), and the presence of brain amyloid as gauged by a radiologist’s visual inspection of amyloid-ligand positron-emission tomography (PET) imaging. All the patients underwent medical and neurologic evaluations, including magnetic resonance imaging (MRI) (or computed tomography if MRI was contraindicated). Other entry criteria included a score of 24 to 30 on the MMSE. Patients could have been receiving an acetylcholinesterase inhibitor, memantine, or both, provided that they had received a stable dose for at least 3 months before screening. The diagnosis of prodromal Alzheimer’s disease was confirmed by independent review. |
| ***Exclusion Criteria*** | History of stroke; Evidence of a clinically relevant neurological disorder other than the disease being studied; History of seizures or epilepsy within the last 5 years; Evidence of a clinically relevant or unstable psychiatric disorder, excluding major depression in remission; Participant is at imminent risk of self-harm or of harm to others; History of alcoholism or drug dependency/abuse within the last 5 years before Screening; Participant does not have a magnetic resonance imaging (MRI) scan obtained within 12 months of Screening and is unwilling or not eligible to undergo an MRI scan at the Screening Visit. With Sponsor approval, a head computed tomography (CT) scan may be substituted for MRI scan to evaluate eligibility; History of hepatitis or liver disease that has been active within the 6 months prior to Screening; Recent or ongoing, uncontrolled, clinically significant medical condition within 3 months of Screening; History of malignancy occurring within the 5 years before Screening, except for adequately treated basal cell or squamous cell skin cancer, in situ cervical cancer, or localized prostate carcinoma; Clinically significant vitamin B12 or folate deficiency in the 6 months before Screening; Use of any investigational drugs or participation in clinical trials within the 30 days before Screening; History of a hypersensitivity reaction to more than three drugs; Has human immunodeficiency virus (HIV) by medical history; Participant is unwilling or has a contraindication to undergo PET scanning including but not limited to claustrophobia, excessive weight or girth; History or current evidence of long QT syndrome, corrected QT (QTc) interval ≥470 milliseconds (for male participants) or ≥480 milliseconds (for female participants), or torsades de pointes; Close family member (including the trial partner, spouse or children) who is among the personnel of the investigational or sponsor staff directly involved with this trial |
| ***Study Design*** | The trial was conducted at 238 centers in 22 countries from November 2013 through April 2018. The trial consisted of a randomized, double-blind, placebo-controlled, parallel group, 104-week trial period. Patients were randomly assigned in a 1:1:1 ratio to receive, once daily, oral verubecestat at a dose of 12 mg, oral verubecestat at a dose of 40 mg, or oral placebo. |
| ***Efficacy Outcomes*** | The primary efficacy outcome was the change from baseline to week 104 in the CDR-SB. There were seven secondary outcomes: the score on the ADCS-ADL. Exploratory outcomes included the change from baseline to week 104 in scores on the ADAS-cog. Other exploratory outcomes included the scores on the MMSE and the NPI. |
| ***Safety Outcomes*** | Adverse events, serious adverse events and death. |

| **Trials** | **Wessels et al.**  **NCT02245737 (AMARANTH) and NCT02783573 (DAYBREAK-ALZ)** |
| --- | --- |
| ***Inclusion Criteria*** | Gradual and progressive change in the participant's memory function over more than 6 months, reported by participant and study partner; MMSE score of 20-30 inclusive at screening; Objective impairment in memory as evaluated by memory test performed at screening; For a diagnosis of mild Alzheimer's Disease (AD), participant meets the National Institute on Aging and the Alzheimer's Association (NIA-AA) criteria for probable AD; For a diagnosis of MCI due to AD, participant meets NIA-AA criteria for MCI due to AD |
| ***Exclusion Criteria*** | Significant neurological disease affecting the central nervous system, other than AD, that may affect cognition or ability to complete the study, including but not limited to, other dementias, serious infection of the brain, Parkinson´s disease, or epilepsy or recurrent seizures; History of clinically evident stroke, or multiple strokes based on history or imaging results; History of clinically important carotid or vertebrobasilar stenosis or plaque; History of multiple concussions with sustained cognitive complaints or objective change in neuropsychological function in the last 5 years; Participants with a current Diagnostic and Statistical Manual of Mental Disorders, Fifth Edition diagnosis of Major Depressive Disorder or any current primary psychiatric diagnosis other than AD if, in the judgment of the investigator, the psychiatric disorder or symptom is likely to confound interpretation of drug effect, affect cognitive assessments, or affect the participant´s ability to complete the study; History of alcohol or drug abuse or dependence (except nicotine dependence) within 2 years before the screening; Within 1 year before the screening or between screening and baseline, any of the following: myocardial infarction; moderate or severe congestive heart failure, New York Heart Association class III or IV; hospitalization for, or symptom of, unstable angina; syncope due to orthostatic hypotension or unexplained syncope; known significant structural heart disease, or hospitalization for arrhythmia; Congenital QT prolongation; History of cancer within the last 5 years, with the exception of non-metastatic basal and/or squamous cell carcinoma of the skin, in situ cervical cancer, non-progressive prostate cancer or other cancers with low-risk of recurrence or spread; Current serious or unstable clinically important systemic illness that, in the judgment of the investigator, is likely to affect cognitive assessment, deteriorate, or affect the participant's safety or ability to complete the study, including hepatic, renal, gastroenterologic, respiratory, cardiovascular, endocrinologic, immunologic, or hematologic disorders |
| ***Study Design*** | AMARANTH was a phase 2/3, multicenter, randomized, 104-week, double-blind, placebo-controlled, global study of lanabecestat in patients with early AD, defined as the continuum of patients with mild cognitive impairment (MCI) attributable to AD and patients diagnosed as having mild AD dementia. DAYBREAK-ALZ was a phase 3, multicenter, randomized, double-blind, global study of lanabecestat in patients with mild AD dementia that included a 78-week placebo-controlled period, followed by a 78-week DS period; during the DS period, all patients receiving placebo were switched to lanabecestat. |
| ***Efficacy Outcomes*** | The primary objective was change from baseline to the end of double-blind, placebo-controlled periods on the ADAS-Cog. Secondary objectives included: ADCS-iADL, FAQ, iADRS, CDR-SB, NPI, and MMSE. |
| ***Safety Outcomes*** | Adverse events, serious adverse events and death. |

| **Trials** | **Lo et al.**  **NCT02791191** |
| --- | --- |
| ***Inclusion Criteria*** | Present with mild AD dementia based on the National Institute on Aging (NIA) and the Alzheimer's Association (AA) disease diagnostic criteria as determined by a qualified clinician approved by the Sponsor or designee; MMSE score of 20 to 26 inclusive at screening visit; Has a florbetapir PET scan consistent with the presence of amyloid pathology at screening. |
| ***Exclusion Criteria*** | Significant neurological disease affecting the central nervous system (CNS), other than AD, that may affect cognition or ability to complete the study, including but not limited to, other dementias, serious infection of the brain, Parkinson's disease, multiple concussions, or epilepsy or recurrent seizures (except febrile childhood seizures); Ocular pathology that significantly limits ability to reliably evaluate vision or the retina; Use of strong inducers of cytochrome P450 3A (CYP3A); Sensitivity to florbetapir or ¹⁸F-AV-1451; Contraindication to MRI or PET or poor venous access for blood draws. |
| ***Study Design*** | Double-blind, randomized, placebo-controlled trial |
| ***Efficacy Outcomes*** | The efficacy outcomes included ADAS-cog, MMSE, and NPI. |
| ***Safety Outcomes*** | Adverse events, serious adverse events and death. |

| **Trials** | **Mintun et al.**  **NCT03367403** |
| --- | --- |
| ***Inclusion Criteria*** | The trial included patients 60 to 85 years of age who had early symptomatic Alzheimer’s disease, defined as prodromal Alzheimer’s disease (the symptomatic predementia phase of Alzheimer’s disease in which mild cognitive impairment is apparent, as defined in the protocol) or mild Alzheimer’s disease with dementia (in which symptoms are sufficiently severe to meet diagnostic criteria for dementia and Alzheimer’s disease), and had a MMSE score of 20 to 28. |
| ***Exclusion Criteria*** | Have a history of long QT syndrome; Have received treatment with a stable dose of an acetylcholinesterase inhibitor (AChEI) and/or memantine for less than 2 months before randomization; Contraindication to MRI. |
| ***Study Design*** | TRAILBLAZER-ALZ is a multicenter, randomized, double-blind, placebo-controlled phase 2 trial that assessed the safety, adverse events, and efficacy of donanemab in patients with early Alzheimer’s disease. |
| ***Efficacy Outcomes*** | The primary outcome was the change from baseline to 76 weeks in the score on the iADRS. The key secondary outcomes were the change from baseline in scores on the CDR-SB, the ADAS-Cog, the ADCS-iADL, and the MMSE. |
| ***Safety Outcomes*** | Adverse events, serious adverse events and death. |

| **Trials** | **Swanson et al.**  **NCT01767311** |
| --- | --- |
| ***Inclusion Criteria*** | Participants comprised 2 subgroups: mild cognitive impairment due to AD or mild AD dementia. All subjects were confirmed amyloid positive via amyloid positron emission tomography (PET) or cerebrospinal fluid (CSF) Aβ1–42 for eligibility. Key inclusion criteria included objective impairment in episodic memory (on Wechsler Memory Scale-IV Logical Memory II [WMS-IV LMII]), MMSE score equal to or greater than 22 at screening and baseline (amended to MMSE 22–28 in EU, except Italy), and naïve to or on stable dose (12 weeks) of approved AD medications. |
| ***Exclusion Criteria*** | Any neurological condition that may be contributing to cognitive impairment above and beyond that caused by the subject's AD; History of transient ischemic attacks (TIA), stroke, or seizures within 12 months of Screening; Any psychiatric diagnosis or symptoms, (e.g., hallucinations, major depression, or delusions) that could interfere with study procedures in the subject; Geriatric Depression Scale (GDS) score ≥8 at Screening; Contraindications to MRI scanning, including cardiac pacemaker/ defibrillator, ferromagnetic metal implants, e,g., in skull and cardiac devices other than those approved as safe for use in MR scanners; Evidence of other clinically significant lesions that could indicate a dementia diagnosis other than AD on brain MRI at Screening, or other significant pathological findings on brain MRI at Screening; A prolonged QT/QTc interval (QTc greater than 450 ms) as demonstrated by a repeated electrocardiogram (ECG); Certain other specified medical conditions; Severe visual or hearing impairment that would prevent the subject from performing psychometric tests accurately |
| ***Study Design*** | Study 201 was an 18-month, multicenter, double-blind, placebo-controlled Bayesian design clinical trial employing response adaptive randomization across placebo and five lecanemab arms (2.5 mg/kg biweekly, 5 mg/kg monthly, 5 mg/kg biweekly, 10 mg/kg monthly, 10 mg/kg biweekly) to assess safety and efficacy in subjects with early Alzheimer’s disease. |
| ***Efficacy Outcomes*** | The primary endpoint was change from baseline at 12 months on the Alzheimer’s Disease Composite Score (ADCOMS). ADAS-Cog, and CDR-SB were collected every 3 months during the study. |
| ***Safety Outcomes*** | Adverse events, serious adverse events and death. |

| **Trials** | **Christopher et al.**  **NCT03887455** |
| --- | --- |
| ***Inclusion Criteria*** | The trial included participants 50 to 90 years of age, with either mild cognitive impairment due to Alzheimer’s disease or mild Alzheimer’s disease–related dementia on the basis of National Institute on Aging–Alzheimer’s Association criteria |
| ***Exclusion Criteria*** | Any neurological condition that may be contributing to cognitive impairment above and beyond that caused by the participant's Alzheimer's disease; History of transient ischemic attacks (TIA), stroke, or seizures within 12 months of Screening; Any psychiatric diagnosis or symptoms that could interfere with study procedures in the participant; Geriatric Depression Scale (GDS) score >=8 at Screening; Contraindications to MRI scanning, including cardiac pacemaker/defibrillator, ferromagnetic metal implants; Evidence of other clinically significant lesions on brain MRI at Screening that could indicate a dementia diagnosis other than Alzheimer's disease; Other significant pathological findings on brain MRI at screening, including but not limited to: more than 4 microhemorrhages (defined as 10 millimeter [mm] or less at the greatest diameter); a single macrohemorrhage >10 mm at greatest diameter; an area of superficial siderosis; evidence of vasogenic edema; evidence of cerebral contusion, encephalomalacia, aneurysms, vascular malformations, or infective lesions; evidence of multiple lacunar infarcts or stroke involving a major vascular territory, severe small vessel, or white matter disease; space occupying lesions; or brain tumors; Any immunological disease which is not adequately controlled, or which requires treatment with immunoglobulins, systemic monoclonal antibodies, systemic immunosuppressants, or plasmapheresis during the study; Participants with a bleeding disorder that is not under adequate control. Participants who are on anticoagulant therapy should have their anticoagulant status optimized and be on a stable dose for 4 weeks before Screening. Participants who are on anticoagulant therapy are not permitted to participate in cerebrospinal fluid (CSF) assessments; Any other medical conditions which are not stably and adequately controlled, or which in the opinion of the investigator(s) could affect the participant's safety or interfere with the study assessments; Participation in a clinical study involving any therapeutic monoclonal antibody, protein derived from a monoclonal antibody, immunoglobulin therapy, or vaccine within 6 months before screening unless it can be documented that the participant was randomized to placebo; Participation in a clinical study involving any anti-amyloid therapies (including any monoclonal antibody therapies and any β-site amyloid precursor protein cleaving enzyme [BACE] inhibitor therapies) unless it can be documented that the participant only received placebo; Participants who have any known prior exposure to lecanemab; Participants who were dosed in a clinical study involving any new chemical entities for AD within 6 months prior to screening unless it can be documented that the participant was in a placebo treatment arm |
| ***Study Design*** | Clarity AD was an 18-month, multicenter, double-blind, placebo-controlled, parallel-group trial involving persons with early Alzheimer’s disease. Eligible participants were randomly assigned in a 1:1 ratio to receive intravenous lecanemab (10 mg per kilogram every 2 weeks) or placebo. |
| ***Efficacy Outcomes*** | The primary efficacy end point was the change in the score on the CDR-SB from baseline at 18 months. Key secondary end points were the change from baseline at 18 months in the following: ADAS-cog14, ADCOMS, and the score on the ADCS-ADL. |
| ***Safety Outcomes*** | Adverse events, serious adverse events and death. |

| **Trials** | **Haeberlein et al.**  **NCT02484547 (EMERGE) and NCT02477800 (ENGAGE)** |
| --- | --- |
| ***Inclusion Criteria*** | Aged 50 to 85 years old, inclusive, at the time of informed consent; Must have at least 6 years of education or work experience to exclude mental deficits other than MCI or mild AD; Must have a positive amyloid PET scan. Previously obtained PET scan (within 12 months of Screening) is permissible for subjects not participating in the amyloid PET sub-study. Previous PET scan images must be submitted to the central imaging vendor to confirm study inclusion criteria are met; Must meet all of the following clinical criteria for MCI due to AD or mild AD according to NIA-AA criteria, and must have: A CDR global score of 0.5; An RBANS score of 85 or lower indicative of objective cognitive impairment (based upon the Delayed Memory Index score); An MMSE score between 24 and 30 (inclusive). |
| ***Exclusion Criteria*** | Any uncontrolled medical or neurological/neurodegenerative condition (other than AD) that, in the opinion of the investigator, might be a contributing cause of the subject’s cognitive impairment; Clinically significant unstable psychiatric illness within 6 months prior to Screening; Transient ischemic attack or stroke or any unexplained loss of consciousness within 1 year prior to Screening; Brain MRI performed at Screening (per centrally read MRI) that shows evidence of any of the following: Acute or sub-acute hemorrhage; Prior macrohemorrhage (defined as >1 cm in diameter on T2* sequence) or prior subarachnoid hemorrhage unless it can be documented that the finding is not due to an underlying structural or vascular abnormality (i.e., finding does not suggest subject is at risk of recurrent hemorrhage); Greater than 4 microhemorrhages (defined as £ 1 cm in diameter on T2* sequence); Cortical infarct (defined as >1.5 cm in diameter; irrespective of anatomic location); >1 lacunar infarct (defined as £1.5 cm in diameter); History of bleeding disorder or predisposing conditions, blood clotting or clinically significant abnormal results on coagulation profile at Screening, as determined by the investigator. |
| ***Study Design*** | EMERGE and ENGAGE were randomized, double-blind, placebo-controlled trials conducted at 348 sites in 20 countries. The number of patients enrolled in ENGAGE remained ahead of EMERGE throughout the study enrollment period. Patients were randomized (1:1:1) to receive lowdose aducanumab, high-dose aducanumab, or placebo via intravenous infusion following dilution into saline every 4 weeks over 76 weeks. |
| ***Efficacy Outcomes*** | The primary outcome measure was the CDR-SB. Secondary clinical outcome measures also assessed cognitive decline (MMSE and ADAS-Cog) and ability to perform daily activities (ADCS-ADL). The NPI was the lone tertiary efficacy endpoint. |
| ***Safety Outcomes*** | Adverse events, serious adverse events and death. |

| **Trials** | **Ostrowitzki et al.**  **NCT02670083 (CREAD) and NCT03114657 (CREAD 2)** |
| --- | --- |
| ***Inclusion Criteria*** | Individuals aged 50 to 85 years with prodromal or mild AD consistent with National Institute on Aging–Alzheimer Association criteria, increased Aβ burden (confirmed by cerebrospinal fluid [CSF] Aβ42 levels or visually read positive amyloid PET scan), a MMSE score of 22 or higher, a Clinical Dementia Rating Scale–Global Score of 0.5 or 1.0, and abnormal memory function defined by a Free and Cued Selective Reminding Test cueing index score of 0.67 or lower and a free recall score of 27 or lower. |
| ***Exclusion Criteria*** | Participants were excluded if their medical history included other conditions causing neurological deficit; cancer; or cardiovascular, hepatic, immune, or metabolic disorders. Other exclusion criteria included evidence of cerebral infarction or more than 4 microbleeds or areas of leptomeningeal hemosiderosis (ARIA-hemosiderosis) on magnetic resonance imaging (MRI). |
| ***Study Design*** | CREAD and CREAD2 were randomized placebo-controlled phase 3 studies in participants with prodromal to mild AD. Participants were randomized 1:1 to crenezumab (60 mg/kg) or placebo, administered by IV infusion Q4W for up to 100 weeks. |
| ***Efficacy Outcomes*** | The primary efficacy measure was the CDR-SB score measuring decline in 6 clinical domains due to cognitive loss. Secondary efficacy measures included cognition assessed by the ADAS and MMSE, function assessed by the ADCS–ADL. |
| ***Safety Outcomes*** | Adverse events, serious adverse events and death. |

| **Trials** | **NCT00722046** |
| --- | --- |
| ***Inclusion Criteria*** | Males or females of non childbearing potential, age > or = 50; Diagnosis of probable Alzheimer's disease, consistent with criterial from both:National Institute of NINCDS-ADRDA; Diagnostic and Statistical Manual of Mental Disorders (DSM IV); MMSE of 16-26 inclusive; Rosen-Modified Hachinski Ischemia Score of < or = 4. |
| ***Exclusion Criteria*** | Diagnosis or history of other demential or neurodegenerative disorders; Diagnosis or history of clinically significant cerebrovascular disease; Specific findings on magnetic resonance imaging (MRI); cortical infarct, micro hemorrhage, multiple white matter lacunes, extensive white matter abnormalities; History of autoimmune disorders; History of allergic or anaphylactic reactions. |
| ***Study Design*** | Phase II multicenter randomized double-blind placebo-controlled study. |
| ***Efficacy Outcomes*** | Change From Baseline in Alzheimer's Disease Assessment Scale-Cognitive Subscale (ADAS-cog) Score at Month 19; Change From Baseline in Disability Assessment for Dementia (DAD) Score at Month 19. |
| ***Safety Outcomes*** | Adverse events, serious adverse events and death. |

| **Trials** | **NCT01524887** |
| --- | --- |
| ***Inclusion Criteria*** | Males or females of age 50 to 89 years inclusive at the time of screening; Diagnosis of Probable Alzheimer´s Disease (AD) according to NINCDS-ADRDA* 1984 criteria (* National Institute of Neurological and Communicative Disorders and Stroke - Alzheimer's Disease and Related Disorders Association); Dementia of mild to moderate severity (Mini-Mental State Examination [MMSE] 16-26 inclusive at the time of screening); Neuroimaging (computed tomography [CT] or MRI) performed after symptom onset consistent with AD diagnosis; If receiving psychoactive medications (eg, antidepressants other than monoamine oxidase inhibitors [MAOIs] and most tricyclics, antipsychotics, anxiolytics, anticonvulsants, mood stabilizers, etc.), must be on stable doses for at least 6 weeks prior to screening; For women of childbearing potential, the subject must have a negative pregnancy test at screening and must agree to employ adequate contraceptive measures (eg, birth control pills/patches, intrauterine device, or diaphragm or condom [for male partner] with spermicidal jelly or foam) throughout the course of the study; For subjects with a coronary artery stent, the subject must receive documented medical clearance from an interventional cardiologist stating that the subject is not at increased risk for stent occlusion with immunoglobulin treatment; For subjects with an endovascular stent, the subject must receive documented medical clearance from a vascular surgeon stating that the subject is not at increased risk for thromboembolic events with immunoglobulin treatment |
| ***Exclusion Criteria*** | Possible AD by NINCDS-ADRDA criteria or non-Alzheimer dementia; Contraindication to undergoing MRI (eg, pacemaker [with the exception of an MRI-compatible pacemaker], severe claustrophobia, ferromagnetic implants such as a metal plate); Clinically significant congestive heart failure; Current atrial fibrillation of unstable angina (angina at rest) or history of myocardial infarction within the 12 months prior to screening; Uncontrolled hypertension defined as systolic blood pressure > 160 mm Hg and/or diastolic > 100 mm Hg confirmed upon repeated measures; History of thrombosis and/or thromboembolic disease (central or peripheral) within the 12 months prior to screening; Known history of procoagulant abnormalities (eg, factor V Leiden, antiphospholipid syndrome, protein S/protein C deficiency, AT III deficiency); History of intracerebral hemorrhage within the 5 years prior to screening; Evidence on MRI of: greater than 4 microhemorrhages (regardless of their anatomical location or diagnostic characterization as "possible" or "definite"), a single area of superficial siderosis, vasogenic edema, a macrohemorrhage, major stroke, prominent white matter disease with a rating score of 3 on the age-related white matter changes (ARWMC) scale from the European Task Force on ARWMC, or multiple lacunae (defined as more than 2 lacunae that are greater than 0.5 mm in size); Head trauma with loss of consciousness, contusion, or open head injury within the 12 months prior to screening; Uncontrolled seizure disorder as defined by two or more breakthrough seizures per year despite adequate antiepileptic drug (AED) treatment; Modified Hachinski score > 4 at time of screening; Subjects with active malignancy or history of malignancy within 5 years prior to screening with the exception of the following: adequately treated basal cell or squamous cell carcinoma of the skin, carcinoma in situ of the cervix, and stable prostate cancer not requiring treatment; Active autoimmune or neuro-immunologic disorder; Uncontrolled major depression, psychosis, or other major psychiatric disorder(s); Poorly controlled diabetes, defined as glycosylated (or glycated) hemoglobin (HbA1c) ≥ 6.5% at screening; Creatinine clearance < 50% of normal adjusted for age and gender, as calculated according to the Cockcroft-Gault formula, at the time of screening; Known history of untreated vitamin B12 deficiency within 6 months prior to screening, or clinically significant abnormally low vitamin B12 at the time of screening. |
| ***Study Design*** | Phase 3 Randomized, Double-blind, Placebo-Controlled Study |
| ***Efficacy Outcomes*** | The primary efficacy measure included ADAS-cog, NPI and ADCS–ADL. |
| ***Safety Outcomes*** | Adverse events and serious adverse events. |

| **Trials** | **NCT00762411** |
| --- | --- |
| ***Inclusion Criteria*** | Meets criteria for mild to moderate Alzheimer's disease (AD) with Mini-Mental State Examination score of 16 through 26 at visit 1; Modified Hachinski Ischemia Scale score of less than or equal to 4; Geriatric Depression Scale score of less than or equal to 6; A magnetic resonance imaging (MRI) or computerized tomography (CT) scan in the last 2 years with no findings inconsistent with a diagnosis of AD; If female, must be without menstruation for a least 12 consecutive months or have had both ovaries removed. |
| ***Exclusion Criteria*** | Is not capable of swallowing whole oral medication; Has serious or unstable illnesses; Does not have a reliable caregiver; Chronic alcohol and/or drug abuse within the past 5 years; Has ever had a active vaccination for AD. |
| ***Study Design*** | Randomized placebo-controlled, parallel-group study |
| ***Efficacy Outcomes*** | The primary efficacy measure included ADAS-cog and ADCS–ADL. |
| ***Safety Outcomes*** | Adverse events, serious adverse events and death. |

| **Trials** | **NCT01561430** |
| --- | --- |
| ***Inclusion Criteria*** | Meets criteria for MCI due to AD or Mild AD; All participants will be required to undergo assessment via the MMSE scale at screening; Participants with MMSE scores of 20 to 26, inclusive, may be enrolled provided they meet the criteria for mild AD, as follows: Participant meets the National Institute of Neurological and Communicative Disorders and Stroke/Alzheimer's Disease and Related Disorders Association (NINCDS/ADRDA) criteria for probable AD, Clinical Dementia Rating Scale (CDR) score of 0.5 or 1, Positive scan for the presence of amyloid beta; Participants with MMSE of 27 to 30, inclusive, may be enrolled as participants with MCI due to AD provided they meet the following criteria: Gradual and progressive change in memory function as reported by the participant or a caregiver during a period of more than 6 months, Free and Cued Selective Reminding Test with Immediate Recall (FCSRT-IR): free recall ≤22 and total recall ≤46, Absence of dementia, Preservation of functional independence, Exclusion of other potential (vascular, traumatic, or medical) causes of cognitive decline, where possible, Positive scan for the presence of amyloid beta; Women must be postmenopausal; Men are required to use an approved barrier method of contraception if their partners are pregnant, or of childbearing potential and not using approved contraceptive methods |
| ***Exclusion Criteria*** | Participant in another drug or device study; Have a history of frontotemporal dementia, Lewy body disease, vascular dementia, Huntington's disease, Parkinson's disease, progressive supranuclear palsy (PSNP), or other movement disorder; Participants are not on a stable standard of care (acetylcholinesterase inhibitors, memantine) initiated less than 2 months prior to entry or have less than 4 weeks of stable therapy. Note: Stable standard of care is allowed; Have had a serious infectious disease affecting the brain in the past 5 years; Have had a serious or repeat head injury; Have significant retinal impairment or disease; Have had a stroke or other circulation problems that are affecting current health; Have had a seizure; Have major depressive disorder and are not on a stable dose of medication. Participants who no longer meet the Diagnostic and Statistical Manual of Mental Disorders, Fourth Edition-Text Revision (DSM-IV) criteria for major depression may be included; History of schizophrenia, bipolar disorder, or severe mental illness; History of alcohol or drug abuse; Have asthma, chronic obstructive pulmonary disease (COPD), or other breathing disease that is not controlled with medicine; Have human immunodeficiency virus (HIV) or syphilis; Are taking blood thinners. |
| ***Study Design*** | Phase I/Phase II randomized, parallel-group study |
| ***Efficacy Outcomes*** | The efficacy measure included ADAS-cog, CDR-SB, and MMSE. |
| ***Safety Outcomes*** | Adverse events and serious adverse events. |

| **Trials** | **NCT02956486** |
| --- | --- |
| ***Inclusion Criteria*** | Mild cognitive impairment due to Alzheimer's disease (AD) or mild AD dementia including: MMSE score equal to or greater than 24; Clinical Dementia Rating (CDR) global score of 0.5; CDR Memory Box score of 0.5 or greater; Impaired episodic memory confirmed by a list learning task; Positive biomarker for brain amyloid pathology as indicated by either amyloid positron emission tomography or cerebrospinal fluid AD assessment or both |
| ***Exclusion Criteria*** | Females who are breastfeeding or pregnant at Screening or Baseline. Females of child-bearing potential must use a highly effective method of contraception throughout the entire study period and for 28 days after study drug discontinuation; Any condition that may be contributing to cognitive impairment above and beyond that caused by the participant's AD; Participants with a history of seizures within 5 years of Screening; History of transient ischemic attacks or stroke within 12 months of Screening; Psychiatric diagnosis or symptoms (example, hallucinations, major depression, delusions etc.); Suicidal ideation or any suicidal behavior within 6 months before Screening or has been hospitalized or treated for suicidal behavior in the past 5 years; Have any contraindications to magnetic resonance imaging (MRI) scanning or Have lesions that could indicate a dementia diagnosis other than AD on brain MRI; Exhibit other significant pathological findings on brain MRI; Participants who have a history of moderate to severe hepatic impairment (example, Child-Pugh Class B or C); Results of laboratory tests conducted during Screening that are outside the following limits: Absolute lymphocyte count below the lower limit of normal (LLN), Thyroid stimulating hormone above normal range, Abnormally low Vitamin B12 levels. Participants at increased risk of infection; Have received any live vaccine/live attenuated vaccine in the 3 months before randomization; Any chronic inflammatory disease that is not adequately controlled or that requires systemic immunosuppressive or immunomodulatory therapy; Any other clinically significant abnormalities; Severe visual or hearing impairment; A prolonged corrected QT (QTc) interval (QT interval with Fridericia's correction [QTcF] greater than 450 milliseconds [ms]); Malignant neoplasms within 5 years of Screening; Known or suspected history of drug or alcohol abuse; Taking prohibited medications, which must be reviewed with the Investigator; Have participated in a recent clinical study |
| ***Study Design*** | Phase III placebo-controlled, double-blind, parallel-group study |
| ***Efficacy Outcomes*** | The efficacy measure included ADAS-cog, CDR-SB, and MMSE. |
| ***Safety Outcomes*** | Adverse events and serious adverse events. |

**Table S4: Quality assessment of the included studies.**

| Items | Clear description of purpose | Clear diagnostic criteria of AD | Description of statistical analysis | Clear description of eligibility criteria | Long enough follow-up period | Limitations considered | Patients’ consent of resaerch |
| --- | --- | --- | --- | --- | --- | --- | --- |
| 2003 Ritchie CW | 1 | 1 | 0 | 0 | 0 | 0 | 1 |
| 2005 Gilman S | 1 | 1 | 1 | 1 | 0 | 0 | 1 |
| 2008 Fleisher AS | 1 | 1 | 1 | 1 | 0 | 1 | 1 |
| 2008 Lannfelt L | 1 | 1 | 1 | 1 | 0 | 1 | 1 |
| 2008 Wilcock GK | 1 | 1 | 1 | 1 | 0 | 0 | 1 |
| 2009 Green RC | 1 | 1 | 1 | 1 | 1 | 0 | 1 |
| 2009 Salloway S | 1 | 0 | 1 | 1 | 1 | 1 | 1 |
| 2011 Aisen PS | 1 | 1 | 1 | 1 | 1 | 1 | 1 |
| 2012 Farlow M | 1 | 0 | 1 | 1 | 0 | 1 | 1 |
| 2012 Vladimir C | 1 | 1 | 1 | 1 | 0 | 1 | 1 |
| 2013 Dodel R | 1 | 1 | 1 | 1 | 0 | 1 | 1 |
| 2013 Doody RS | 1 | 1 | 1 | 1 | 1 | 1 | 1 |
| 2014 Salloway S | 1 | 1 | 1 | 1 | 1 | 0 | 1 |
| 2015 Arai H | 1 | 1 | 1 | 1 | 1 | 1 | 1 |
| 2015 Farlow MF | 1 | 0 | 1 | 0 | 0 | 1 | 1 |
| 2015 Vladimir C | 1 | 1 | 1 | 1 | 1 | 1 | 1 |
| 2016 CH van Dyck | 1 | 1 | 1 | 1 | 1 | 1 | 1 |
| 2016 Delnomdedieu M | 1 | 1 | 0 | 1 | 0 | 1 | 1 |
| 2016 Pasquier F | 1 | 1 | 1 | 1 | 1 | 1 | 1 |
| 2016 Vandenberghe R | 1 | 0 | 1 | 1 | 1 | 1 | 1 |
| 2016 Vandenberghe R NCT01097096 | 1 | 1 | 1 | 1 | 1 | 1 | 1 |
| 2017 Landen JW | 1 | 1 | 1 | 1 | 1 | 0 | 1 |
| 2017 Ostrowitzki S | 1 | 1 | 1 | 1 | 1 | 1 | 1 |
| 2017 Relkin NR | 1 | 1 | 1 | 1 | 1 | 1 | 1 |
| 2018 Cummings JL | 1 | 1 | 1 | 1 | 1 | 0 | 1 |
| 2018 Egan MF | 1 | 1 | 1 | 1 | 1 | 0 | 1 |
| 2018 Honig LS | 1 | 1 | 1 | 1 | 1 | 0 | 1 |
| 2018 Salloway S | 1 | 1 | 1 | 1 | 1 | 1 | 1 |
| 2019 Egan MF | 1 | 0 | 1 | 1 | 1 | 0 | 1 |
| 2019 Wessels AM | 1 | 0 | 1 | 1 | 1 | 1 | 1 |
| 2021 Lo AC | 1 | 0 | 1 | 0 | 0 | 0 | 1 |
| 2021 Mintun MA | 1 | 1 | 1 | 1 | 1 | 1 | 1 |
| 2021 Swanson CJ | 1 | 1 | 1 | 1 | 1 | 1 | 1 |
| 2022 Haeberlein SB | 1 | 1 | 1 | 1 | 1 | 1 | 1 |
| 2022 Ostrowitzki S | 1 | 1 | 1 | 1 | 1 | 1 | 1 |
| 2023 Christopher H | 1 | 1 | 1 | 1 | 1 | 1 | 1 |

**Figure S1: Forest plot of meta-analysis: CDR-SB**

**
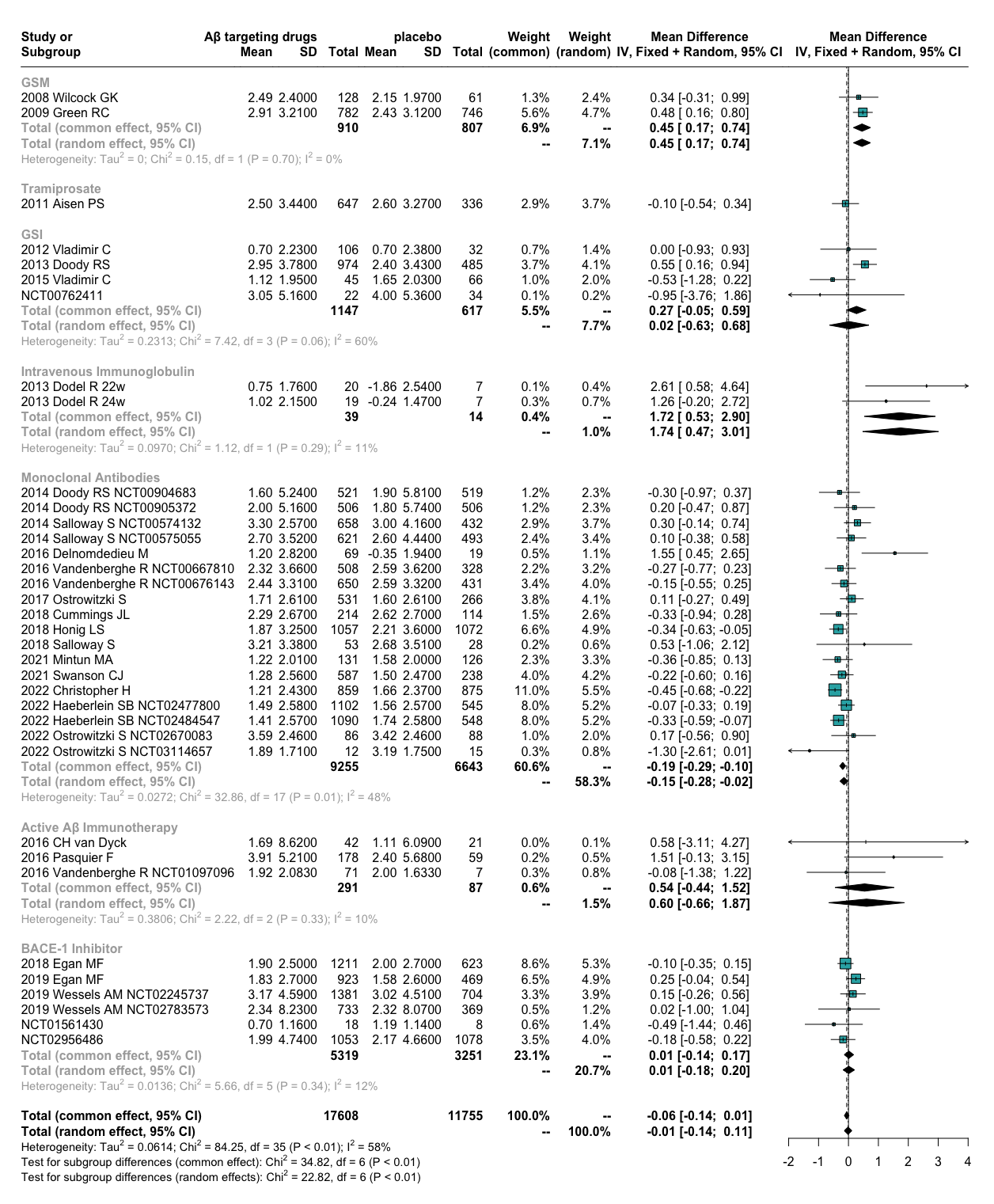
**

**Figure S2: Forest plot of meta-analysis: MMSE**

**
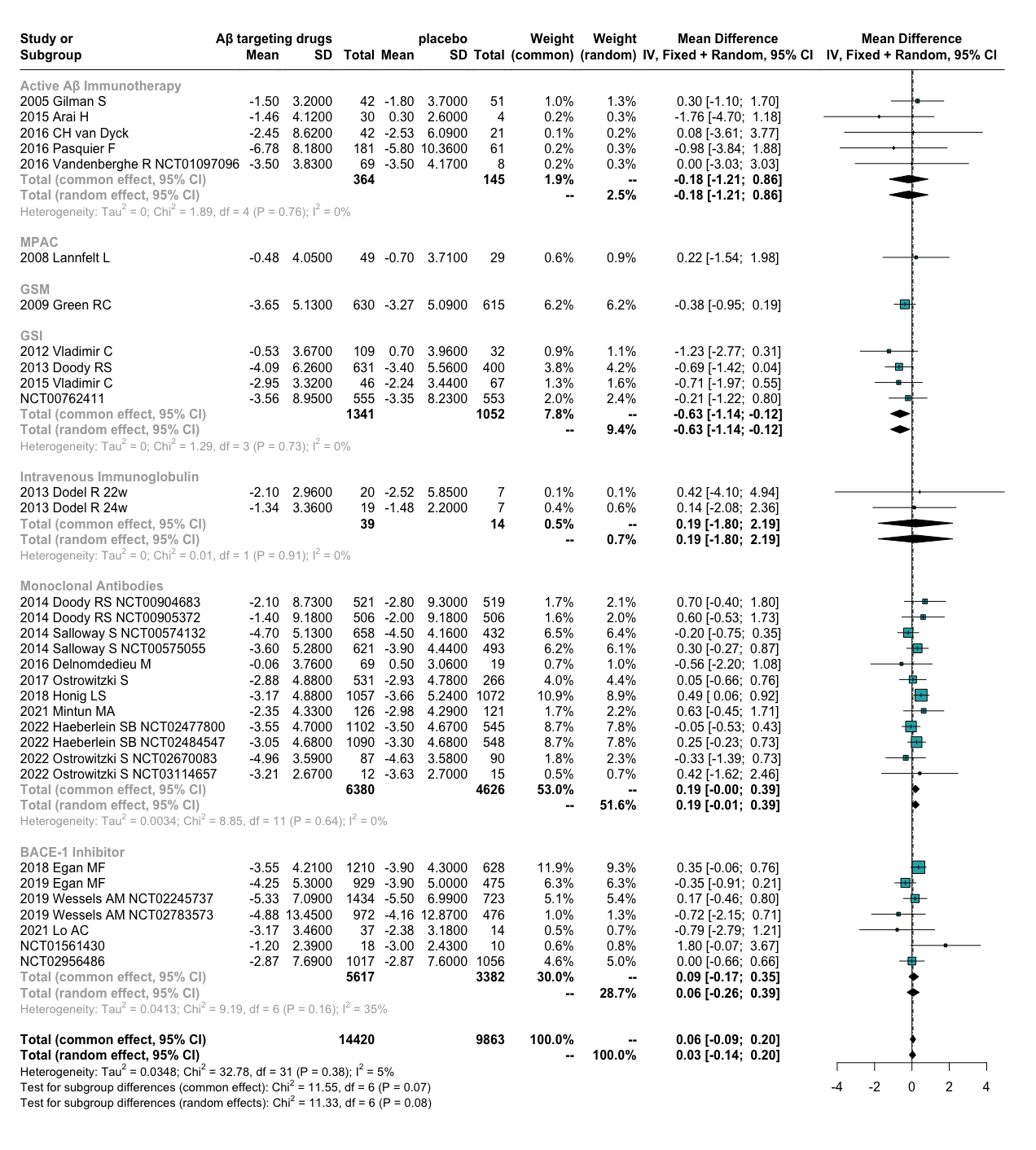
**

**Figure S3: Forest plot of meta-analysis: ADCS-ADL**

**
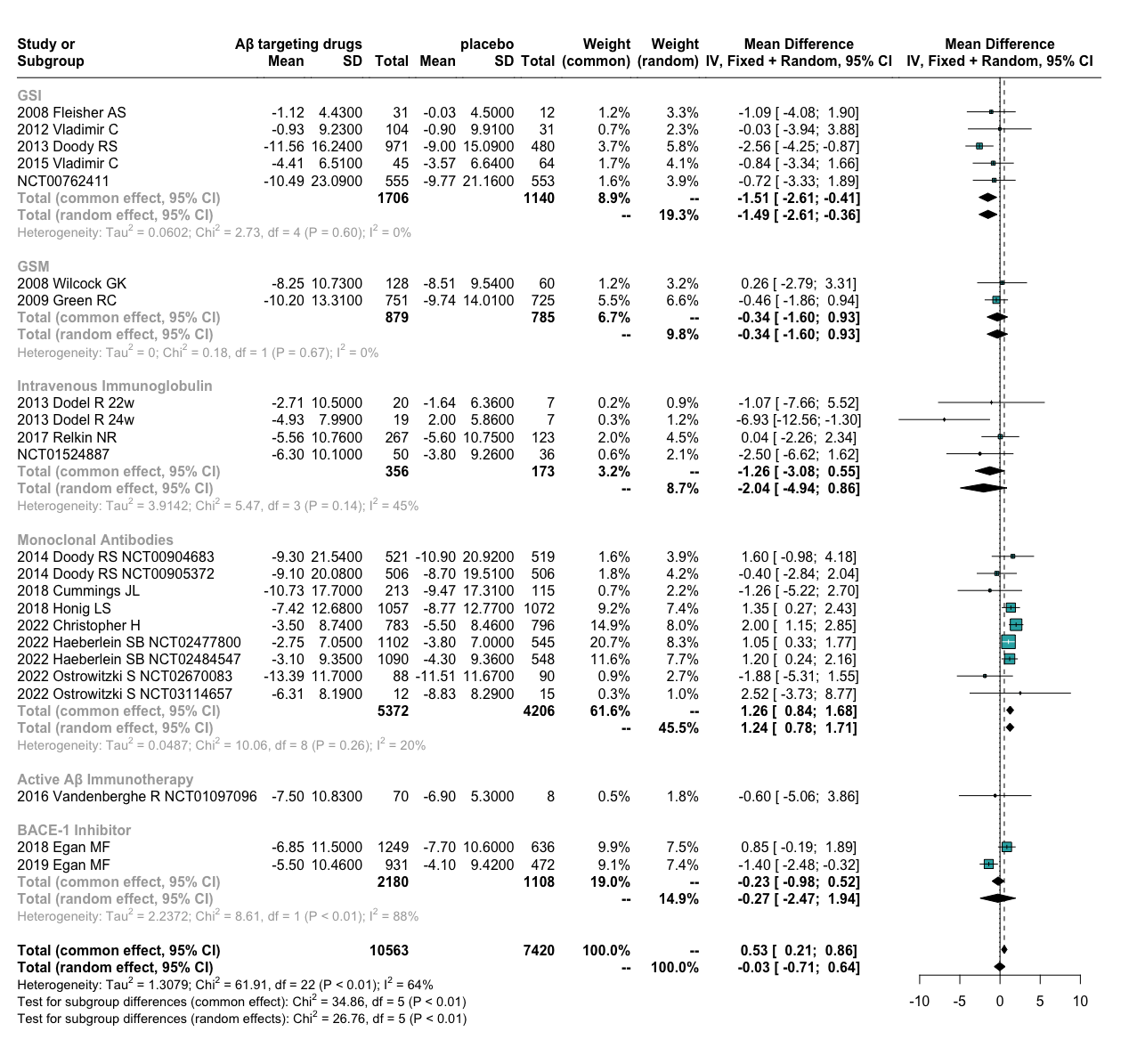
**

**Figure S4: Forest plot of meta-analysis: NPI**

**
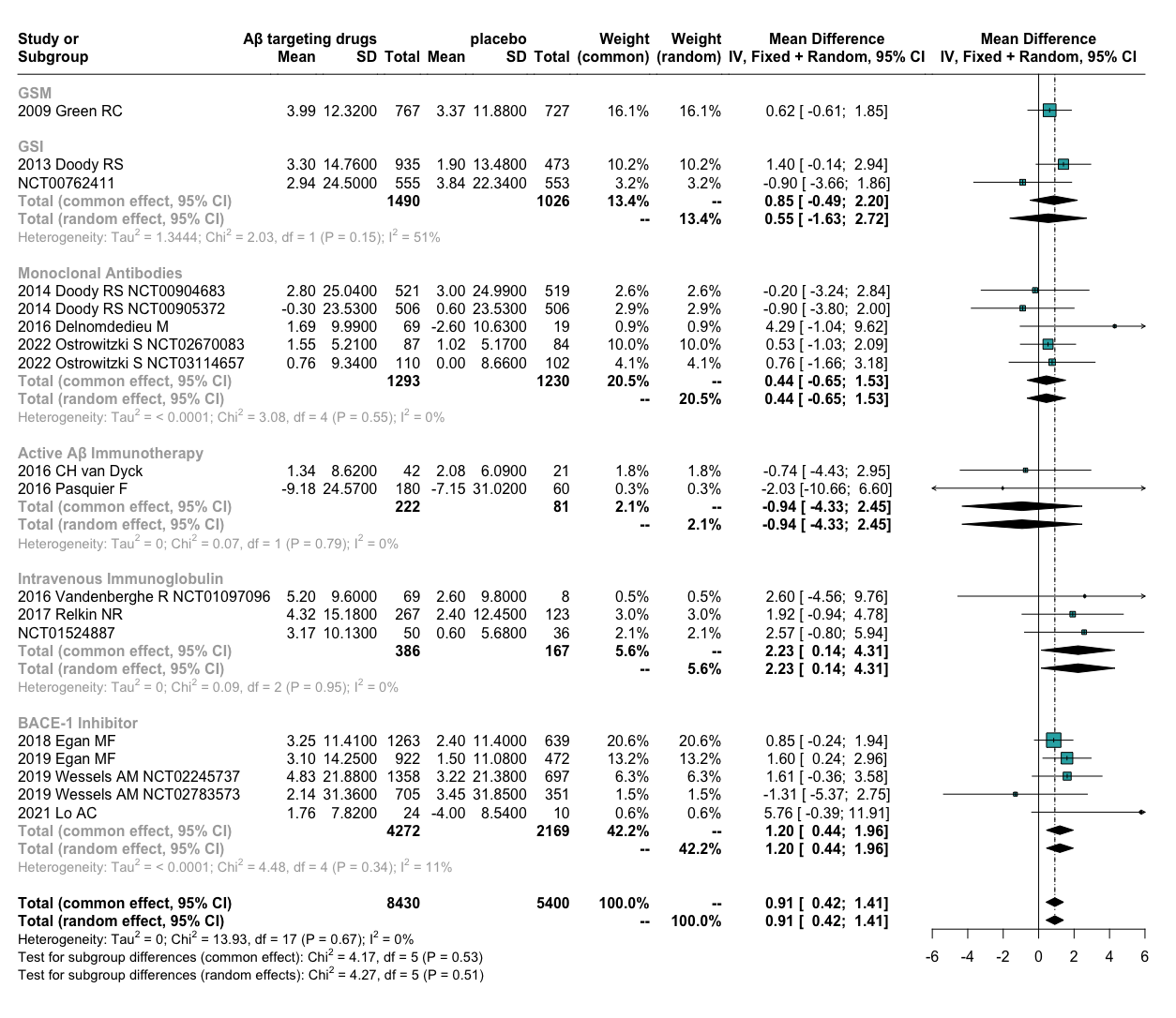
**

**Figure S5: Forest plot of meta-analysis: SAEs**

**
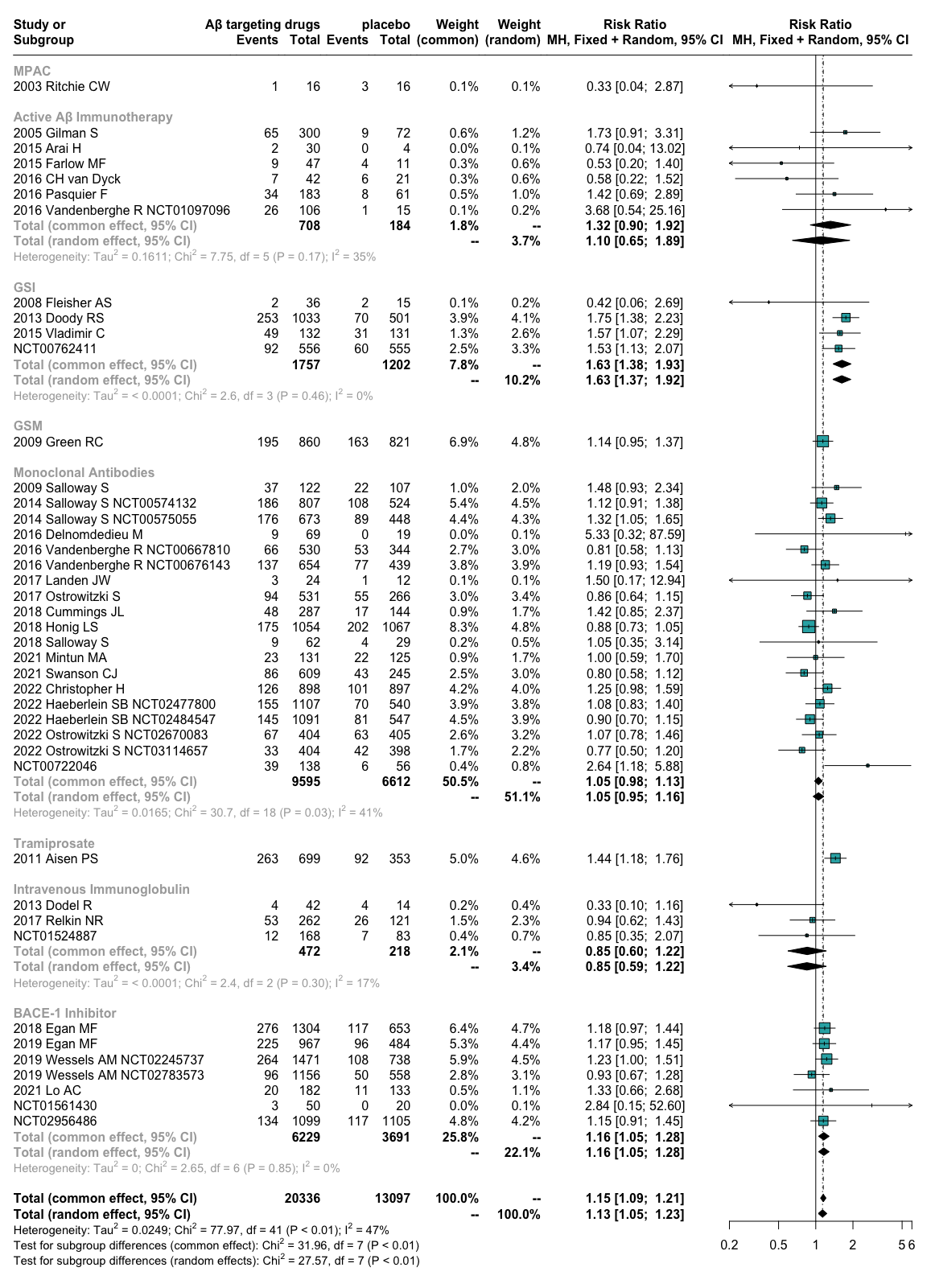
**

**Figure S6: Forest plot of meta-analysis: Death.**

**
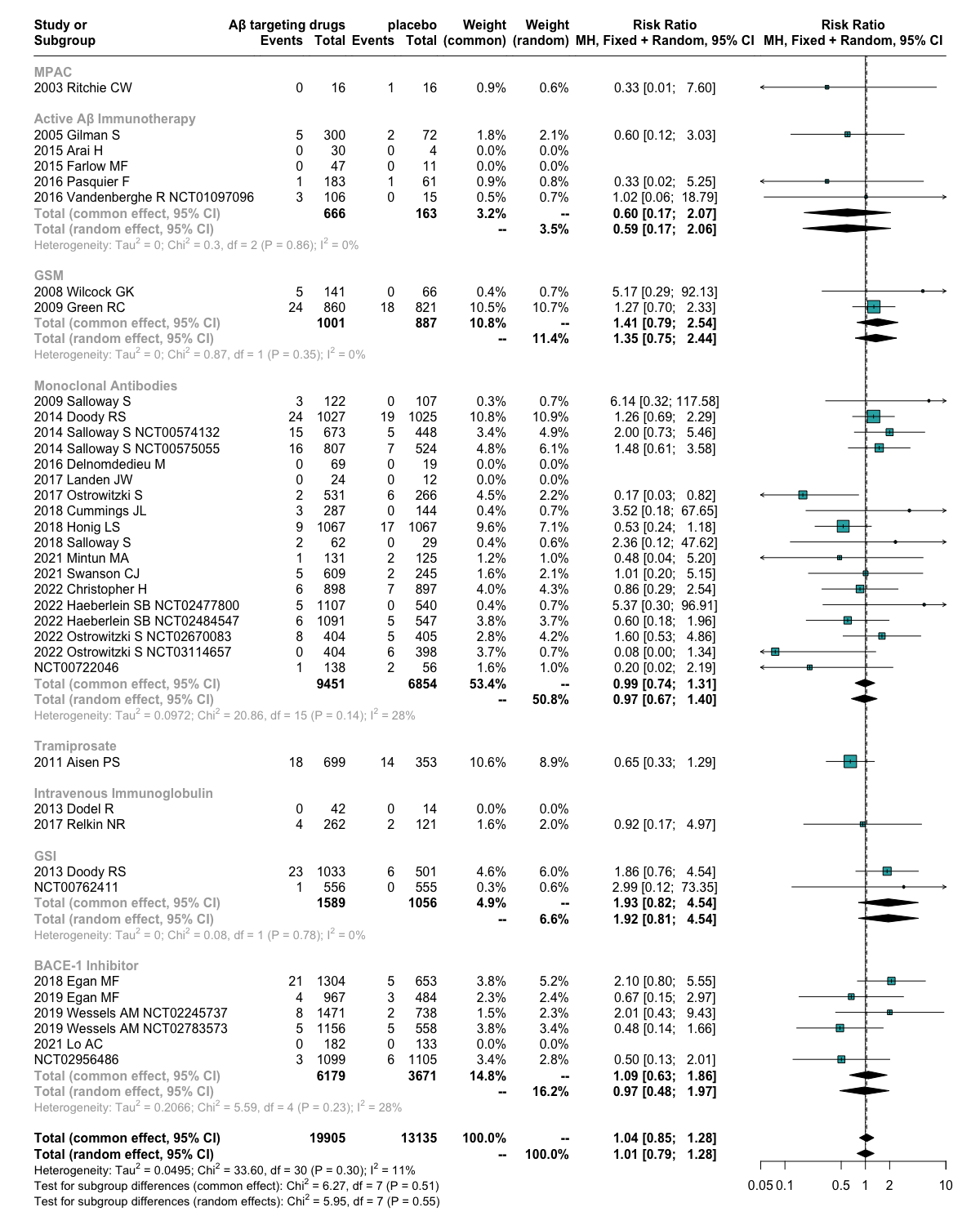
**

**Figure S7: Forest plot of sub-analysis based on different follow-up times: ADAS-Cog**


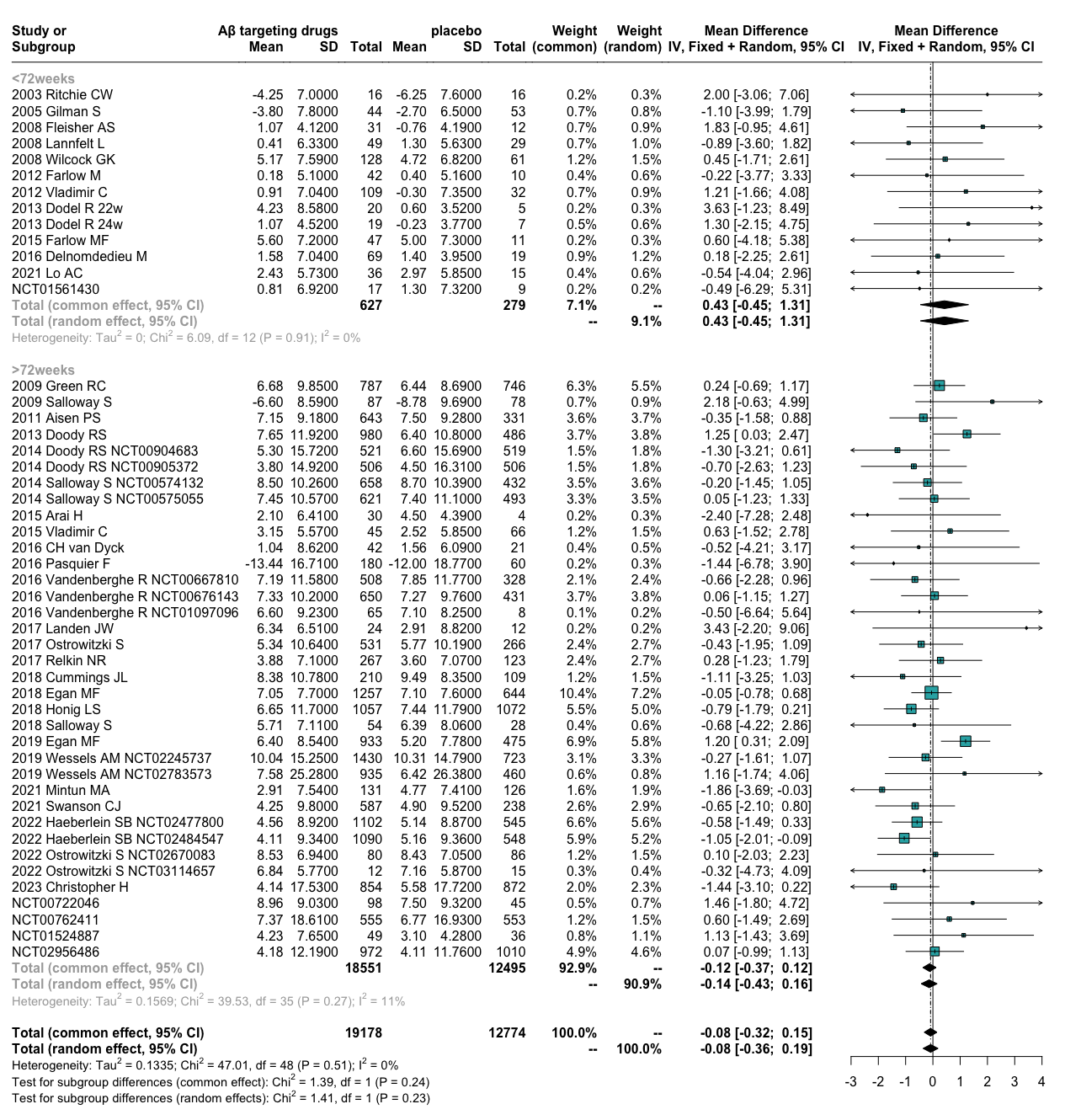


**Figure S8: Forest plot of sub-analysis based on different follow-up times: CDR-SB**


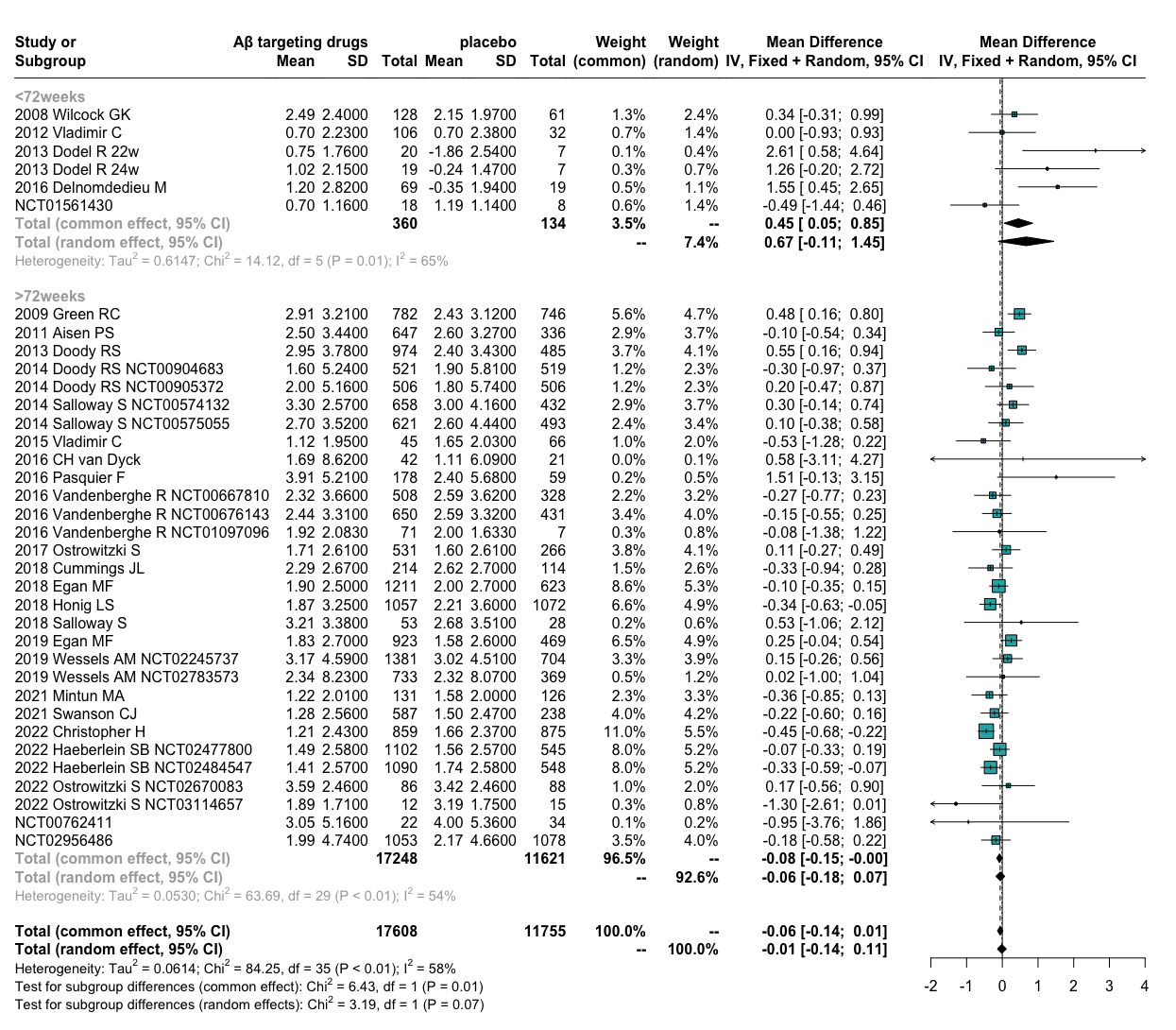


**Figure S9: Forest plot of sub-analysis based on different follow-up times: MMSE**


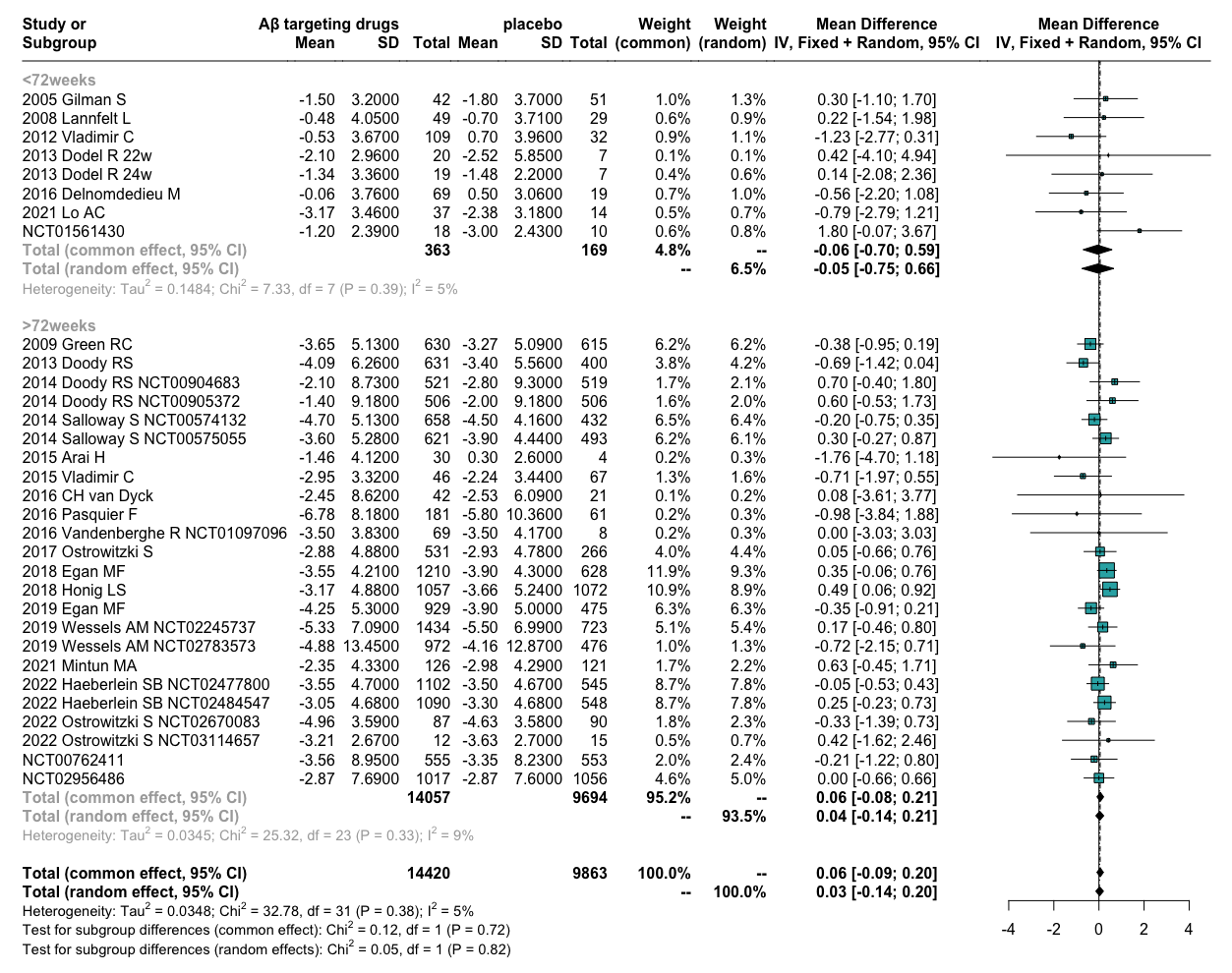


**Figure S10: Forest plot of sub-analysis based on different follow-up times: ADCS-ADL**


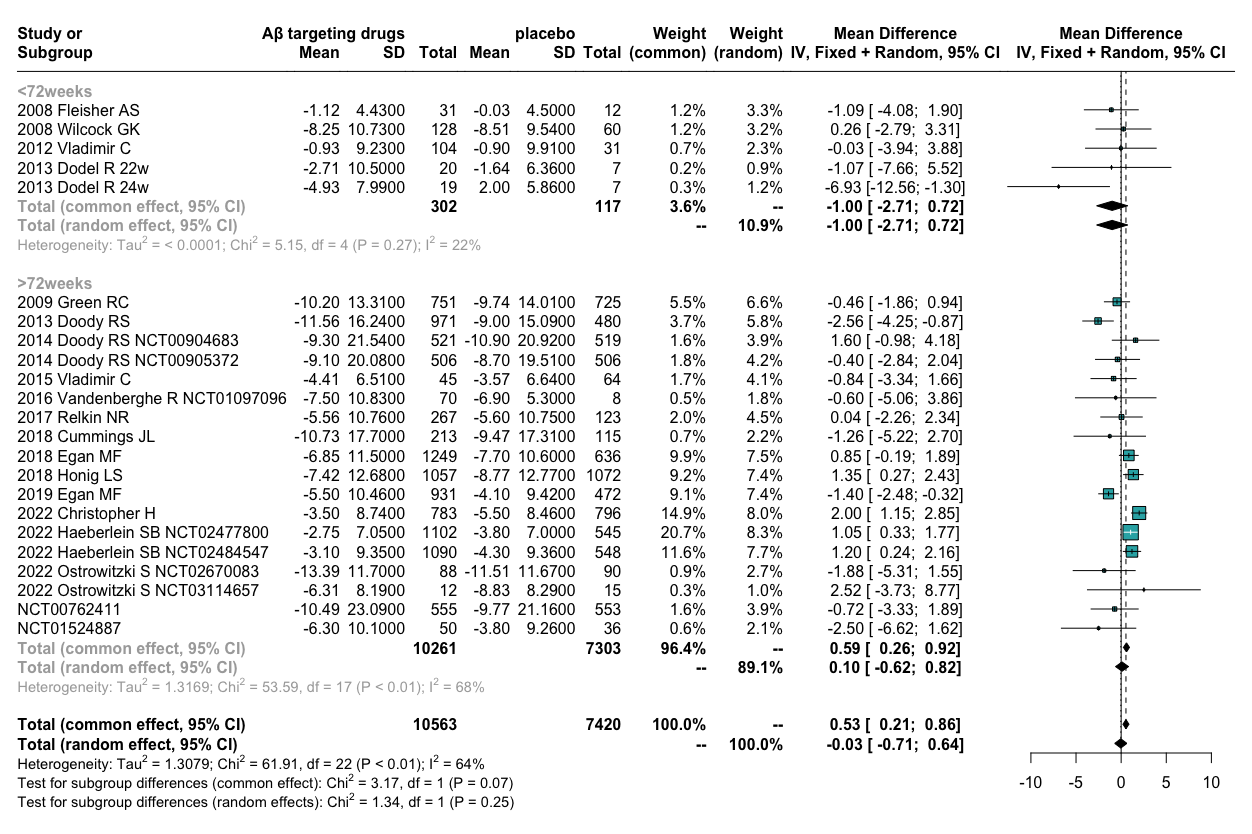


**Figure S11: Forest plot of sub-analysis based on different follow-up times: NPI**


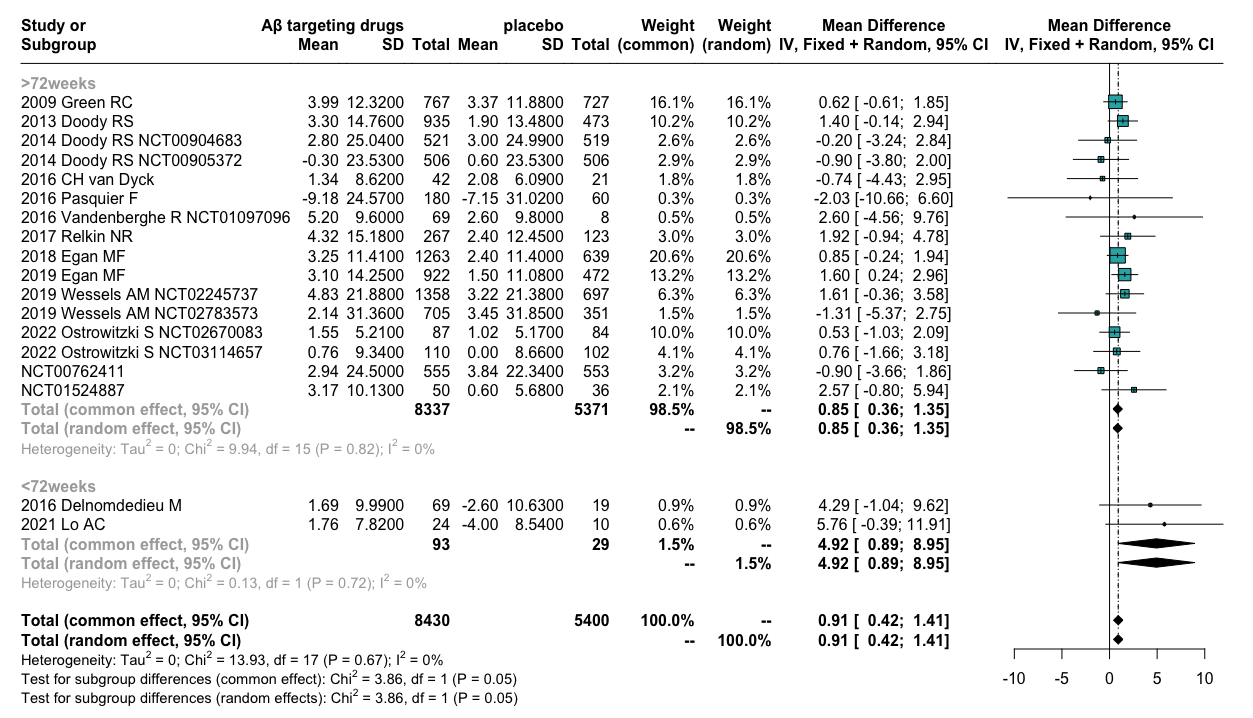


**Figure S12: Forest plot of sub-analysis based on different follow-up times: AEs**


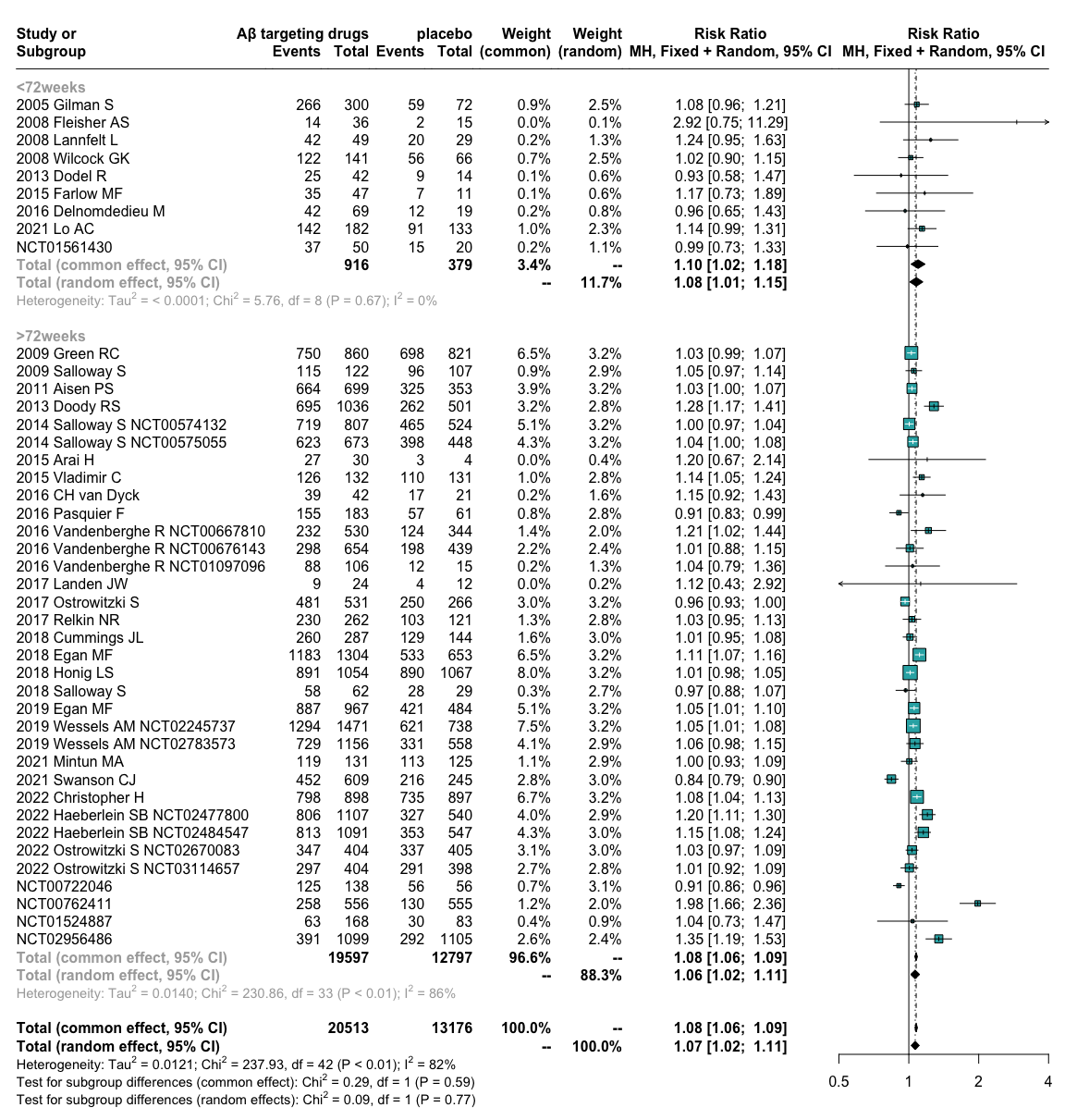


**Figure S13: Forest plot of sub-analysis based on different follow-up time: SAEs**


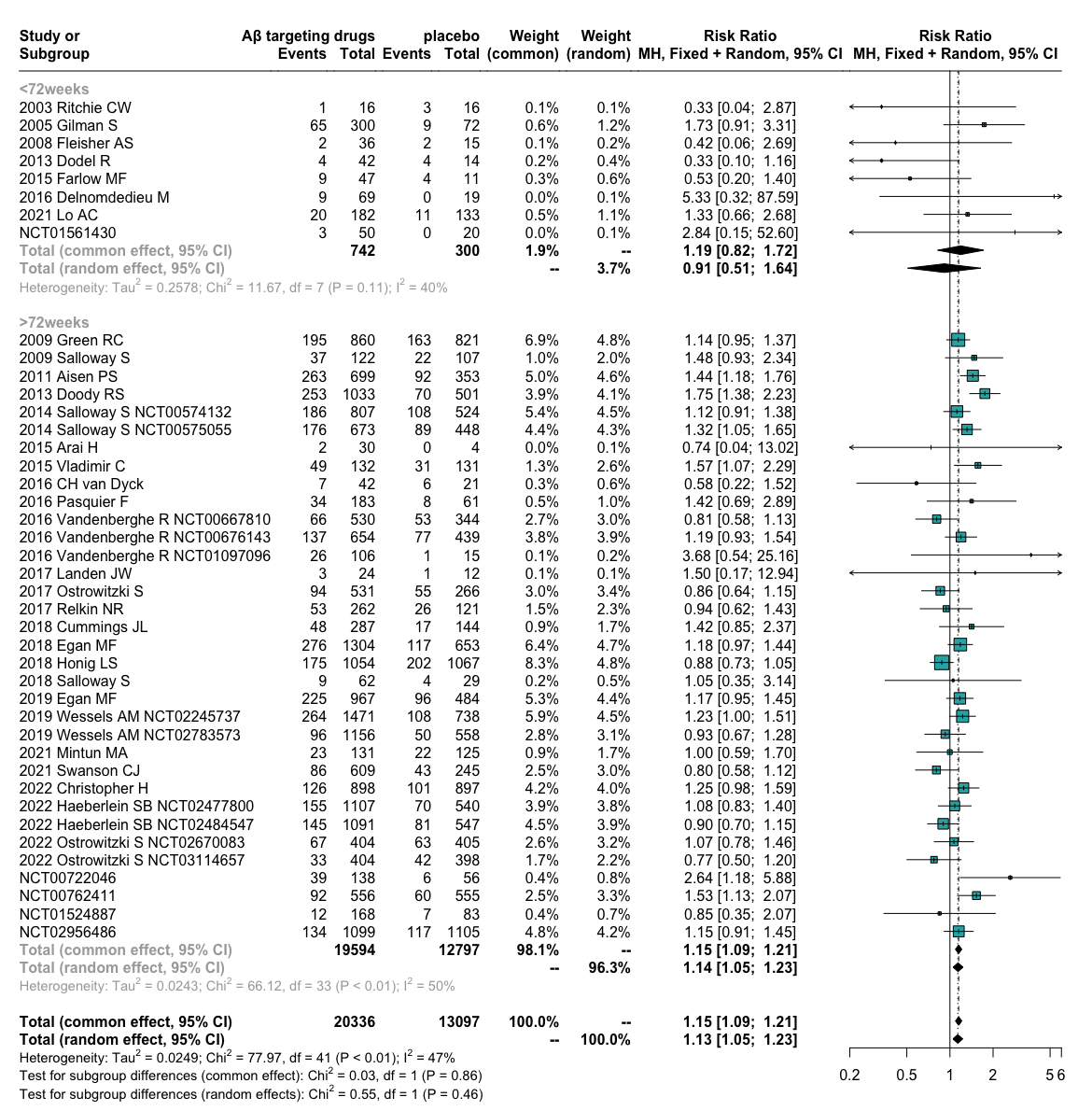


**Figure S14: Forest plot of sub-analysis based on different follow-up time: Death**


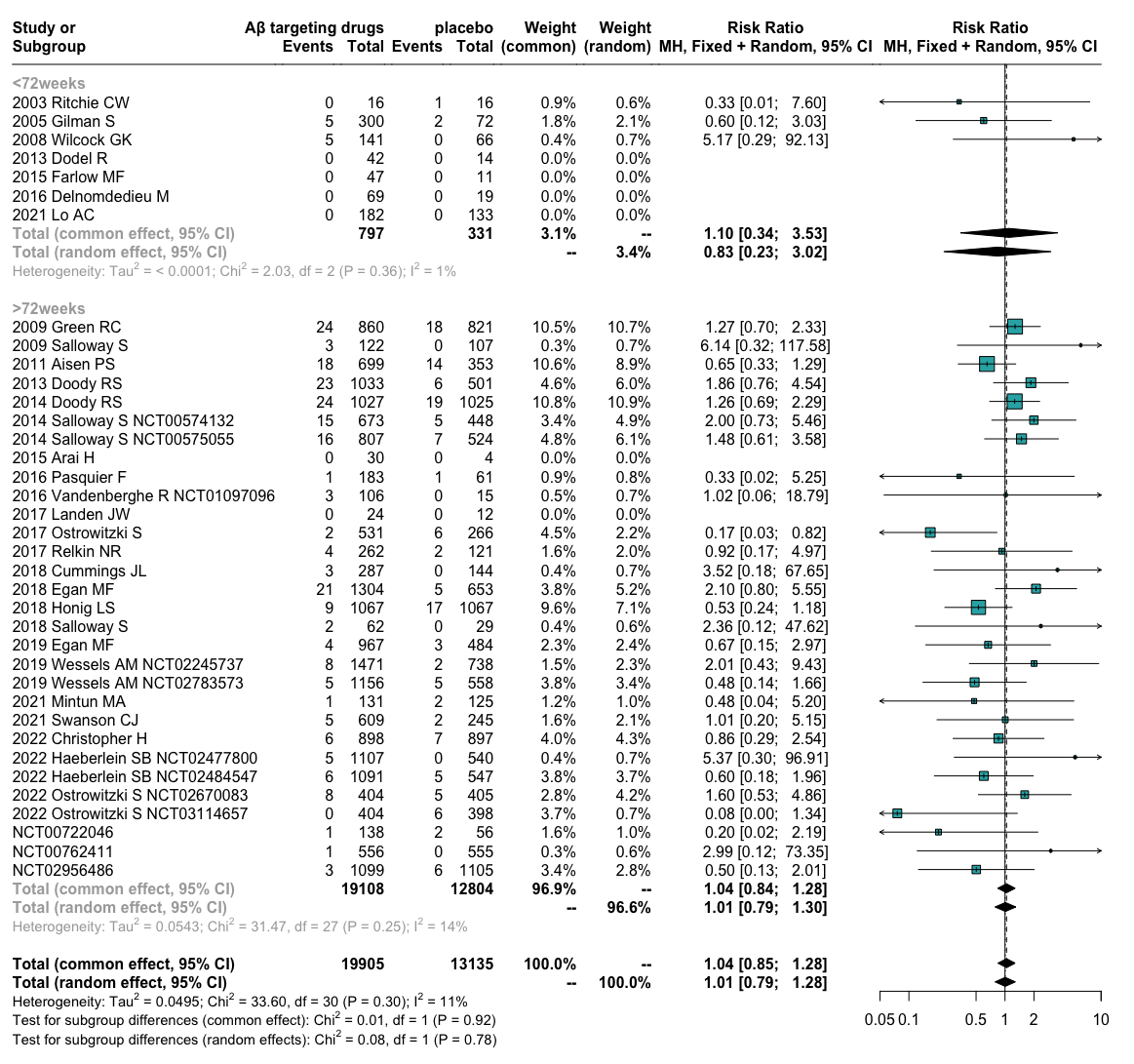


**Figure S15: Forest plot of ADAS-Cog in patients with mild cognitive impairment**


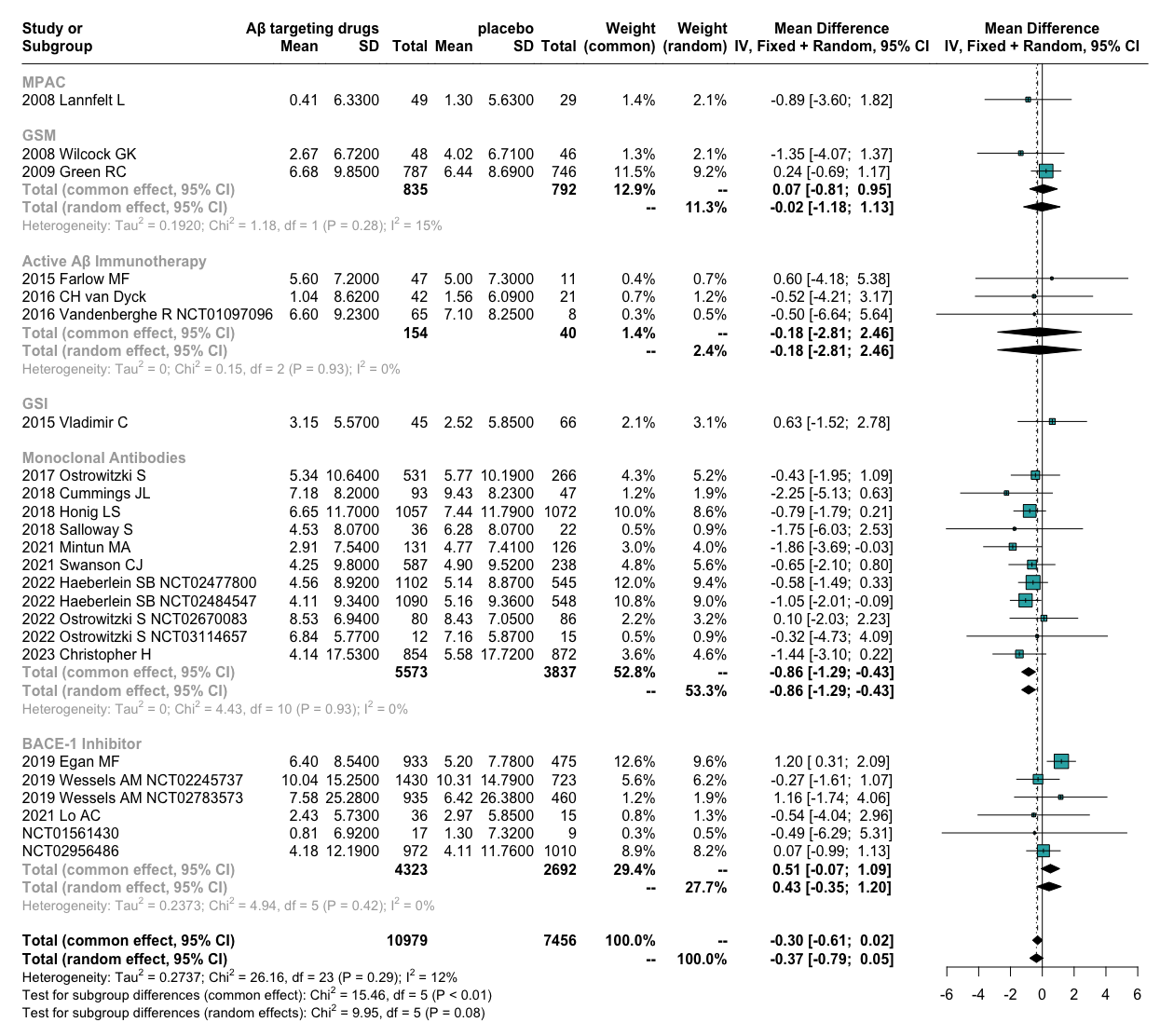


**Figure S16: Forest plot of CDR-SB in patients with mild cognitive impairment**


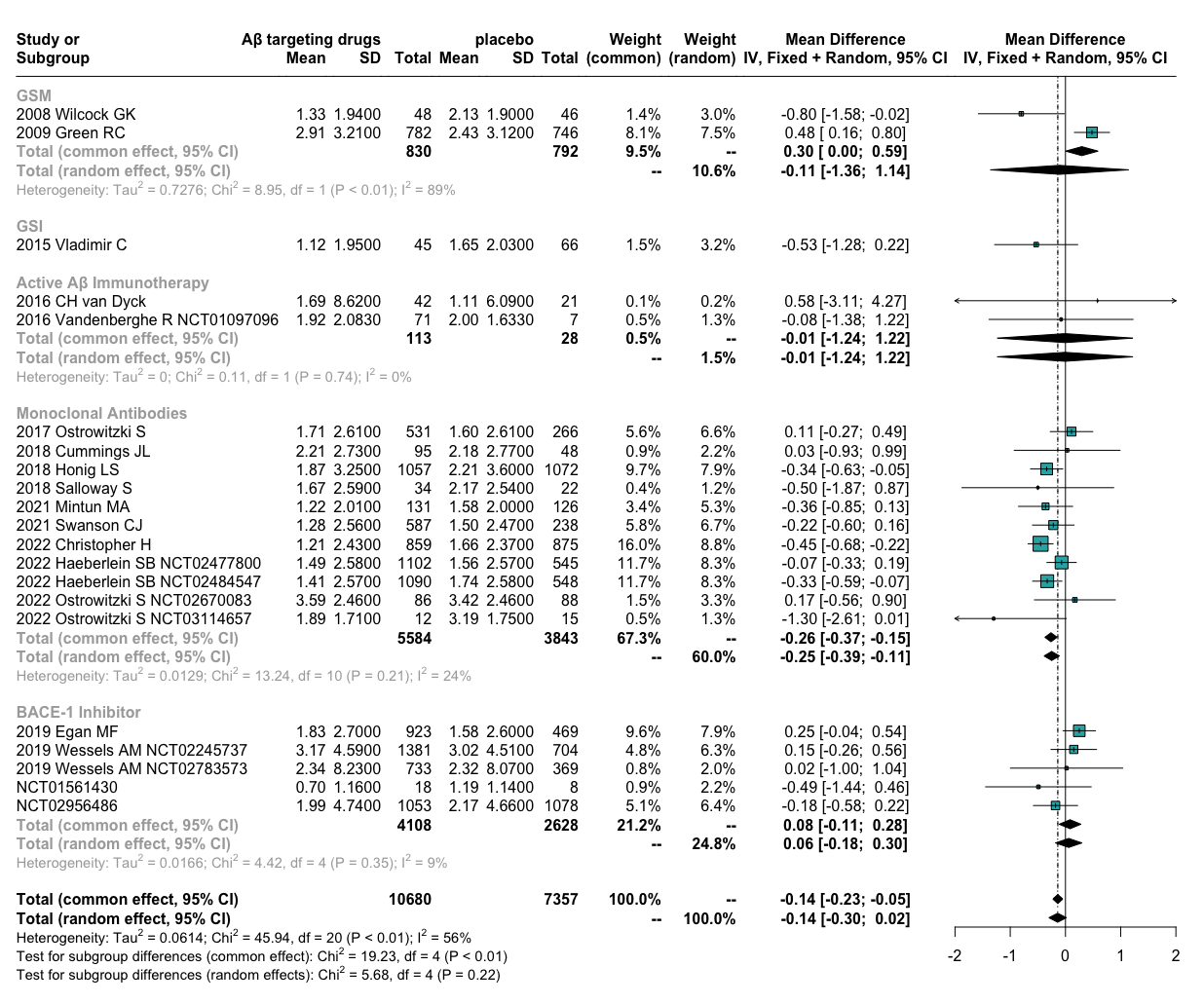


**Figure S17: Forest plot of MMSE in patients with mild cognitive impairment**


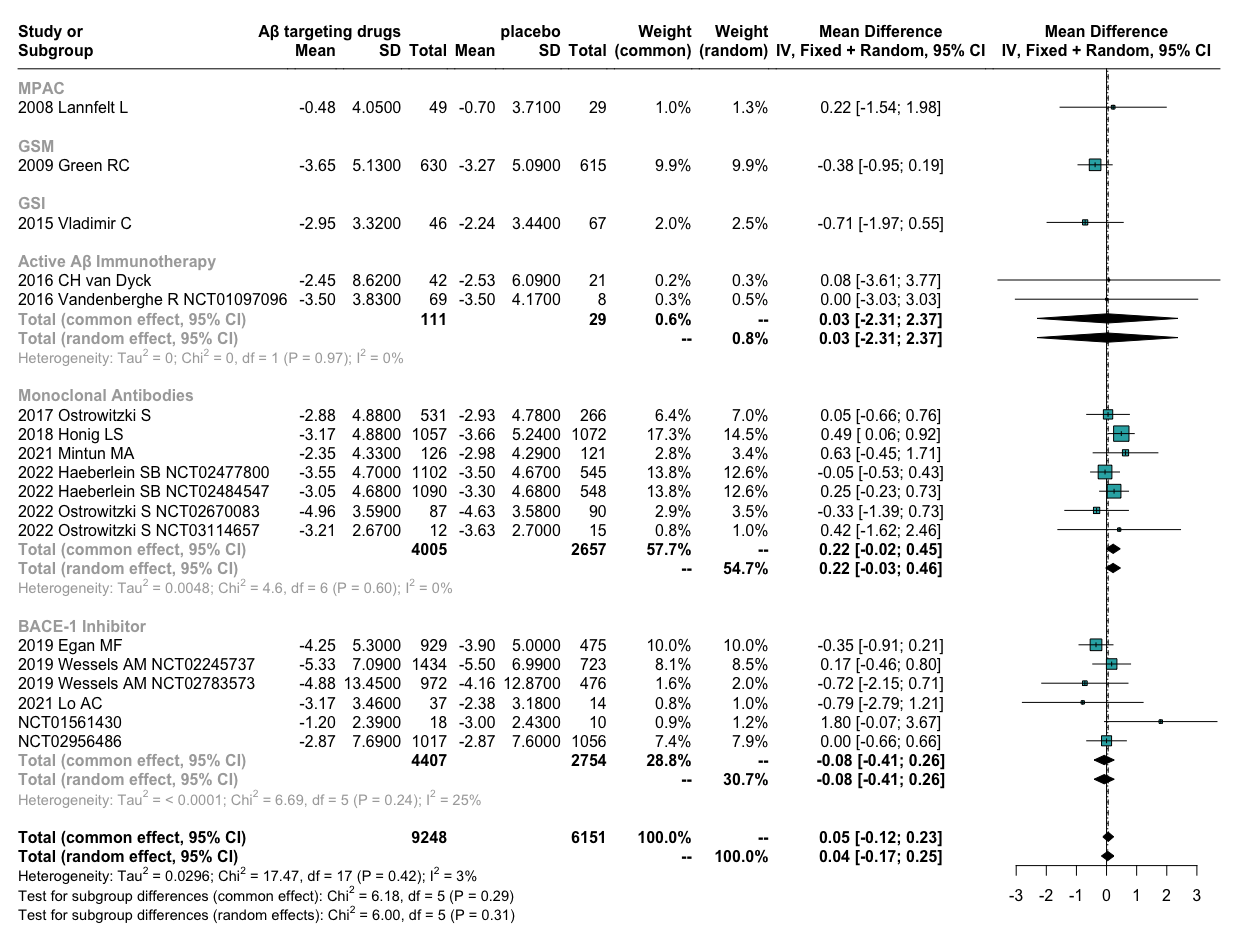


**Figure S18: Forest plot of ADCS-ADL in patients with mild cognitive impairment**


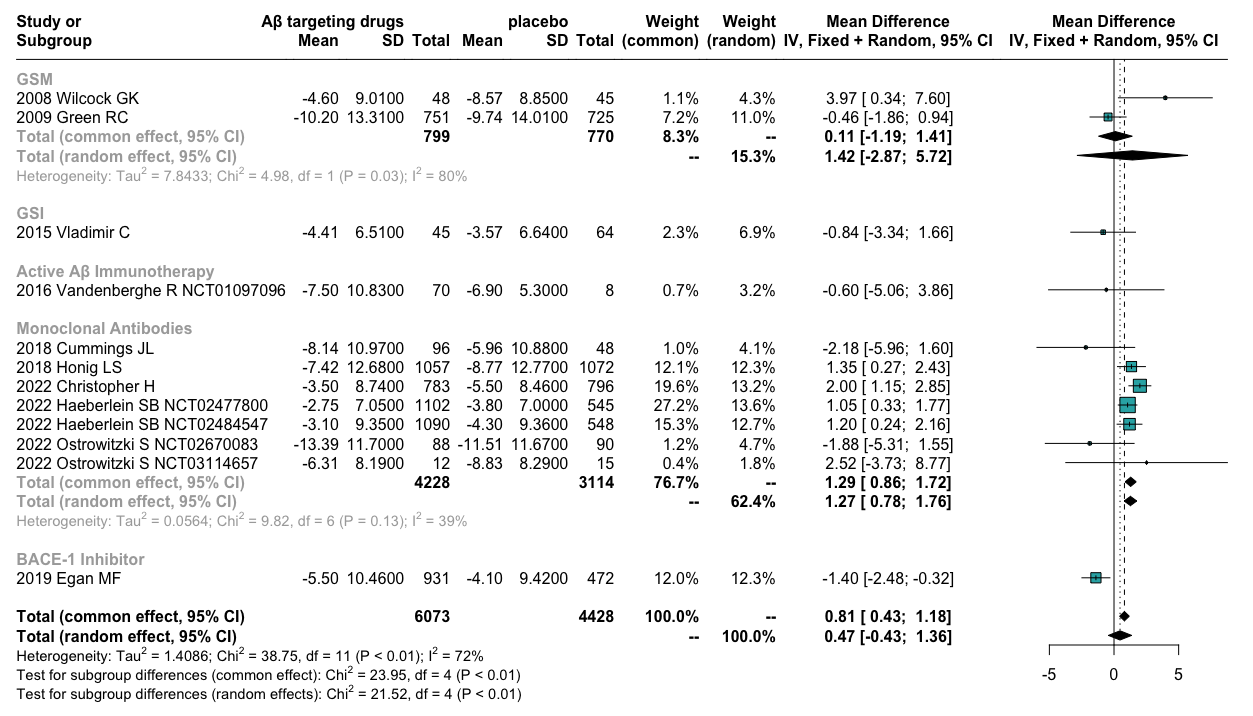


**Figure S19: Forest plot of NPI in patients with mild cognitive impairment**


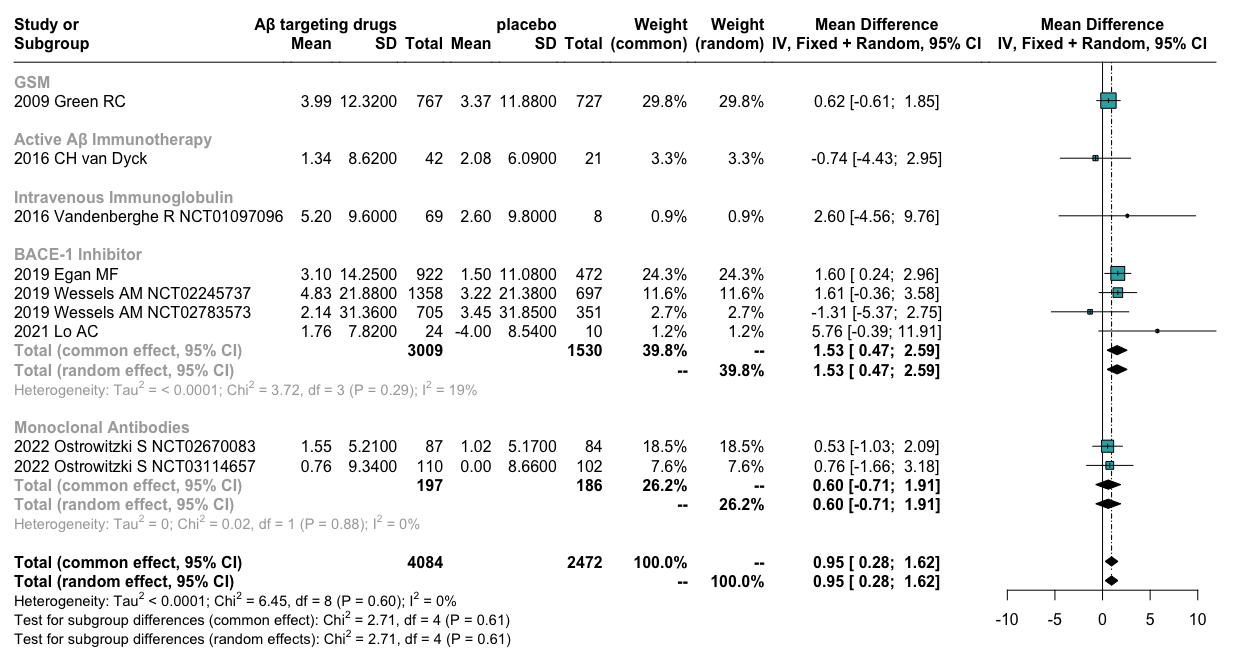


**Figure S20: Forest plot of AEs in patients with mild cognitive impairment**


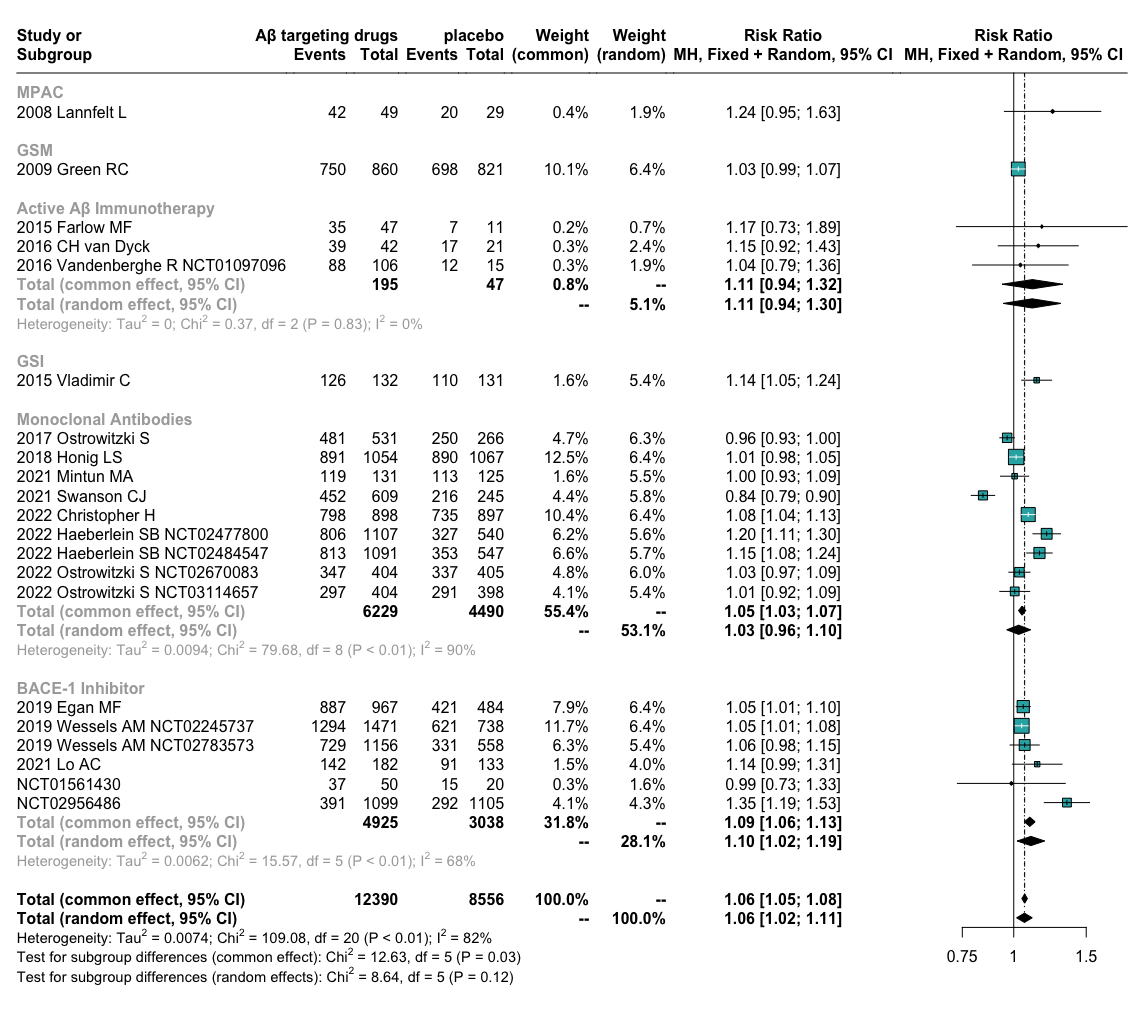


**Figure S21: Forest plot of SAEs in patients with mild cognitive impairment**


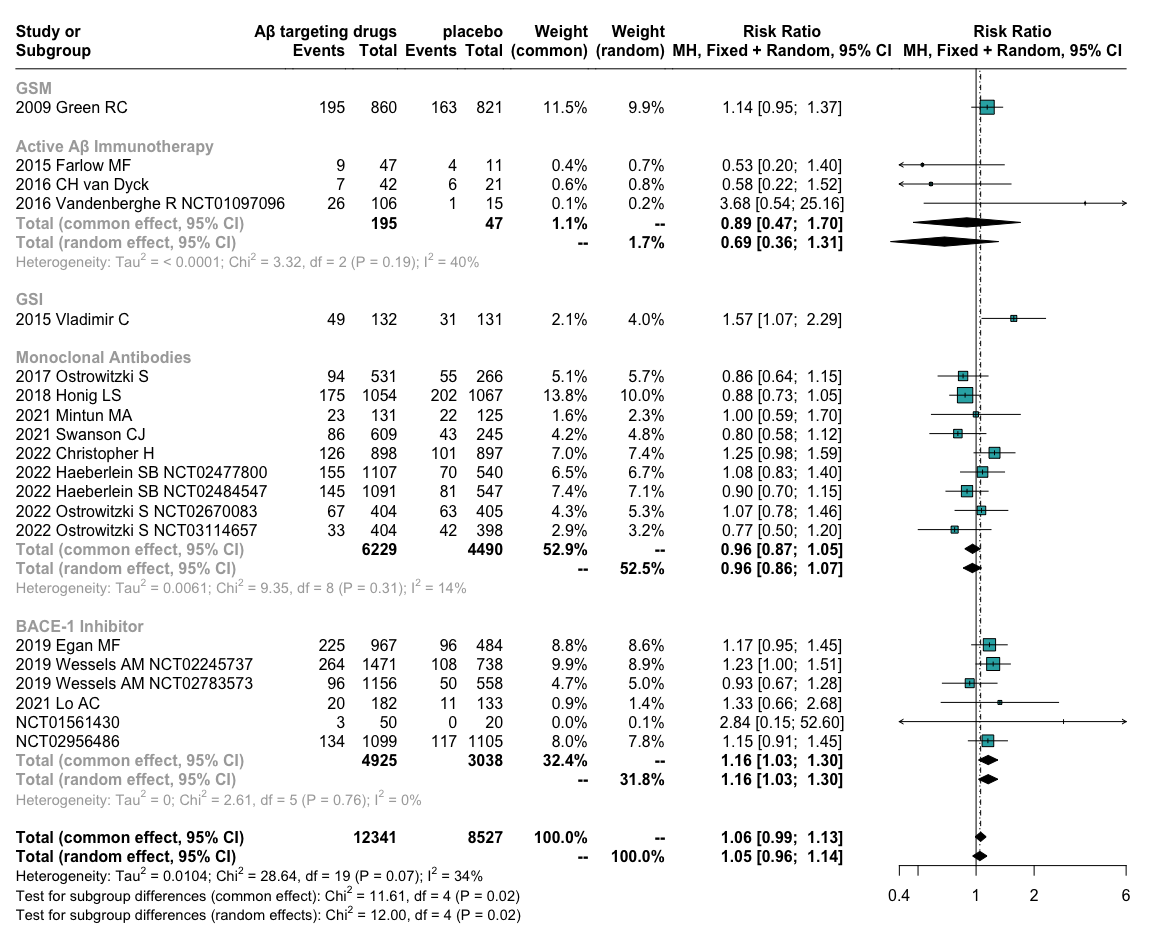


**Figure S22: Forest plot of Death in patients with mild cognitive impairment**


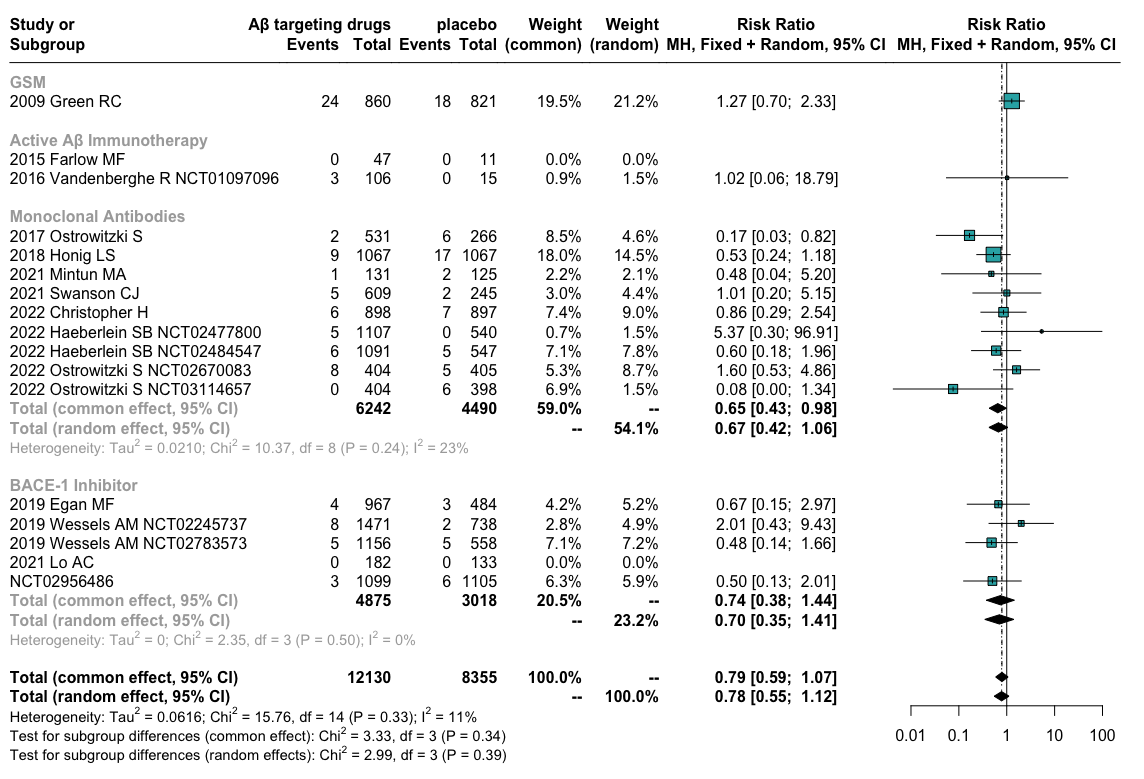


**Figure S23: Forest plot of sensitive analysis: ADAS-Cog**


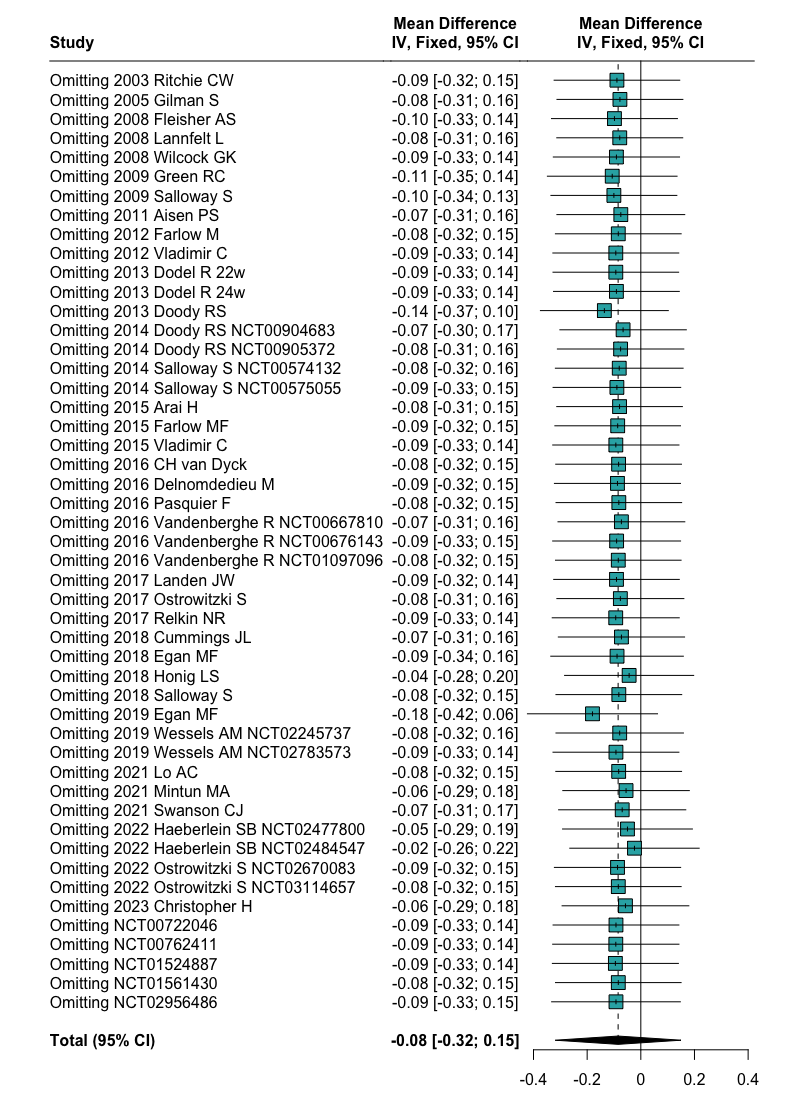


**Figure S24: Forest plot of sensitive analysis: CDR-SB**


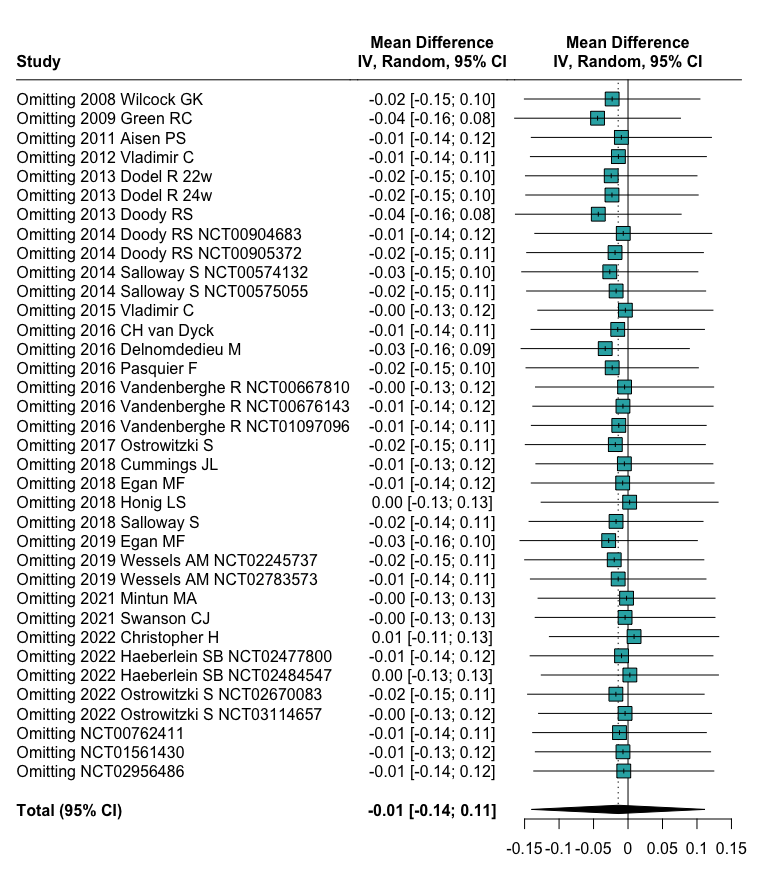


**Figure S25: Forest plot of sensitive analysis: MMSE**


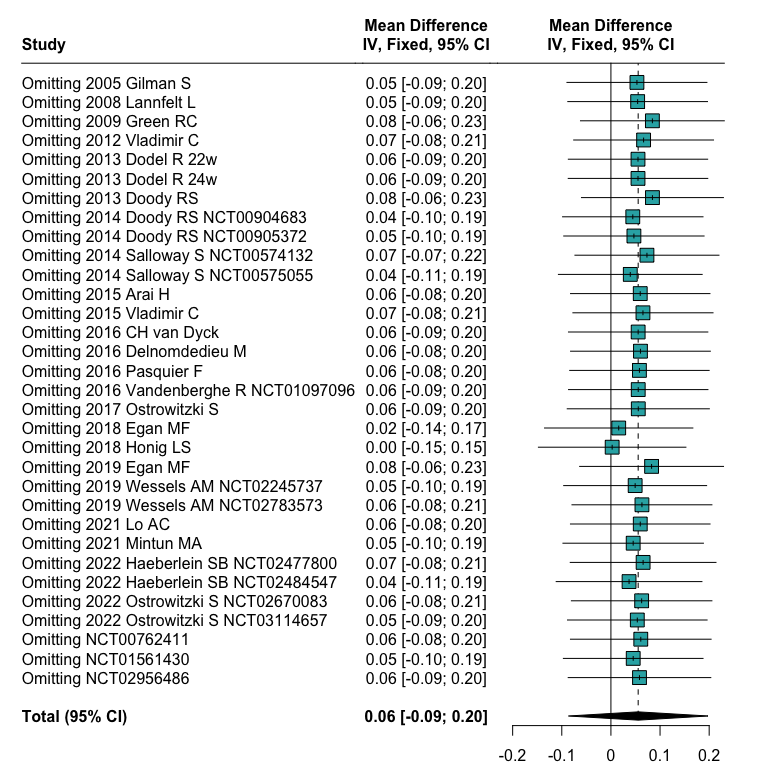


**Figure S26: Forest plot of sensitive analysis: MMSE**


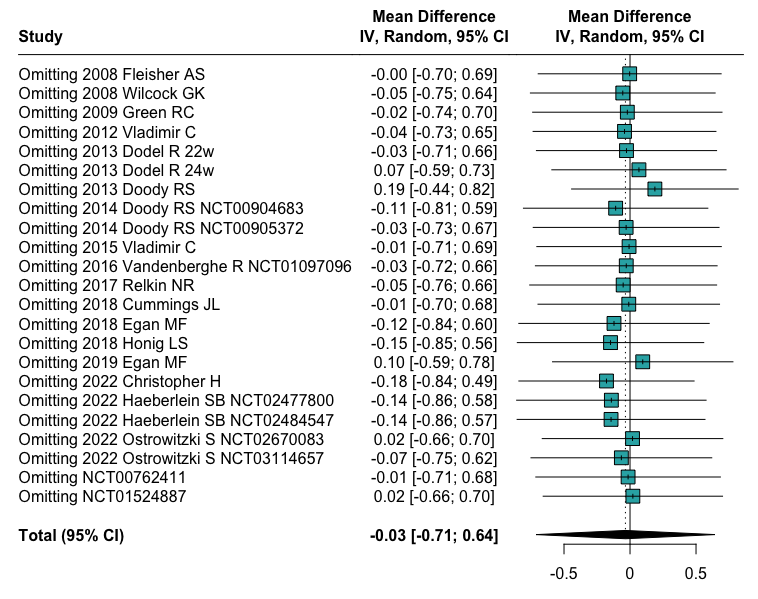


**Figure S27: Forest plot of sensitive analysis: NPI**


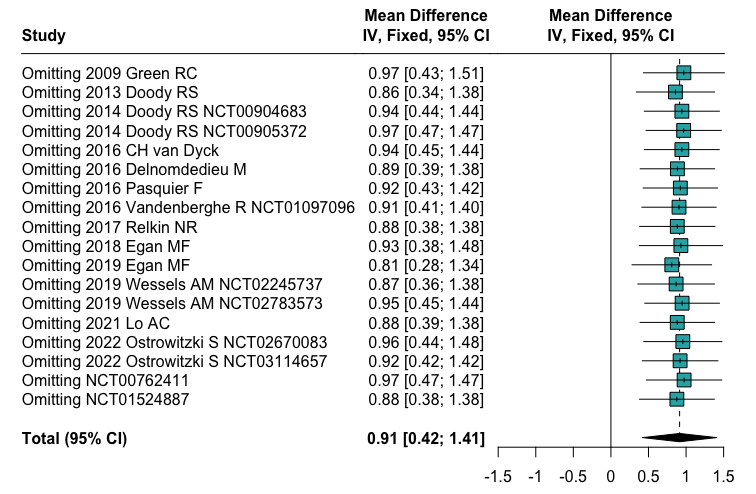


**Figure S28: Forest plot of sensitive analysis: AEs**


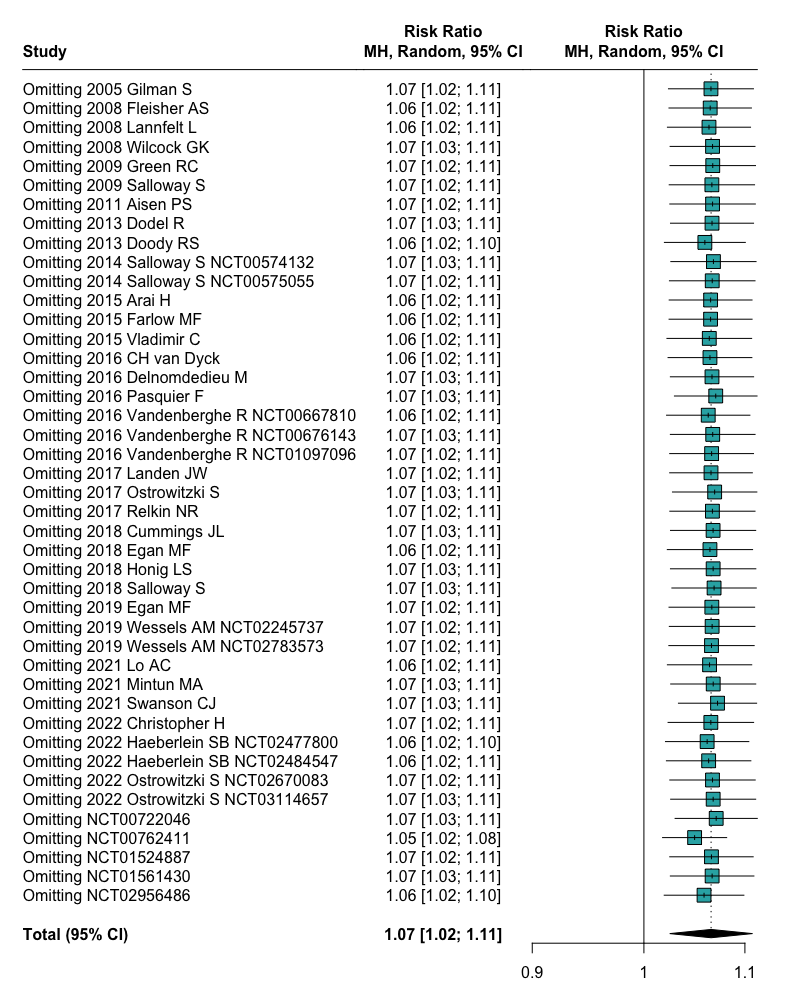


**Figure S29: Forest plot of sensitive analysis: SAEs**


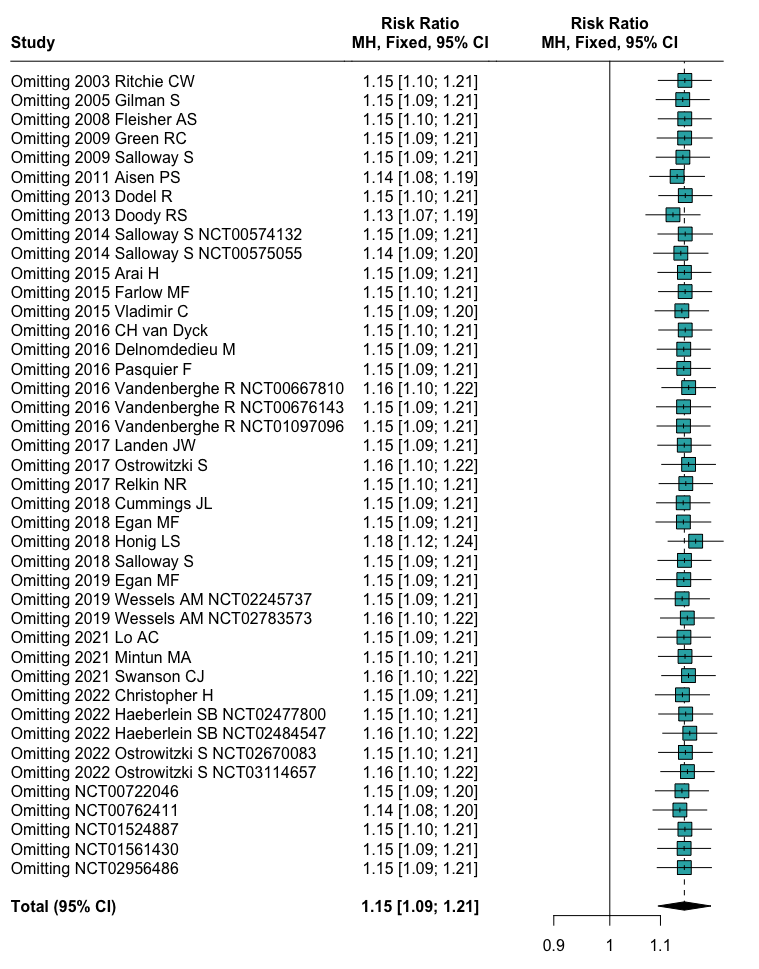


**Figure S30: Forest plot of sensitive analysis: Death**


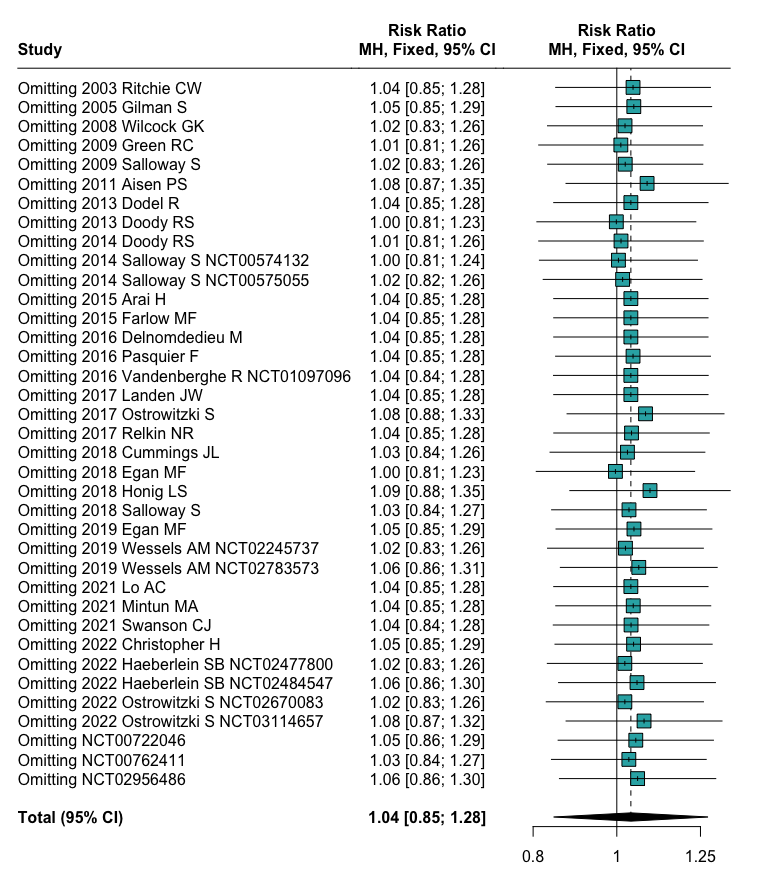


**Figure S31: Funnel plot of sensitive analysis: ADAS-Cog**


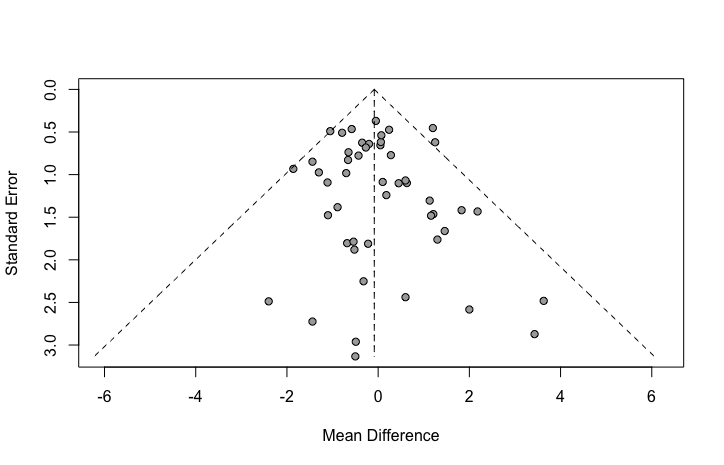


**Figure S32: Funnel plot of sensitive analysis: CDR-SB**


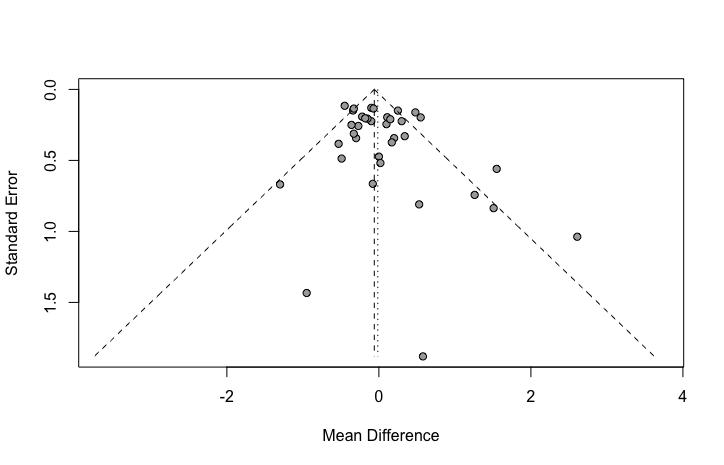


**Figure S33: Funnel plot of sensitive analysis: MMSE**


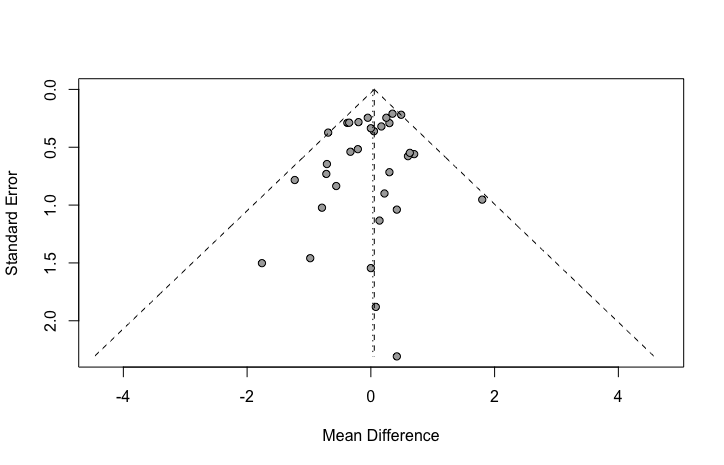


**Figure S34: Funnel plot of sensitive analysis: ADCS-ADL**


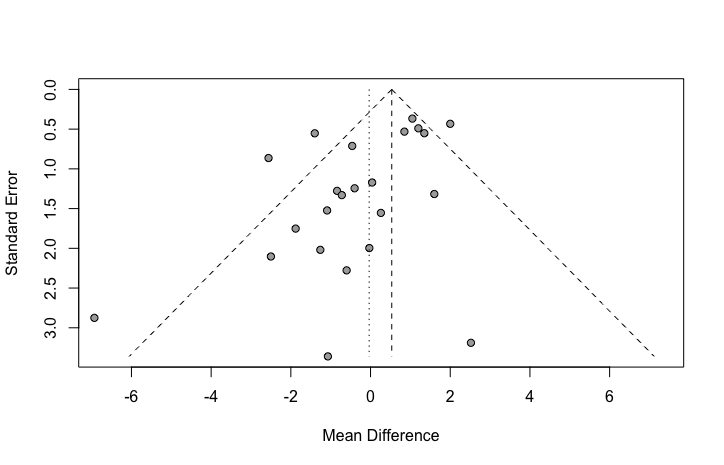


**Figure S35: Funnel plot of sensitive analysis: NPI**


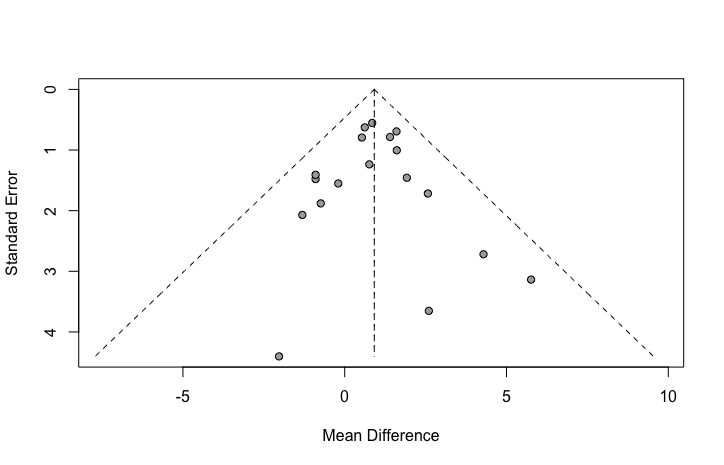


**Figure S36: Funnel plot of sensitive analysis: AEs**


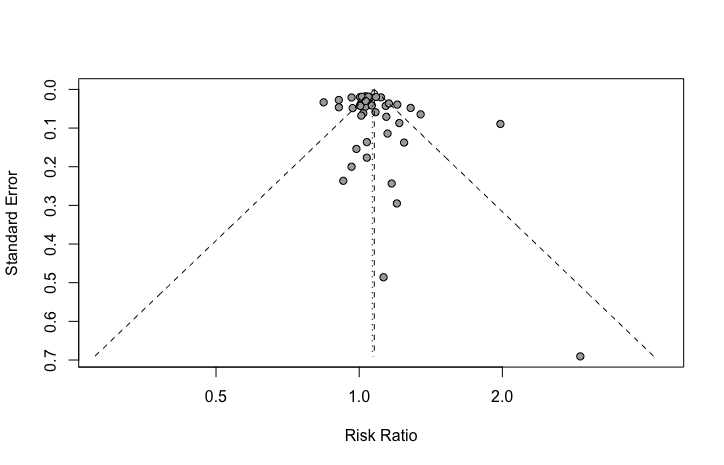


**Figure S37: Funnel plot of sensitive analysis: SAEs**


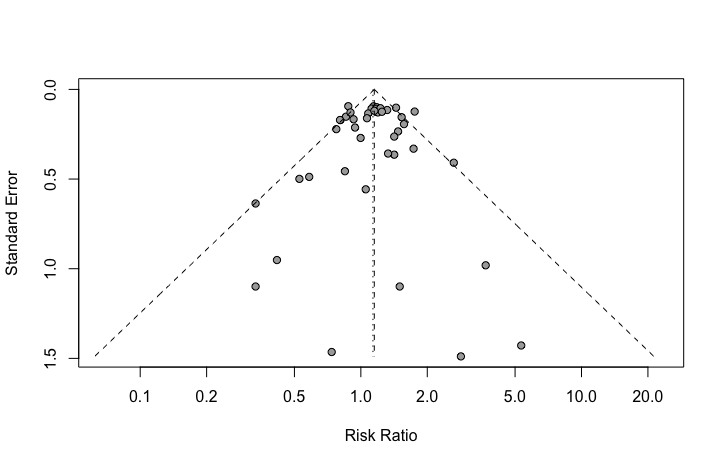


**Figure S38: Funnel plot of sensitive analysis: Death**


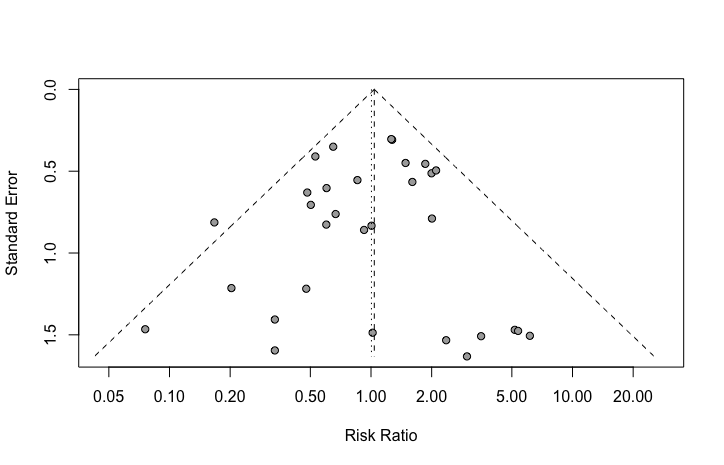

Supplement: Supplementary file 1 [file Data_Sheet_1.DOCX]
